# Cerium–quinone redox couples put under scrutiny†

**DOI:** 10.1039/d0sc04489j

**Published:** 2020-11-23

**Authors:** Uwe Bayer, Daniel Werner, Andreas Berkefeld, Cäcilia Maichle-Mössmer, Reiner Anwander

**Affiliations:** Institut für Anorganische Chemie, Eberhard Karls Universität Tübingen (EKUT) Auf der Morgenstelle 18 72076 Tübingen Germany reiner.anwander@uni-tuebingen.de [http://uni-tuebingen.de/syncat-anwander]

## Abstract

Homoleptic cerous complexes Ce[N(SiMe_3_)_2_]_3_, [Ce{OSi(O*t*Bu)_3_}_3_]_2_ and [Ce{OSi^i^Pr_3_}_3_]_2_ were employed as thermally robust, weakly nucleophilic precursors to assess their reactivity towards 1,4-quinones in non-aqueous solution. The strongly oxidizing quinones 2,3-dichloro-5,6-dicyano-1,4-benzoquinone (DDQ) or tetrachloro-1,4-benzoquinone (Cl_4_BQ) readily form hydroquinolato-bridged ceric complexes of the composition [(Ce^IV^L_3_)_2_(μ_2_-O_2_C_6_R_4_)]. Less oxidising quinones like 2,5-di-*tert*-butyl-1,4-benzoquinone (*t*Bu_2_BQ) tend to engage in redox equilibria with the ceric hydroquinolato-bridged form being stable only in the solid state. Even less oxidising quinones such as tetramethyl-1,4-benzoquinone (Me_4_BQ) afford cerous semiquinolates of the type [(Ce^III^L_2_(thf)_2_)(μ_2_-O_2_C_6_Me_4_)]_2_. All complexes were characterised by X-ray diffraction, ^1^H, ^13^C{^1^H} and ^29^Si NMR spectroscopy, DRIFT spectroscopy, UV-Vis spectroscopy and CV measurements. The species putatively formed during the electrochemical reduction of [Ce^IV^{N(SiMe_3_)_2_}_3_]_2_(μ_2_-O_2_C_6_H_4_) could be mimicked by chemical reduction with Co^II^Cp_2_ yielding [(Ce^III^{N(SiMe_3_)_2_}_3_)_2_(μ_2_-O_2_C_6_H_4_)][Co^III^Cp_2_]_2_.

## Abstract 2

*Para*-quinones reveal distinct reactivity towards homoleptic cerous silylamide and siloxide complexes depending on both their oxidizing power and the supporting ligand L.

## Graphical abstract

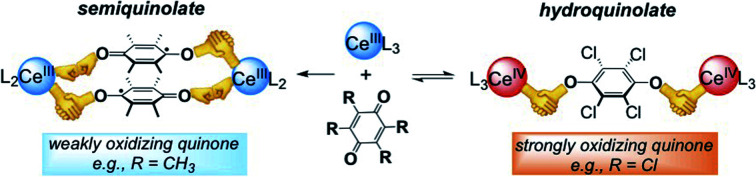

## Introduction

Quinones are multifunctional organic molecules exhibiting intriguing redox behaviour.^1,2^ Of particular note is their importance in biological electron–transfer processes (photosynthesis, respiration)^3^ and in industrial catalysis (anthraquinone process for hydrogen peroxide production).^4^ Quinones can engage in one or two electron redox processes involving the formation of either semiquinolates or hydroquinolates.^5^ Strikingly, the reduction potential of 1,4-benzoquinones (*para*-benzoquinones) can easily be modified by introducing electron-withdrawing or donating substituents into the benzene ring.^5,6^ As a consequence, tetrachloro-1,4-benzoquinone (chloranil, Cl_4_BQ) and even more so 2,3-dichloro-5,6-dicyano-1,4-benzoquinone (DDQ) emerged as efficient oxidants in organic synthesis.^7^ DDQ has been further successfully applied in photoredox catalysis.^8^ Moreover, anionic η^4^-1,4-benzoquinone manganese tricarbonyl features a quinoid π-complex, broadly used for the fabrication of supramolecular metal–organometallic coordination networks.^9^ Relatedly, deprotonated variants of 2,5-dihydroxy-1,4-benzoquinone (DHBQ) were shown to act as rigid ditopic linkers,^10^*e.g.*, to support the formation of pentagonal dodecahedral Ce_2_(H_2_O)_18_ cages or in permanently porous aluminium frameworks.^11^ DHBQ was also probed as a bridging redox-active ligand in bimetallic [LnCl_2_(thf)_3_]_2_(μ-bobq) (Ln = Y, Dy; bobq = 2,5-bisoxide-1,4-benzoquinolato) to build single-molecule magnets.^12^ More recently, the related semiquinolato radical-bridged dimeric complexes [LnCl_2_(thf)_3_(μ-Me_4_sq)_2_]_2_ (Ln = Y, Gd) were obtained by oxidation of the corresponding *in situ* formed hydroquinolate complexes with FeCl_3_.^13^ Semiquinolato-bridged scandium(III) species were reported to promote self-organised electron transfer from d-transition metals (Ir, Fe) to 1,4-quinones.^14,15^

Targeted metal-redox chemistry with quinones has been a recurring issue for the rare-earth-metal couples Ln(ii)/Ln(iii)^16^ and Ce(iii)/Ce(iv).^17^ Especially in the case of molecular cerium chemistry,^17^ its unique single-electron-transfer (SET) pathway has recently been extended beyond the traditional application of ceric ammonium nitrate (CAN; redox potential of 1.61 V *vs.* NHE) in organic synthesis^18^ to photoredox catalysis.^19^ On the other hand, redox protocols are known to provide efficient access to metalorganic Ce^IV^ complexes. Typically, such Ce^III^ → Ce^IV^ transformations are promoted by halogenating oxidants (*e.g.* C_2_Cl_6_, Ph_3_CCl, PhICl_2_, TeCl_4_, FcPF_6_, FcBF_4_, Ph_3_CBF_4_, Ph_3_CPF_6_, I_2_),^20^ silver salts (AgX, X = F, I, BF_4_, OTf)^21^ or dioxygen.^20*b*,22^

**Chart 1:**
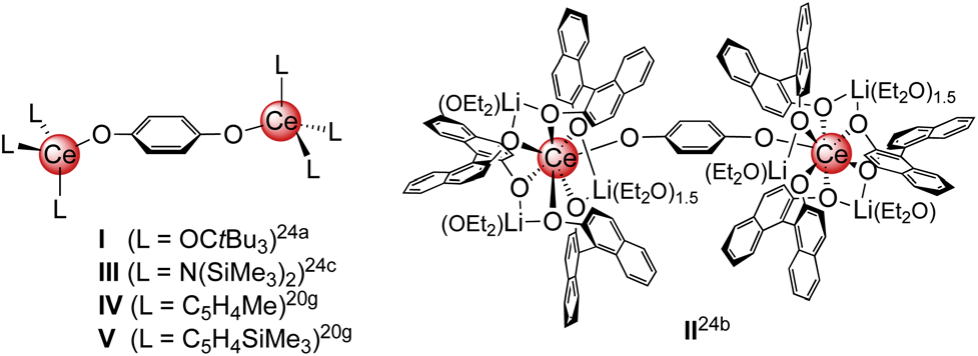
Structurally characterised dicerium(iv) hydroquinolate complexes (I–V),^20*g*,24^ obtained *via* oxidation of cerous precursor with BQ.

Archetypical 1,4-benzoquinone (BQ) has been established as a versatile oxidant for the synthesis of homoleptic ceric complexes CeL_4_ from cerous ate complexes [CeL_4_M(do)_*x*_] *via* tandem oxidation-ligand redistribution protocols (L = monoanionic ligand, M = alkali metal and do = donor solvent; separation of an alkali-metal hydro-/semiquinolate).^23^ In the presence of sterically demanding ligands L, BQ was also shown to form hydroquinolato (hq)–bridged ceric complexes of the general composition [L_3_Ce–OC_6_H_4_O–CeL_3_].^20*g*,24^ This very Ce^III^ → Ce^IV^ transformation was pioneered by Sen *et al.* in 1992, resulting in the isolation of [(*t*Bu_3_CO)_3_Ce(OC_6_H_4_O)Ce(OC*t*Bu_3_)_3_] (Chart 1, I).^24*a*^ In the same paper, the oxidation of Ce(OC*t*Bu_3_)_3_ with 2,6-di-*tert*-butyl-1,4-benzoquinone to the terminal Ce^IV^-semiquinolate radical (*t*Bu_3_CO)_3_Ce(O_2_C_6_H_2_*t*Bu_2_) was described as evidenced by ^1^H NMR and EPR spectroscopic measurements.^24*a*^ More recently, Schelter *et al.* reported on hq-bridged complex II resulting from the oxidation of cerous Ce(BINOlate)_3_(thf)Li_3_(thf)_4_ with 0.5 equivalents of BQ.^24*b*^ Similarly, our group synthesized [Ce{N(SiMe_3_)_2_}_3_]_2_(μ_2_-O_2_C_6_H_4_)^24*c*^ (III) and (CeCp^R^_3_)_2_(μ_2_-O_2_C_6_H_4_) (Cp^R^ = C_5_H_4_Me (IV) and C_5_H_4_(SiMe_3_) (V)).^20*g*^ In contrast, the reaction of BQ with [Ce(Me_2_pz)_3_]_*x*_ featuring the sterically less demanding and increasingly nucleophilic 3,5-dimethylpyrazolato ligand (Me_2_pz) led in fact to a transient Ce^IV^ hydroquinolate species (as indicated by the characteristic colour change), which, however, at ambient temperature was converted into the isolable trimetallic Ce^III^ complex Ce_3_(pchd)_2_(Me_2_pz)_5_(thf)_2_ (pchd = 1,4-bis(3,5-dimethylpyrazol-1-yl)cyclohex-2,5-diene-1,4-diolato).^23*c*^ Apparently, the new pchd ligand formed *via* 1,4-nucleophilic attack at bq by two adjacent Me_2_pz ligands. This nucleophilic reaction pathway could be prevented by using bulky *t*Bu groups on the pz ligand, but homolpetic Ce(*t*Bu_2_pz)_4_ was formed as the main ceric product *via* irreversible ligand rearrangement.^23*c*^

As such, cerium–1,4-benzoquinone couples have revealed distinct redox chemistry, we became curious about as to what extent such redox transformations are affected by both the type of 1,4-benzoquinone oxidant and the molecular Ce^III^ precursor. The present study uncovers some unexpected correlation between Ce^IV^–hydroquinolato stabilisation and quinone oxidant strength, as well as a new path to *p*-semiquinolato–radical-bridged rare-earth-metal complexes.

## Results and discussion

### Molecular redox precursors

**Scheme 1:**
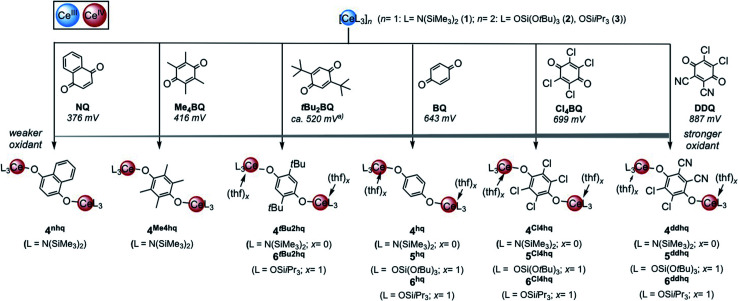
Oxidation of trivalent cerium complexes with 1,4-quinone derivatives under formation of hydroquinolato-bridged ceric complexes. To enable better assessment of the relative oxidation ability 2e^−^/2H^+^ reductions potentials are given in mV *versus* NHE according to ref. 5. (*a*) Calculated value.

The quinones used in this study comprise 2,3-dichloro-5,6-dicyano-1,4-benzoquinone (DDQ), tetrachloro-1,4-benzoquinone (Cl_4_BQ), 1,4-benzoquinone (BQ), tetramethyl-1,4-benzoquinone (Me_4_BQ), 2,5-di-*tert*-butyl-1,4-benzoquinone (*t*Bu_2_BQ), 1,4-naphthoquinone (NQ), and 9,10-anthraquinone (AQ). All are commercially available and were selected according to their reduction potentials spanning a *E*^0^ range of 89 to 887 mV (2e^−^/2H^+^, *vs.* NHE, *cf.*, Scheme 1).^5,25^ The cerous precursors were chosen according to the criteria solubility, weak nucleophilicity, proven access to the tetravalent state, and a stabilizing effect on the latter. Furthermore, the use of sterically bulky ligands was assumed to minimise the occurrence of ligand redistribution reactions. Accordingly, homoleptic Ce[N(SiMe_3_)_2_]_3_ (1) appeared to be an ideal benchmark system.^24*c*^ After additional investigations into the respective pyrazolate chemistry, the abovementioned [Ce(R_2_pz)_3_] (R = Me, *t*Bu) were discarded because of persisting alternative reaction pathways like 1,4-nucleophilic attack of BQ by Me_2_pz and ligand redistribution (formation of Ce(*t*Bu_2_pz)_4_).^23*c*^ The new pyrazolate studies clearly confirmed that steric hindrance of both the pyrazolato ligand and the 1,4-benzoquinone can minimise/counteract such undesired reactions, but the formation of product mixtures seems inevitable. Products crystallised from these reactions include minor amounts of ceric [Ce(*t*Bu_2_pz)_3_(thf)]_2_(Me_4_hq) or a cerous product of partial pyrazolyl-promoted nucleophilic attack Ce_3_(bpad)(pasq)(Me_2_pz)_6_(thf) (bpad = 1,4-bis(3,5-dimethylpyrazol-1-yl)anthra-1,4-diolato; pasq = 1-(3,5-dimethylpyrazol-1-yl)anthra-1,4-semiquinolato) (84%) mixed with semiquinolate [Ce(Me_2_pz)_2_(thf)_2_(asq)]_2_ (asq = anthra-semiquinolato; *cf.* ESI† for structural details). The use of Ce^III^ halides was discarded mainly for solubility issues.

**Fig. 1:**
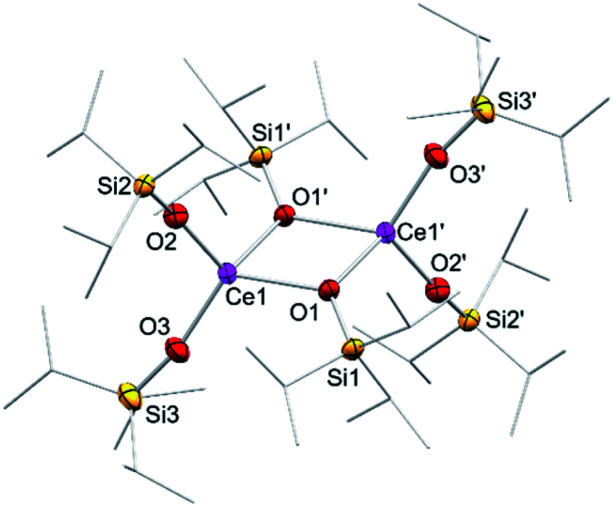
Crystal structure of [Ce{OSi^i^Pr_3_}_3_]_2_ (3). Ellipsoids are shown at the 50% probability level. Hydrogen atoms are omitted for clarity. Selected interatomic distances [Å] and angles [°]: Ce1–O1 2.3951(12), Ce1–O2 2.1659(14), Ce1–O3 2.1671(14); Ce1–O2–Si2 163.03(9), Ce1–O3–Si3 167.40(10).

In addition to silylamide 1, the siloxide derivatives [Ce{OSi(O*t*Bu)_3_}_3_]_2_ (2)^21*d*,26^ and [Ce(OSi^i^Pr_3_)_3_]_2_ (3) were assessed as suitable cerous precursors. Complexes 2 and 3, with and without intramolecular donor site, respectively, were readily obtained in pure form *via* protonolysis of 1 with the corresponding silanol.^26^ The crystal structure of the new complex 3 revealed a dimeric arrangement with two μ_2_-bridging and four terminal siloxy groups (Fig. 1), similar to that found for tris(*tert*-butoxy)siloxy congener 2 or [Ce(OSiPh_3_)_3_]_2_ ^27^ or [Ce(OCH*t*Bu_2_)_3_]_2_.^28^ The Ce–O_terminal_ (2.1659(14) and 2.1671(14) Å) and the Ce–O_μ_2__ distances (2.3951(12) and 2.4030(12) Å) of 3 are slightly shorter than those in 2 (Ce–O_terminal_ 2.202(3), 2.186(3) Å; Ce–O_μ2_ 2.532(2) Å) and [Ce(OSiPh_3_)_3_]_2_ (Ce–O_terminal_ 2.141(7), 2.185(6) Å; Ce–O_μ2_ 2.345(6), 2.583(5) Å) reflecting the lower coordination number (CN 4 *vs.* 5), but slightly longer than in [Ce(OCH*t*Bu_2_)_3_]_2_ (Ce–O_terminal_ 2.142(2), 2.152(3) Å; Ce–O_μ2_ 2.363(3) Å).^28^ The ^1^H NMR spectrum of 3 in C_6_D_6_ shows two singlets at −28.82 and −17.23 ppm for the μ_2_-OSi^i^Pr_3_ groups and two singlets at 6.46 and 9.09 ppm for the terminal siloxy ligands indicating a non-fluxional dimeric species in non-coordinating solvents. When recorded in THF-*d*_8_, only two signals for the OSi^i^Pr_3_ groups appeared, in accordance with the formation of a monomeric adduct [Ce{OSi^i^Pr_3_}_3_(thf-*d*_8_)_*x*_].

### Quinone oxidation of Ce[N(SiMe_3_)_2_]_3_ (1)

**Fig. 2:**
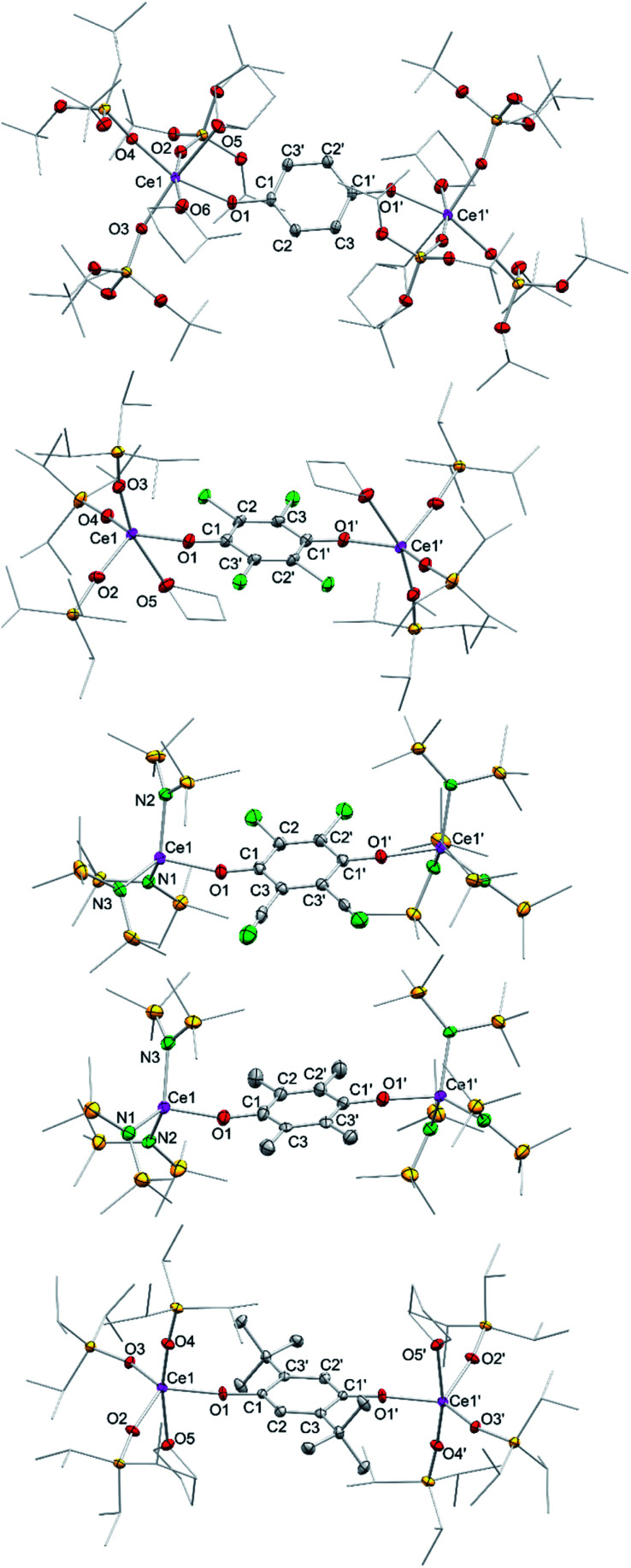
Crystal structures of [Ce{OSi(O*t*Bu)_3_}_3_(Me-thf)_2]2_(μ_2_-O_2_C_6_H_4_) (5^hq^·2MeTHF), [Ce{OSi^i^Pr_3_}_3_(thf)]_2_(μ_2_-O_2_C_6_Cl_4_) (6^Cl4hq^), [Ce{N(SiMe_3_)_2}3]2_(μ_2_-O_2_C_6_Cl_2_CN_2_) (4^ddq^), [Ce{N(SiMe_3_)_2}3]2_(μ_2_-O_2_C_6_Me_4_) (4^Me4hq^), and [Ce{OSiiPr_3}3_(thf)]_2_(μ_2_-O_2_C_6_*t*Bu_2_H_2_) (6*^t^*^Bu2hq^) (from top down). Ellipsoids are shown at the 50% probability level. Hydrogen atoms, disordered ligands and lattice solvents are omitted for clarity. Selected interatomic distances for 5^hq^, 6^Cl4hq^, 4^ddq^, and 4^Me4hq^ are given in Tables 1 and 2. Selected bond lengths for 6*^t^*^Bu2hq^ [Å]: Ce1–O1 2.109(3), Ce1–O2 2.104(3), Ce1–O3 2.097(3), Ce1–O4 2.107(3), C1–C2 1.391(6), C2–C3 1.391(6), C1–C3′ 1.405(6), C1–O1 1.350(5).

Treatment of Ce[N(SiMe_3_)_2_]_3_ (1) with each 0.5 equivalents of DDQ, Cl_4_BQ, Me_4_BQ, *t*Bu_2_BQ and NQ, in a mixture of toluene and *n*-hexane, immediately led to a colour change from yellow to dark brown. Upon recrystallisation from toluene/*n*-hexane mixtures it was possible to isolate the hydroquinolato-bridged complexes [Ce{N(SiMe_3_)_2_}_3_]_2_(μ_2_-O_2_C_6_Cl_4_) (4^Cl4hq^), [Ce{N(SiMe_3_)_2_}_3_]_2_(μ_2_-O_2_C_6_Cl_2_(CN)_2_) (4^ddhq^), [Ce{N(SiMe_3_)_2_}_3_]_2_(μ_2_-O_2_C_6_Me_4_) (4^Me4hq^), [Ce{N(SiMe_3_)_2_}_3_]_2_(μ_2_-O_2_C_6_*t*Bu_2_H_2_) (4*^t^*^Bu2hq^) and [Ce{N(SiMe_3_)_2_}_3_]_2_(μ_2_-O_2_C_10_H_6_) (4^nhq^) in very good crystalline yields of 71 to 90% (Scheme 1). The crystal structures of the new complexes 4^xhq^ are isostructural to the previously reported derivative 4^hq^^24*c*^ and only differ in the bridging hq linker (Fig. 2). The Ce1–O1 distances of 2.084(6) to 2.173(2) Å (for a full list of interatomic distances see Table 1) are in the same range as found for other hq-bridged cerium complexes (2.086(10)–2.143(5) Å).^20*g*,24^ Likewise, the Ce1–N bond lengths compare well to other Ce^IV^ silylamides like [Ce{N(SiMe_3_)_2_}_3_]_2_(μ_2_-O_2_C_6_H_4_) (2.2388(14)–2.2487(14) Å),^24*c*^ Ce[N(SiMe_3_)_2_]_3_Cl (2.217(3) Å),^20*a*^ and Ce[N(SiHMe_2_)_2_]_4_ (2.2378(11)–2.2574(11) Å).^20*d*^ Also, the C–C distances of the hq linker converge as the expected aromatic ring is formed and the C–O distances of 1.318(3) to 1.378(3) Å corroborate the formation of C–O single bonds.

**Table 1:** Selected analytical data of complexes 4^hq^, 4^Cl4hq^, 4^ddhq^, 4^Me4hq^, 4*^t^*^Bu2hq^, 4^nhq^. Interatomic distances are given in [Å], angles in [°], chemical shifts in [ppm], *μ*_eff_ in [BM], UV/Vis absorption band in [nm], and *E*_pc_/*E*_pa_ in [V *vs.* Fc/Fc^+^]

Complex	4^hq^^24*c*^	4^Cl4hq^	4^ddhq^	4^Me4hq^	4*^t^*^Bu2hq^	4^nhq^
Ce1–O1	2.0895(13)	2.149(4)	2.1731(15)	2.082(6)	2.117(2)	—
Ce1–N1	2.2388(14)	2.265(5)	2.2206(18)	2.280(7)	2.235(2)	—
Ce1–N2	2.2398(15)	2.230(5)	2.2112(18)	2.229(7)	2.246(2)	—
Ce1–N3	2.2487(14)	2.244(5)	2.2467(19)	2.238(7)	2.259(2)	—
C–C_arom_	1.387(3)–1.399(2)	1.383(8)–1.392(8)	1.390(4)–1.407(3)	1.390(11)–1.408(12)	1.388(4)–1.405(4)	—
C1–O1	1.356(2)	1.324(7)	1.318(3)	1.366(10)	1.378(3)	—
Ce1–O1–C1	173.13(11)	156.1(4)	161.02(15)	162.4(6)	146.79(18)	—
^1^H NMR^a^	0.43	0.45	0.45	0.43^d^	0.49	0.44
^13^C{^1^H} NMR^a^	—	—	5.6	—	—	5.6
^29^Si{^1^H} NMR^a^	—	−8.1	−7.3	−8.8	—	−8.1
*μ*_eff_	0.67	0.68	0.59	0.89	—	1.19
UV-Vis absorption bands^b^	485	319/518	384/511	362/411/681	—	339/425/474/677
*E*_pc_ c	−0.699/−0.966	−0.415/−0.624	−0.546	—	—	—
*E*_pa_ c	−0.558	−0.290	−0.1685	—	—	—
*E*_1/2_	−0.76	−0.46	−0.36			
Δ*E*	0.408	0.334	0.377	—	—	—

a NMR spectra recorded in C_6_D_6_.

b UV-Vis spectra recorded in toluene.

c Determined in THF using *c*(analyte) = 2 mM and *c*(electrolyte) = 0.1 M and a scan rate of 50 mV s^−1^.

d Determined in toluene-*d*_8_ at 0 °C.

**Scheme 2:**
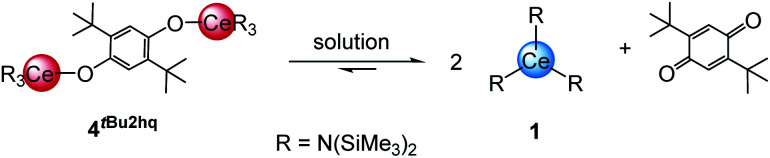
Equilibrium between 4*^t^*^Bu2hq^ and reformation of reactants in solution and solid state.

The ^1^H NMR spectra of compounds 4^xhq^ in C_6_D_6_ show singlets for the trimethylsilyl (TMS) groups at 0.43 to 0.45 ppm along with signals for the bridging hydroquinolato moieties. Further, the ^13^C{^1^H} NMR spectra of 4^ddhq^ and 4^nq^ display a singlet for the TMS groups at 5.6 ppm and signals in the aromatic region for the different hydroquinolates, indicative of a successful reduction of the respective benzoquinone derivatives. The characterisation of 4*^t^*^Bu2hq^ in solution (C_6_D_6_) was not feasible, due to the prevailing equilibrium shown in Scheme 2, and ready back-formation of 1 and 2,5-di-*tert*-butyl-1,4-benzoquinone.

While the ^1^H NMR spectrum of 4*^t^*^Bu2hq^ primarily shows signals for the starting materials and only minor product signals, its DRIFT spectrum indicated the absence of any strong C=O absorption band, and therefore the stability of 4*^t^*^Bu2hq^ in the solid state (see Fig. S10 and S45 in ESI†). In contrast, complexes 4^hq^, 4^Cl4hq^ and 4^ddhq^ derived from the stronger oxidizing quinones are very stable in the solid state and in solution. This fits again well with the already pronounced instability of 4^Me4hq^ and 4^nhq^ which slowly decompose in *n*-hexane and toluene at ambient temperature and rapidly undergo decomposition in THF. Tracking of the progress of the decomposition by ^1^H NMR spectroscopy revealed the formation of 1 and other paramagnetic Ce^III^ species which, however, could not be identified. The progressing decomposition can also be seen in the ligand-to-metal charge transfers observed in the UV-Vis spectra (Fig. S68, ESI†). As the spectra of 4^hq^, 4^Cl4hq^ and 4^ddhq^ show mainly one strong absorption band at around 500 nm (*ε* > 5060 L mol^−1^ cm^−1^), the spectra of 4^Me4hq^ and 4^nhq^ show several absorption bands with significantly lower intensities (*ε* < 4400 L mol^−1^ cm^−1^) indicative of Ce^III^ species and therefore redox decomposition of the compounds.

All attempts to isolate putative 4^ahq^, derived from the weakest oxidising quinone under study, namely 9,10-anthraquinone (*E*^0^ = 89 mV; 2e^−^/2H^+^, *vs.* NHE),^5^ were unsuccessful with the reaction mixtures showing no colour change immediately after addition of the anthraquinone. However, a colour change from yellow to green occurred after two days and the respective ^1^H NMR spectrum showed multiple paramagnetic signals.

### Quinone oxidation of siloxides [Ce{OSi(O*t*Bu)_3_}_3_]_2_ (2) and [Ce{OSi^i^Pr_3_}_3_]_2_ (3)

Reacting cerous siloxides 2 and 3 with the selected quinones in THF immediately gave a colour change of the reaction mixtures (from colourless to: dark purple (BQ), dark red (Cl_4_BQ), dark yellow/orange (DDQ), pale purple (*t*Bu_2_BQ), pale blue (Me_4_BQ), pale green (NQ)). The ceric compounds [CeL_3_(thf)]_2_(μ_2_-O_2_C_6_H_4_) (5^hq^, 6^hq^), [CeL_3_(thf)]_2_(μ_2_-O_2_C_6_Cl_4_) (5^Cl4hq^, 6^Cl4hq^), [CeL_3_(thf)]_2_(μ_2_-O_2_C_6_Cl_2_(CN)_2_) (5^ddq^, 6^ddq^), with L = OSi(O*t*Bu)_3_ or OSi^i^Pr_3_ derived from quinones with a relatively strong oxidising effect were successfully isolated from these reactions (Scheme 1).

However, the weakly oxidizing quinones Me_4_BQ and NQ did not lead to tetravalent cerium species, as indicated by the detection of only paramagnetic signals in the ^1^H NMR spectra (for an example of such a ^1^H NMR spectrum, see Fig. S34 in the ESI;† formation of semiquinolates, *vide infra*). The accessible complexes 5 and 6 were obtained in moderate to good crystalline yields of 42 to 71% upon recrystallisation from THF or THF/Et_2_O mixtures. Crystals suitable for XRD analysis were obtained for complexes 5^hq^, 5^Cl4hq^, 6^Cl4hq^, 6^ddhq^ and 6*^t^*^Bu2hq^, revealing the same structural motif as complexes 4, that is two CeL_3_ moieties connected *via* a hydroquinolato linker (Fig. 2).

Strikingly, the ^1^H NMR spectrum of 6*^t^*^Bu2hq^ indicated the existence of an equilibrium similar to that of ceric 4*^t^*^Bu2hq^ (*cf.*Scheme 2). However, along with the reactants 3 and *t*Bu_2_BQ additional signals assignable to distinct dia- and paramagnetic decomposition products were detected. Further, the crystal structures of complexes 5 and 6 show that the cerium atoms are additionally coordinated by THF donor molecules. The Ce1–O_siloxide_ distances of 2.066(2) to 2.1534(10) (see Table 2 for a complete list of interatomic distances) compare well to other ceric siloxides like Ce{OSi(O*t*Bu)_3_}_4_ (2.089(2)–2.157(2) Å ^26^ and 2.084–2.160 Å ^21*d*^) or Ce{OSiPh_3_}_4_(dme) (2.098(1)–2.133(1) Å).^29^ Also, as seen for the silylamides 4, the Ce1–O_hq_ distances of 2.1244(10) to 2.2325(16), as well as the C–C and C–O distances underline the formation of an aromatic hq linker.^20*g*,24^^1^H NMR spectroscopic measurements also validate the formation of Ce^IV^ species, showing a sharp singlet for the *tert*-butyl groups and a doublet plus a septet for the iso-propyl groups depending on the siloxy co-ligand.

**Table 2:** Selected analytical data of complexes 5^hq^, 5^Cl4hq^, 5^ddq^, 6^hq^, 6^Cl4hq^, 6^ddq^. Interatomic distances are given in [Å], angles in [°], chemical shifts in [ppm], *μ*_eff_ in [BM], UV/Vis absorption band in [nm], and *E*_pc_/*E*_pa_ in [V *vs.* Fc/Fc^+^]

Complex	5^hq^	5^Cl4hq^	5^ddq^	6^hq^	6^Cl4hq^	6^ddq^
Ce1–O1	2.1244(10)	2.184(3)	—	—	2.207(2)	2.2325(16)
Ce1–O2	2.1534(10)	2.091(3)	—	—	2.095(2)	2.0841(16)
Ce1–O3	2.1334(11)	2.094(3)	—	—	2.066(2)	2.0710(17)
Ce1–O4	2.1396(11)	2.104(3)	—	—	2.080(2)	2.091(2)
C–C_arom_	1.390(2)–1.395(2)	1.373(8)–1.406(5)	—	—	1.380(4)–1.407(4)	1.379(5)–1.415(3)
C1–O1	1.3540(17)	1.326(4)	—	—	1.322(3)	1.316(3)
Ce1–O1–C1	151.76(10)	140.0(2)	—	—	138.54(18)	144.85(14)
^1^H NMR^a^	1.36	1.36	1.36		1.12/1.05	1.12/1.05
^13^C{^1^H} NMR^a^	72.5/32.6	72.8/32.4	73.0/32.5		18.0/14.0	19.1/15.1
^29^Si{^1^H} NMR^a^	−103.2	−104.6	−105.3	7.0	9.6	10.7
*μ*_eff_	0.82	0.54	0.60	0.68	0.50	0.66
UV-Vis absorption band^b^	369/622	493	384/450	526^c^	511	381/470
*E*_pc_ d	−1.7855	−1.414	−1.353	−1.116/−1.816	−1.580	−1.149/−1.484
*E*_pa_ d	−0.3625	−0.521	0.130	−1.273/−0.817	−0.729	−0.751
Δ*E*	1.416	0.893	1.483	0.999	0.851	0.733

a NMR spectra recorded in THF-*d*_8_.

b Spectra recorded in toluene.

c Spectra recorded in THF.

d Determined in THF using *c*(analyte) = 2 mM and *c*(electrolyte) = 0.1 M and a scan rate of 50 mV s^−1^.

### Electrochemical investigation of complexes 4^xhq^, 5^xhq^ and 6^xhq^

Cyclic voltammetry (CV) measurements of complexes 4^xhq^, 5^xhq^ and 6^xhq^ have been conducted at ambient temperature in 0.2 mM solutions in THF and 0.1 M [*n*Pr_4_N][B(C_6_H_3_(CF_3_)_2_-3,5)_4_] as a support electrolyte, and referenced *vs.* Fc/Fc^+^. Due to the low stability of compounds 4^Me4hq^, 4*^t^*^Bu2hq^ and 4^nhq^ in polar solvents CV measurements of these complexes were not feasible. Most of the CV measurements revealed successive quasireversible (4) or irreversible (5/6) Ce^IV^ → Ce^III^ reduction steps, but badly resolved (for *E*_pc_ values see Tables 1 and 2). The detection of two closely adjacent redox events (*E*_pc_ values) in some cyclic voltammograms may correspond to a successive reduction/oxidation of the cerium centres. Similar features were also described for the hq-bridged Ce(iv)–BINOLate complex II.^24*b*^ All complexes under study display redox processes with a large separation of *E*_pc_ and *E*_pa_ (Δ*E* ≈ 0.6 V for 4; 1.5 V for 5 and 1.0 V for 6).

**Fig. 3:**
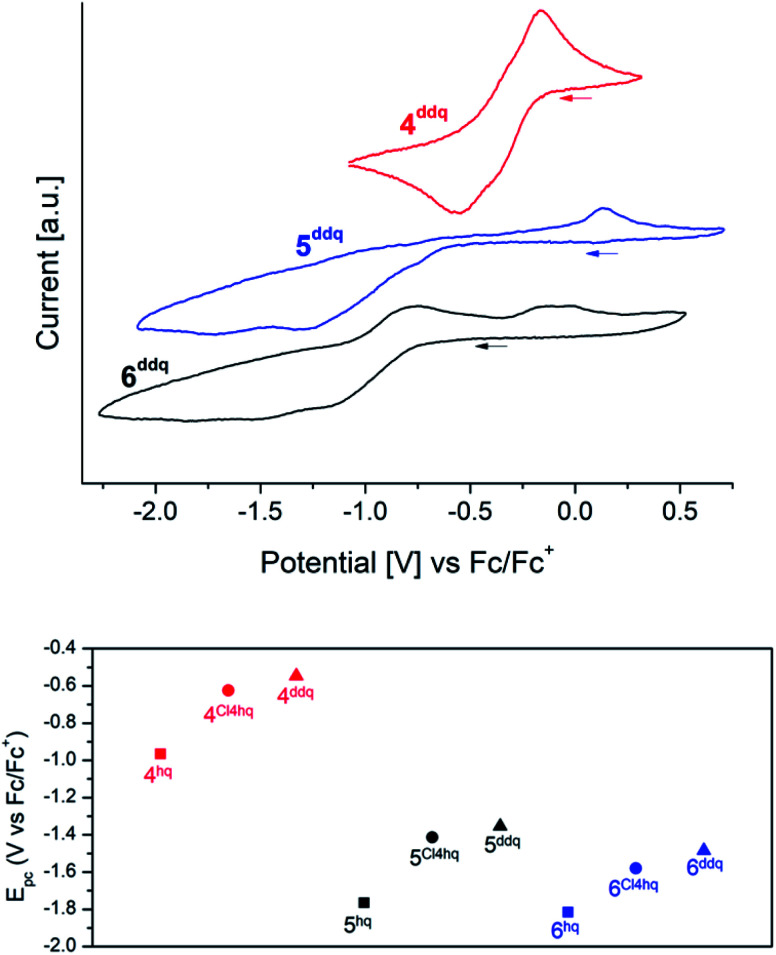
Top: Stacked cyclic voltammograms of complexes 4^ddq^ (red), 5^ddq^ (blue), 6^ddq^ (black) in THF (*v* = 50 mV s^−1^; c(analyte) = 2 mM; c([*n*Pr_4_N][B(C_6_H_3_(CF_3_)_2_-3,5)_4_]) = 0.1 M). Bottom: comparison of the second reduction potentials of complexes 4 (red), 5 (black), and 6 (blue) in THF *vs.* Fc/Fc^+^. Squares: complexes with bridging 1,4-hydroquinolates; cycles: complexes with bridging tetrachloro-1,4-hydroquinolates; triangles: complexes with bridging 2,3-dichloro-5,6-dicyano-1,4-hydroquinolates.

Representatively, the cyclic voltammograms of the DDQ-functionalized Ce^III^/Ce^IV^ redox couples are depicted in Fig. 3 (top graphic). The silylamide complexes 4 gave reduction potentials similar to those reported for halogenido-functionalised ceric complexes Ce[N(SiMe_3_)_2_]_3_X (*E*_1/2_ = −0.56 (X = F), −0.30 (X = Cl), −0.31 (X = Br)) with *E*_1/2_ values of −0.46 V for 4^Cl4hq^ and −0.36 V for 4^ddhq^.^30^ Only 4^hq^ with *E*_1/2_ = −0.76 V gave a significantly higher stabilisation by 0.20 V. The extra-large separation of the reduction/oxidation events observed for the siloxide complexes 5 and 6 had been noticed previously for rare-earth-metal siloxides and was assigned to oxidation-state-dependent ligand reorganisation processes.^31^

Stabilisation of the tetravalent oxidation state of cerium in complexes 4, 5, and 6 increases in the order of N(SiMe_3_)_2_ < OSi(O*t*Bu_3_)_3_ < OSi^i^Pr_3_ as co-ligand (Fig. 3/bottom) which is in accordance with previous findings.^20*f*,26,30,31*c*^ Surprisingly, the stabilisation of Ce^IV^ proceeds in reverse order of the oxidation potential of the 1,4-quinones under study giving the most stable complexes for the hydroquinolato-bridged complexes and the least stable compounds for its 2,3-dichloro-5,6-dicyano-hydroquinolato congeners. A reason for this trend could be the increasingly electron-deficient nature of the aromatic hydroquinolato linkers due to the large −I effect of the substituents. The Ce^IV^ oxidation state can be stabilised by increasing donor strength of the ligands.^32^ Based on this, it seems surprising that isolable complexes 4^Me4hq^ and 4^nhq^, derived from weakly oxidizing quinones, are not stable in solution at ambient temperature. This might be a result of another reaction pathway preferred after formation of the hydroquinolato-bridged Ce^IV^ complexes (like following up redox processes and the formation of Ce^III^ semiquinolates, *cf.* vide infra).

### Reduction of silylamide 4^hq^ with cobaltocene

**Scheme 3:**
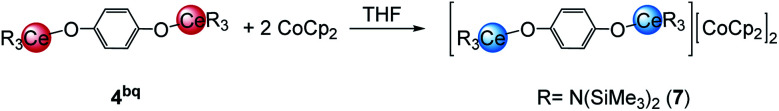
Reduction of 4^bq^ with two equivalents of CoCp_2_.

**Fig. 4:**
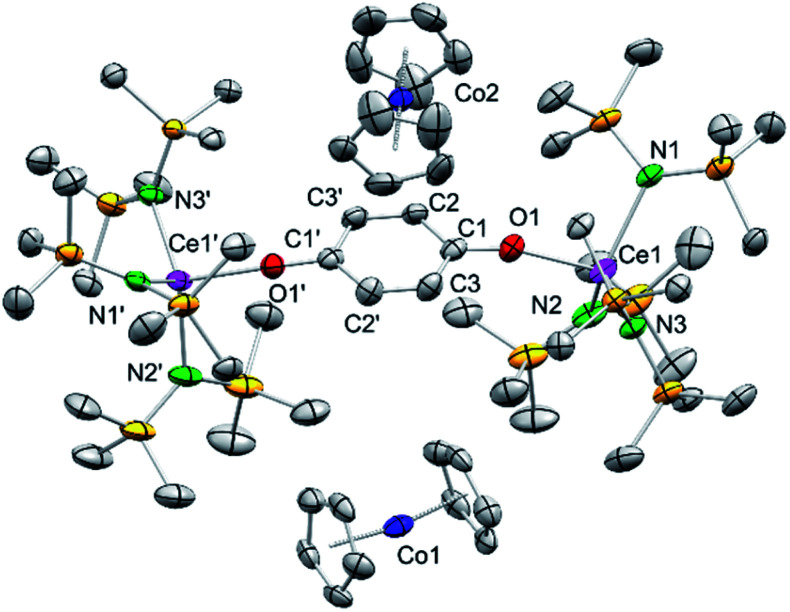
Crystal structure of [(Ce{N(SiMe_3_)_2_}_3_)_2_(μ_2_-O_2_C_6_H_4_)][CoCp_2_]_2_ (7). Ellipsoids are shown at the 50% probability level. Hydrogen atoms, disordering of the cyclopentadienyl ligands and lattice THF are omitted for clarity. Selected interatomic distances [Å]: Ce1–N1 2.420(2), Ce1–N2 2.402(2), Ce1–N3 2.418(2), Ce1–O1 2.202(2), C1–O1 1.344(4), C1–C2 1.391(4), C1–C3 1.392(4), C2–C3′ 1.392(4).

Having investigated the electrochemical reduction of compounds 4, 5 and 6, the chemical reduction with cobaltocene (CoCp_2_) (−1.31 V *vs.* Fc/Fc^+^ in DME)^2*a*^ was attempted, as it has already been shown to engage in such reductions.^31*a*,33^ Accordingly, treatment of a solution of 4^hq^ in THF with two equivalents of CoCp_2_ resulted in a colour change from dark brown to pale yellow (Scheme 3). The ^1^H NMR spectrum of the reaction mixture showed complete consumption of CoCp_2_ and only broadened signals indicating the formation of a paramagnetic Ce^III^ species. Crystallisation from a concentrated THF-*d*_8_ solution at −40 °C gave light brown crystals of the composition [(Ce{N(SiMe_3_)_2_}_3_)_2_(μ_2_-O_2_C_6_H_4_)][CoCp_2_]_2_ (7) (Fig. 4).

Complex 7 shows the same structural motif as 4^hq^ but is flanked by two cobaltocenium cations. Compared to 4^hq^, the Ce–N and Ce1–O1 distances are elongated by approximately 0.19 Å as expected for the larger Ce^III^ ion size.^34^ On the contrary, the bonding parameters within the bridging hydroquinolato linker did not change, further corroborating a cerium-borne redox chemistry. Reacting 4^hq^ with one equivalent of CoCp_2_ did not lead to a mixed Ce^III/IV^ complex but gave a mixture of 50% of 7 and 50% of unreacted starting material.

The reactions of CoCp_2_ with other complexes 4 to 6 in THF-*d*_8_ showed immediate decolourisation of the solution while the ^1^H NMR spectra of the reaction mixtures displayed only paramagnetic signals (for an example, see Fig. S33 in the ESI†). However, the isolation of additional reduced species similar to 7 was not successful.

### Cerium semiquinolates

**Scheme 4:**
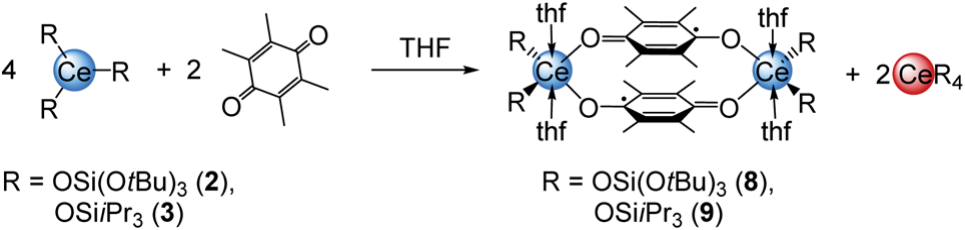
Formation of cerous semiquinolates 8 and 9 from the reaction of 2 or 3 with Me_4_BQ.

A closer look at the reactions of cerous siloxides 2 and 3 with the weakly oxidising quinone Me_4_BQ (which did not produce any tetravalent cerium species; *vide supra*) revealed another important detail of the cerium–quinone redox system. Treatment of 2 or 3 with 0.5 equivalents of Me_4_BQ led to a colour change from colourless to light blue. Upon recrystallisation from THF dark blue crystals suitable for X-ray diffraction could be grown and were identified as cerous semiquinolates [CeL_2_(thf)_2_]_2_(μ_2_-O_2_C_6_Me_4_)_2_ (with L = OSi(O*t*Bu)_3_ (8) or OSi^i^Pr_3_ (9)) (Scheme 4).

Examining the reaction mixtures by ^1^H NMR spectroscopy in THF-*d*_8_ showed, besides paramagnetic signals for 8 and 9, a sharp singlet at 1.39 ppm (for 8) or a doublet plus a septet at 1.13 and 1.04 ppm (for 9), indicating the formation of homoleptic Ce[OSi(O*t*Bu)_3_]_4_ or [Ce(OSi^i^Pr_3_)_4_], respectively, as a result of the one-electron reduction of Me_4_bq followed by ligand redistribution. Crucially, such a reaction pathway seems unfeasible for complexes 4, since putative homoleptic “Ce[N(SiMe_3_)_2_]_4_” is unknown.^20*a*^ Emergent kinetic constraints in the case of ceric complexes 4 were also suggested by the redox behaviour of [Ce{N(SiHMe_2_)_2_}_3_]_2_ derived from a less bulky silylamido ligand. Accordingly, the cerous bis(dimethylsilyl)amide complex was treated with one equivalent of both BQ and Me_4_BQ in THF-*d*_8_ and C_6_D_6_ (see Fig. S38–S41, ESI†). The ^1^H NMR spectra of these reactions suggest the formation of a tetravalent species of the composition “[Ce{N(SiHMe_2_)_2_}_3_]_2_(μ_2_-O_2_C_6_R_4_)”. However, the ceric products appear to be unstable in solution at ambient temperature. In C_6_D_6_, the formation of Ce[N(SiHMe_2_)_2_]_4_ was observed in the reaction with BQ as well as other insoluble products. In THF-*d*_8_, the resulting product seemed more stable but after 24 h in solution also traces of decomposition products were found. The Me_4_BQ reaction in C_6_D_6_ also indicated successful oxidation, however, after 24 h the ^1^H NMR spectrum revealed signals for trivalent decomposition products as well as traces of Ce[N(SiHMe_2_)_2_]_4_. In THF-*d*_8_, the putatively formed hydroquinolate complex was even less stable, showing signals for trivalent by-products directly after addition of Me_4_BQ. In addition, the stability of 4^bq^ was investigated in THF-*d*_8_ showing small amounts of decomposition products like Ce[N(SiMe_3_)_2_]_3_ after 24 h (Fig. S42, ESI†).

**Fig. 5:**
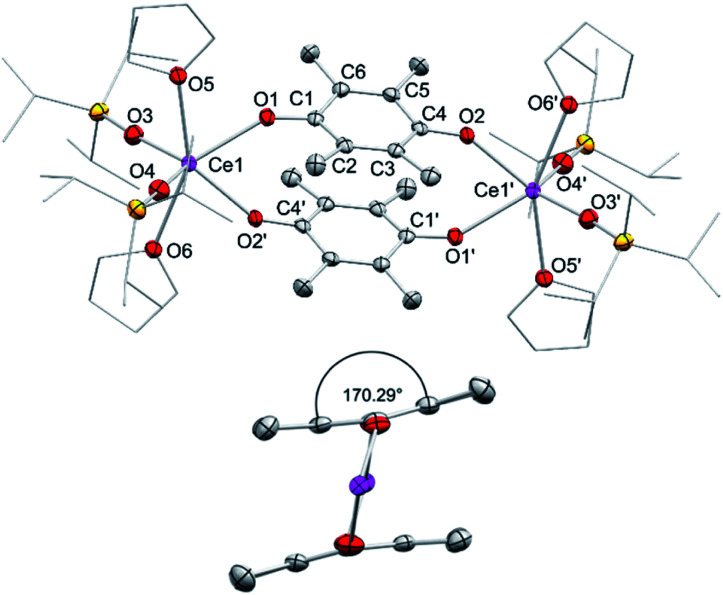
Top: Crystal structure of [Ce{OSi^i^Pr_3_}_2_(thf)_2_]_2_(μ_2_-O_2_C_6_Me_4_)_2_ (9). Ellipsoids are shown at the 50% probability level. Hydrogen atoms are omitted for clarity. Selected interatomic distances [Å]: Ce1–O1 2.446(7), Ce1–O2 2.422(7), Ce1–O3 2.219(9), Ce1–O4 2.237(6), Ce1–O5 2.648(7), Ce1–O6 2.644 (7), C1–O5 1.306(5), C4–O6 1.307(5), C1–C2 1.458(6), C2–C3 1.401(6), C3–C4 1.449(8), C4–C5 1.463(6), C5–C6 1.388(6), C1–C6 1.463(8). Bottom: side view of [(Ce{OSi^i^Pr_3_}_2_(thf)_2_)_2_(μ_2_-O_2_C_6_Me_4_)_2_] (9).

Regrettably, purification of complexes 8 and 9 was impeded by co-crystallisation with the ceric by-products CeL_4_. The crystal structures of 8 and 9 revealed two six-coordinate cerium atoms surrounded by two siloxy ligands, two THF donor molecules and two bridging tetramethyl semiquinolato moieties (Fig. 5). The Ce1–O_silanolato_ distances are elongated by about 0.1 Å compared to the respective tetravalent compounds 5 and 6 and in accordance with other Ce^III^ siloxides like 3, Ce{OSi(O*t*Bu)_3]3_(thf)_3_ (2.243(2)–2.249(2) Å), [Ce{OSi(O*t*Bu)_3_}_3_]_2_ (Ce–O_term_ 2.186(3)–2.202(3) Å),^26^ Ce{OSiPh_3_}_3_(thf)_3_ (Ce–O_avg_ 2.222(4) Å)^35^ and [Ce{OSiPh_3_}_3_]_2_ (Ce–O_term_ 2.141(7)–2.184(6) Å).^27^ As expected for semiquinolato ligands the six-membered rings display two shortened C–C and four elongated C–C bonds. Additionally, the six-membered rings are slightly bent in comparison to the flat aromatic hydroquinolato linkers in complexes 4, 5 and 6 resulting in an angle of 170.34° for 8 and 170.29° for 9, respectively (see Fig. 5, bottom). Notwithstanding, the bridging radicals engage in significant π-stacking as indicated by close semiquinolato–semiquinolato distances of 3.112 Å for 8 and 3.156 Å for 9. Overall, complexes 8 and 9 display the same arrangement of the semiquinolato radical bridges as observed in complexes [LnCl_2_(THF)_3_(μ-Me_4_sq)_2_]_2_ (Ln = Y, Gd) (Ct⋯Ct 3.097 Å; Ct = centroid of benzene rings).^13^

**Fig. 6:**
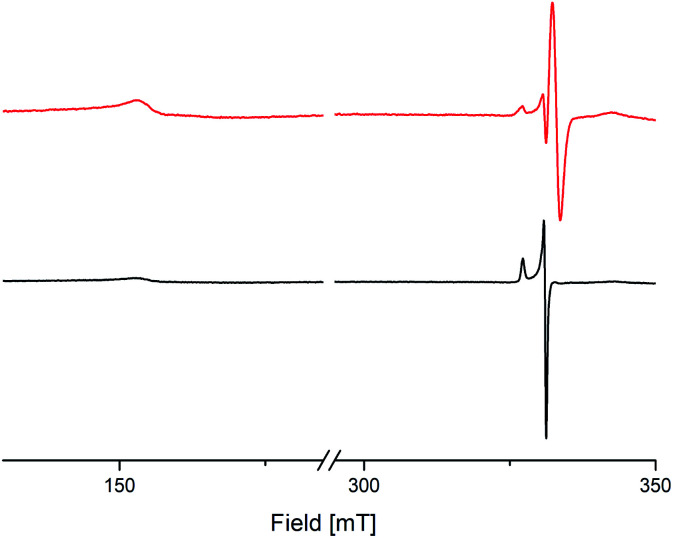
X-Band cw-EPR spectra of crystalline [Ce{OSi(O*t*Bu)_3_}_2_(thf)_2_(μ_2_-O_2_C_6_Me_4_)]_2_ (8) (black trace) and [Ce(OSi^i^Pr_3_)_2_(thf)_2_)(μ_2_-O_2_C_6_Me_4_)]_2_ (9) (red trace).

To investigate the electronic behaviour of the bridging semiquinolates, X-band EPR spectra of compounds 8 and 9 were recorded from a crystal powder sample at 123 K (Fig. 6). For both complexes cw-EPR spectra are composed of two distinct sets of resonances, one that results from the transition within the Kramers doublet corresponding to *m*_j_ = ±1/2 and one at half-field, *H* ≈ 160 mT. The transition for the *m*_j_ = ±1/2 state associates with an axial *g* tensor with principal components *g*_II_ = 2.094 and g_⊥_ = 2.032 for 8 and *g*_II_ = 2.088 and g_⊥_ = 2.032 for 9, respectively. The transition locating at half-field gives rise to a very broad dispersion line with *g*_II_ ≈ 4.359 for 8 and *g*_II_ ≈ 4.351 for 9, respectively. Notably, an identical line pattern derives from a frozen 2-Me-THF solution at 77 K; *cf.* Fig. S67, ESI,† for pertinent details. The cw-EPR spectra corroborate the presence of Ce^3+^, and agree with early work on mononuclear complexes of Ce^3+^.^36^ This indicates a radical–radical π-bonding, as it was recently shown for yttrium and gadolinium semiquinolates [LnCl_2_(thf)_2_]_2_(μ_2_-O_2_C_6_Me_4_)_2_ (Ln = Y or Gd).^13^ Additionally and also similar to the yttrium semiquinolate, complex 9 shows a signal with a *g* value of 1.999, which most likely results from a non-coupled radical impurity.

Note that the reactions of cerium siloxides 2 and 3 with 1,4-naphthoquinone resulted also in the formation of homoleptic ceric siloxides as well as paramagnetic by-products (*cf.* Fig. S36, ESI†) indicating a similar reactivity as observed for Me_4_BQ. Unfortunately, any putative semiquinolate complexes could not be isolated.

## Conclusions

Cerium(iii) silylamides and siloxides are suitable reagents for assessing the oxidising power/reducibility of differently substituted 1,4-quinones in non-aqueous solutions. The cerium–quinone redox matching is revealed by the ease of formation of Ce^IV^ hydroquinolates [CeL_3_]_2_(μ_2_-O_2_C_6_R_4_), in the case of the parent 1,4-benzoquinone (BQ) or when R represents electron-withdrawing groups (Cl, CN). Depending on their reduction potential, alkyl-substituted BQs engage in redox equilibria, with the Ce^IV^ hydroquinolate species being preferentially stable in the solid state, but also afford semiquinolates *via* redox ligand redistribution. The structurally characterized siloxide semiquinolate complexes [(CeL_2_(thf)_2_)(μ_2_-O_2_C_6_Me_4_)]_2_ (L = OSi(O*t*Bu)_3_, OSi^i^Pr_3_) exhibit a molecular arrangement, recently detected for [LnCl_2_(THF)_3_(μ-Me_4_sq)_2_]_2_ (Ln = Y, Gd).^13^ The stabilisation of the tetravalent oxidation state in hydroquinolato-bridged complexes [Ce^IV^L_3_]_2_(μ_2_-O_2_C_6_R_4_) was examined by electrochemical measurements, as well as NMR and UV/Vis spectroscopies. In accordance with previous findings,^16,18–20^ the stability of the ceric complexes increases in the order of N(SiMe_3_)_2_ < OSi(O*t*Bu_3_)_3_ < OSi^i^Pr_3_ as supporting ligand, but surprisingly drops in reverse order of the oxidation potential of the 1,4-quinones, being the least stable for the 2,3-dichloro-5,6-dicyano-hydroquinolato congener. The preferred formation of hydroquinolato-bridged silylamides [Ce{N(SiMe_3_)_2_}_3_]_2_(μ_2_-O_2_C_6_R_4_) seems kinetically favoured. Finally, the electrochemical reduction of the hydroquinolato-bridged ceric complexes [Ce^IV^L_3_]_2_(μ_2_-O_2_C_6_R_4_) can be mimicked by chemical reduction with cobaltocene, as shown for the isolation and structural characterisation of cerous [(Ce{N(SiMe_3_)_2_}_3_)_2_(μ_2_-O_2_C_6_H_4_)][CoCp_2_]_2_. The cerium–quinone redox matching and tuning might be used as a role-model in tetravalent praseodymium and terbium chemistry.

## Conflicts of interest

There are no conflicts to declare.

## Supplementary Material

SC-012-D0SC04489J-s001

SC-012-D0SC04489J-s002
