# Supplementary material for: Cerium–quinone redox couples put under scrutiny
Source: Chem Sci. 2020 Nov 23;12(4):1343–51. doi: 10.1039/d0sc04489j (PMC8179043; doi:10.1039/d0sc04489j)
Supplement: SC-012-D0SC04489J-s001 [file SC-012-D0SC04489J-s001.pdf]

Electronic Supplementary Material (ESI) for Chemical Science.
This journal is © The Royal Society of Chemistry 2020

Supporting Information

The cerium–quinone redox couples put under scrutiny

Uwe Bayer, Daniel Werner, Andreas Berkefeld, Cäcilia Maichle-Mössmer and Reiner Anwander*

Table of Contents

Experimental Section - Procedures **S3**

NMR Spectra **S11**

IR Spectra **S32**

Cyclic voltammetry measurements **S38**

UV/Vis spectra **S49**

EPR spectra **S51**

Crystallographic Data **S53**

References **S63**

Experimental Section

General Procedures. All manipulations were performed under an inert atmosphere (Ar) using a glovebox (MBraun 200B; <0.1 ppm O_2, <0.1 ppm H_2O), or according to standard Schlenk techniques in oven-dried glassware. The solvents were purified with Grubbs-type columns (MBraun SPS, solvent purification system) and stored in a glovebox. Anhydrous cerium(III) chloride (99.9%) was purchased from Sigma Aldrich and activated by Soxhlet extraction with THF giving $CeCl_3(thf)_{1.04}$. Potassium bis(trimethylsilyl)amide was purchased from Sigma Aldrich and purified by high-vacuum sublimation before use. 1,4-Benzoquinone was purchased from Sigma Aldrich and purified by sublimation. Tris-*tert*-butoxy silonol, tri*iso*propyl silanol, tetramethyl-1,4-benzoquinone, 1,4-naphthoquinone and 9,10-anthraquinone were purchased from Sigma Aldrich, tetrachloro-1,4-benzoquinone and 2,5-di-*tert*-butyl-1,4-benzoquinone from TCI Deutschland GmbH and used as received. C_6D_6, toluene-d_8 and THF-d_8 were purchased from Sigma Aldrich, degassed, dried by being stirred over NaK alloy for 24 h, filtered and stored in a glovebox. $Ce[N(SiMe_3)_2]_3$ (**1**), $[Ce\{OSi(OtBu)_3\}_3]_2$ (**2**), $[Ce\{N(SiMe_3)_2\}_3]_2(\mu_2\text{-}O_2C_6H_4)$ (**4^hq^**), $Ce_4(Me_2pz)_{12}$·¼ *n*-hexane and $[nPr_4N][B(C_6H_3(CF_3)_2\text{-}3,5)_4]$ were synthesised according to literature procedures.[1–5] NMR spectra were recorded on a Bruker AVII+400 (^{1}H: 400.13 MHz, ^{13}C: 100.61 MHz), a Bruker AVIIIHD-300 (^{1}H: 300.13 MHz, ^{13}C: 75.47 MHz, ^{29}Si: 59.63 MHz) or a Bruker AVII+ 500 (^{1}H: 500.13 MHz, ^{13}C: 125.76 MHz, ^{29}Si: 99.36 MHz) at 26 °C. NMR shifts are referenced to a solvent resonance and reported in parts per million (ppm) relative to tetramethylsilane.[6] Analysis of the NMR spectra was performed with ACD/NMR Processor Academic Edition (Product Version: 12.01). Multiplicities of signals are given as s (singlet), bs (broad singlet), d (doublet) and dd (doublet of doublets), sept (septet). Coupling constants (J) are given in Hz. Infrared spectra were recorded on a ThermoFisher Scientific NICOLET 6700 FTIR ($\tilde{v}$ = 4000 – 400 cm^{-1}) spectrometer using a DRIFT chamber with dry KBr/sample mixtures and KBr windows. Recorded spectra were converted using Kubelka-Munk correction. Elemental analysis (C/H/N) was performed on an Elementar vario MICRO cube. UV/Vis measurements were carried out in toluene on a PG Instruments T60 UV-Vis spectrophotometer. The effective magnetic moments were determined according to Evans' Method on a Bruker AVII+400 at 25 °C (^{1}H: 400.13 MHz), using C_6D_6 as a solvent and hexamethyldisiloxane as reference.[7] Cyclic voltammetry measurements were conducted in a glovebox under argon atmosphere using a Nordic Electrochemistry ECi-200 workstation in IR-compensation mode. Data recording was done using Nordic Electrochemistry EC4 DAQ (version 4.1.90.1) and processed with Nordic Electrochemistry EC-4 VIEW (version 1.2.36.1). The experiments were performed in a 4 mL glass vial with a CHI 104 glassy carbon disc working electrode, a Ag/AgCl quasi reference electrode and a platinum wire counter electrode using c(analyte) = 2 mM and c($[nPr_4N][B(C_6H_3(CF_3)_2\text{-}3,5)_4]$) = 0.1 M in THF. The reported potentials are given in [V] and are referenced vs. the Fc/Fc$^+$ couple which was added as an internal standard at the end of each measurement. EPR spectra were measured on a continuous wave X-Band Bruker ESP 300E using 4 mm O.D. Wilmad quartz (CFQ) EPR tubes. Spectra were referenced to the Bruker strong pitched standard giso= 2.0088.

Synthesis of [Ce(OSi*i*Pr$_3$)$_3$]$_2$ (3). *i*Pr$_3$SiOH (1.15 g, 6.60 mmol) was dissolved in *n*-hexane (6 mL) and added to a solution of Ce[N(SiMe$_3$)$_2$]$_3$ (1.37 g, 2.20 mmol) in *n*-hexane (6 mL). The solution was stirred for 16 h, then concentrated in vacuo and stored at −40 °C. After 16 h the supernatant solution was removed, and the resulting colourless crystals were dried in vacuum giving **3**. Yield: 1.01 g (70%). ^{1}H NMR (C_6D_6, 400.13 MHz, 26 °C): δ = 9.09 (bs, 12 H, terminal C*H*(CH$_3$)$_2$), 6.46 (bs, 72 H, terminal CH(C*H*$_3$)$_2$), −17.23 (bs, 36 H, μ_2-C*H*(CH$_3$)$_2$), −28.82 (bs, 6H, μ_2-CH(C*H*$_3$)$_2$) ppm; ^{1}H NMR (THF-d$_8$, 400.13 MHz, 26 °C): δ =2.98 (bs, 9 H, C*H*(CH$_3$)$_2$), 1.58 (bs, 54 H, CH(C*H*$_3$)$_2$) ppm; IR (DRIFT): $\tilde{v}$ = 2935 (vs), 2861 (vs), 2745 (vw), 2715 (vw), 1462 (s), 1478 (w), 1292 (vw), 1240 (w), 1157 (vw), 1051 (w), 988 (m), 911 (s), 882 (s), 841 (m), 666(s), 580 (vw), 514 (w), 464 (m), 401 (m) cm^{-1}; elemental analysis (%) calcd. for $C_{54}H_{126}Ce_2O_6Si_6$ (1320.34): C 49.12, H 9.62; found: C 48.99, H 9.56.

Synthesis of [Ce{N(SiMe$_3$)$_2$}$_3$]$_2$(μ_2-O$_2$C$_6$H$_4$) (4^{hq}). 1,4-Benzoquinone (0.0191 g, 0.177 mmol) was dissolved in toluene (1 mL) and added to a solution of Ce[N(SiMe$_3$)$_2$]$_3$ (0.220 g, 0.354 mmol) in toluene (3 mL). After being stirred for 2 h the solution was concentrated in vacuo (1-2 mL) and stored at −40 °C. After 2 days the supernatant solution was removed. The brown solid was dried in vacuum giving microcrystalline **4^{hq}**. Yield: 0.159 g (69%). ^{1}H NMR (C_6D_6, 400.13 MHz): δ = 7.28 (s, 4 H, μ_2-O$_2$C$_6$H$_4$), 0.42 (s, 108 H, SiMe$_3$) ppm; UV/Vis: 485 nm (14964 ± 3215 L mol^{-1} cm^{-1}); elemental analysis (%) calcd. for $C_{42}H_{112}Ce_2N_6O_2Si_{12}$ (1350.64): C 37.34, H 8.36, N 6.22; found: C 37.01, H 8.37, N 6.14; μ_{eff} = 0.67 BM (1.06·10^{-5} mol L^{-1}, Δ = 2.4 Hz). The ^{1}H NMR spectrum was in accordance with literature.[3]

Synthesis of [Ce{N(SiMe$_3$)$_2$}$_3$]$_2$(μ_2-O$_2$C$_6$Cl$_4$) (4^{Cl4hq}). Tetrachloro-1,4-benzoquinone (0.0601 g, 0.242 mmol) was dissolved in toluene (3 mL) and added to a solution of Ce[N(SiMe$_3$)$_2$]$_3$ (0.300 g, 0.483 mmol) in *n*-hexane (2 mL). After being stirred for 16 h the solution was stored at −40 °C and after 4 d the supernatant solution was removed. The remaining purple/brown solid was dried in vacuo producing **4^{Cl4hq}** as a dark purple solid. Yield: 0.279 g (77%). ^{1}H NMR (C_6D_6, 400.13 MHz): δ = 0.45 (s, 108 H, SiMe$_3$) ppm; ^{29}Si DEPT45 NMR (C_6D_6, 59.63 MHz, 26 °C): δ = −8.1 ppm; due to low solubility, a ^{13}C NMR spectrum could not be obtained; IR (DRIFT): $\tilde{v}$ = 2952 (m), 2899 (w), 1414 (vs), 1376 (w), 1251 (s), 1211 (w), 1174 (w), 934 (s), 896 (vs), 873 (s), 846 (vs), 771 (s), 731 (w), 718 (w), 675 (m), 656 (s), 607 (s), 502 (m) cm^{-1}; UV/Vis: 319 nm (ε = 11602 ± 1308 L mol^{-1} cm^{-1}), 518 nm (ε = 13180 ± 1580 L mol^{-1} cm^{-1}); elemental analysis (%) calcd for $C_{42}H_{108}Ce_2Cl_4N_6O_2Si_{12}$ (1488.42): C 33.89, H 7.31, N 5.65; found: C 34.09, H 7.17, N 5.59; μ_{eff} = 0.68 BM (1.08·10^{-5} mol L^{-1}, Δ = 2.5 Hz).

Synthesis of [Ce{N(SiMe$_3$)$_2$}$_3$]$_2$(μ_2-O$_2$C$_6$Cl$_2$(CN)$_2$) (4^{ddhq}). 2,3-Dichloro-5,6-dicyano-1,4-benzoquinone (0.0273 g, 0.121 mmol) was dissolved in toluene (3 mL) and added to a solution of Ce[N(SiMe$_3$)$_2$]$_3$ (0.150 g, 0.242 mmol) in *n*-hexane (2 mL). After being stirred for 16 h the solution was stored at −40 °C and after 1 d the supernatant solution was removed. The remaining purple/brown solid was dried in vacuo producing **4^{ddhq}** as a dark red solid. Yield: 0.145 g (81%). ^{1}H NMR (C_6D_6, 400.13 MHz): δ = 0.45 (s, 108 H, SiMe$_3$) ppm; ^{13}C{^{1}H} NMR (C_6D_6, 125.61 MHz,

26 °C): δ = 161.1 (2 C, C_{arom}–O), 132.2 (2 C, C_{arom}–Cl), 115.7 (2 C, C_{arom}–*C*N), 103.6 (2 C, C_{arom}–CN), 5.6 (36 C, $SiMe_3$) ppm; ^{29}Si DEPT45 NMR (C_6D_6, 59.63 MHz, 26 °C): δ = −7.3 ppm; IR (DRIFT): $\tilde{v}$ = 2953 (w), 2900 (vw), 2227 (vw), 1406 (s), 1251 (s), 1093 (vw), 1003 (m), 888 (vs), 844 (vs), 771 (m), 654 (m), 607 (s), 547 (w) cm^{-1}; UV/Vis: 384 nm (ε = 4510 ± 117 L mol^{-1} cm^{-1}), 511 nm (ε = 5060 ± 202 L mol^{-1} cm^{-1}); elemental analysis (%) calcd for $C_{44}H_{108}Ce_2Cl_2N_8O_2Si_{12}$ (1469.55): C 35.96, H 7.41, N 7.63; found: C 36.17, H 7.07, N 7.54; μ_{eff} = 0.59 BM (6.60·10^{-6}mol L^{-1}, Δ = 1.0 Hz).

Synthesis of [Ce{N(SiMe₃)₂}₃]₂(*μ*₂-O₂C₆Me₄) (4^Me4hq). Tetramethyl-1,4-benzoquinone (0.0264 g, 0.161 mmol) was dissolved in *n*-hexane (2 mL) and added to a solution of $Ce[N(SiMe_3)_2]_3$ (0.200 g, 0.322 mmol) in *n*-hexane (2 mL). After being stirred for 5 min the solution was evaporated to dryness giving **4^{Me4hq}** as a dark brown solid. Yield: 0.203 g (90%). ^{1}H NMR (toluene-*d*$_8$, 500.13 MHz, 273 K): δ =2.67 (s, 12 H, μ_2-$O_2C_6Me_4$), 0.43 (s, 108 H, $SiMe_3$) ppm; ^{29}Si INEPTND NMR (C_6D_6, 59.63 MHz, 26 °C): δ = −8.8 ppm; due to low solubility and fast decomposition, a ^{13}C NMR spectrum could not be obtained; IR (DRIFT): $\tilde{v}$ =2952 (m), 2899 (w), 1450 (w), 1399 (w), 1385 (w), 1370 (vw), 1255 (vs), 1079 (s), 984 (w), 918 (s), 903 (s), 863 (vs), 837 (s), 773 (s), 732 (w), 675 (s), 658 (vs), 609 (s), 416 (m) cm^{-1}; UV/Vis: 362 nm (ε = 1249 ± 44 L mol^{-1} cm^{-1}), 411 nm (ε = 1610 ± 49 L mol^{-1} cm^{-1}), 681 nm (ε = 556 ± 24 L mol^{-1} cm^{-1}); elemental analysis (%) calcd. for $C_{46}H_{120}Ce_2N_6O_2Si_{12}$ (1406.76): C 39.28, H 8.60, N 5.97; found: C 39.29, H 8.81, N 5.92; μ_{eff} = 0.89 BM (1.07·10^{-5} mol L^{-1}, Δ = 5.1 Hz).

Synthesis of $[Ce\{N(SiMe_3)_2\}_3]_2(\mu_2\text{-}O_2C_6tBu_2H_2)$ (4^{tBu2hq}). $Ce[N(SiMe_3)_2]_3$ (0.300 g, 0.483 mmol) was dissolved in *n*-hexane (3 mL) and added to a suspension of 2,5-di-*tert*-butyl-1,4-benzoquinone (0.0532 g, 0.241 mmol) in *n*-hexane (2 mL). After being stirred for 2 h the solution was stored at −40 °C and after 1 d the supernatant solution was removed. The remaining brown solid was dried in vacuo giving **4^{tBu2hq}** as the crude product. Yield: 0.308 g (88%).^{1}H NMR (C_6D_6, 400.13 MHz): δ = 0.45 (s, 108 H, $SiMe_3$) ppm; due to the redox equilibrium between the species formed in the solid state and solution and the resulting low concentration of **4^{tBu2hq}** in solution, no other peaks corresponding to the product could be observed; IR (DRIFT): $\tilde{v}$ = 3019 (w), 2965 (m), 2952 (m), 2900 (w), 1484 (w), 1392 (w), 1355 (w), 1251 (vs), 1198 (w), 1186 (m), 1118 (w), 893 (s), 860 (vs), 837 (vs), 831 (vs), 773 (s), 673 (m), 658 (s), 634 (w), 611 (m), 463 (m), 448 (w) cm^{-1}; elemental analysis (%) calcd. for $C_{50}H_{128}Ce_2N_6O_2Si_{12}$ (1467.87): C 41.05, H 8.82, N 5.74; found: C 41.05, H 8.87, N 5.74.

Synthesis of $[Ce\{N(SiMe_3)_2\}_3]_2(\mu_2\text{-}O_2C_{10}H_6)$ (4^{nhq}). 1,4-Naphthoquinone (0.0127 g, 0.0805 mmol) was dissolved in *n*-hexane (1 mL) and added to a solution of $Ce[N(SiMe_3)_2]_3$ (0.100 g, 0.161 mmol) in *n*-hexane (1 mL). After being stirred for 30 min the solution was stored at −40 °C. After 10 d small reddish brown crystals were handpicked and analysed by X-ray crystallography. The supernatant solution was removed and the remaining solid was dried in vacuo giving **4^{nhq}** as a brown solid. Yield: 0.080 g (71%). ^{1}H NMR (C_6D_6, 400.13 MHz): δ = 8.57 (dd, 2 H, $^3J_{HH}$ = 6.46 Hz, $^4J_{HH}$ = 3.30 Hz, H-8, H-9), 7.52 (dd, 2 H, $^3J_{HH}$ = 6.34 Hz, $^4J_{HH}$ = 3.30 Hz, H-7, H-10),

7.24 (s, 2 H, H-2, H-3), 0.44 (s, 108 H, $SiMe_3$) ppm; $^{13}C\{^1H\}$ NMR (C_6D_6, 100.61 MHz, 26 °C): δ = 165.8 (2 C, C_{arom}–O), 125.8 (2 C, C_{arom}), 125.0 (2 C, C_{arom}), 120.7 (2 C, C_{arom}), 114.3 (2 C, C_{arom}), 5.6 (36 C, $SiMe_3$) ppm; ^{29}Si DEPT45 NMR (C_6D_6, 59.63 MHz, 26 °C): δ = −8.1 ppm; IR (DRIFT): $\tilde{v}$ = 3068 (w), 3041 (w), 2951 (m), 2897 (w), 1574 (w), 1449 (m), 1377 (s), 1266 (vs), 1250 (vs), 1227 (m), 1215 (m), 1151 (w), 1077 (m), 1053 (w), 1014 (w), 905 (vs), 881 (s), 841 (vs), 826 (s), 776 (vs), 758 (s), 735 (m), 674 (m), 657 (s), 611 (s), 501 (w) cm^{-1}; UV/Vis: 339 nm (ε = 8386 ± 1267 L mol^{-1} cm^{-1}), 425 nm (ε = 3956 ± 576 L mol^{-1} cm^{-1}), 474 nm (ε = 4405 ± 646 L mol^{-1} cm^{-1}), 678 nm (ε = 2391 ± 362 L mol^{-1} cm^{-1}); elemental analysis (%) calcd. for $C_{46}H_{114}Ce_2N_6O_2Si_{12}$ (1400.71): C 39.44, H 8.20, N 6.00; found: C 40.54, H 7.74, N 5.93; due to rapid decomposition no better microanalytical data could be obtained; μ_{eff} = 1.19 BM ($1.08 \cdot 10^{-5}$ mol L^{-1}, Δ = 9.76 Hz).

Synthesis of [Ce{OSi(O*t*Bu)$_3$}$_3$(thf)]$_2$(μ_2-$O_2C_6H_4$) (5^{hq}). 1,4-Benzoquinone (0.0116 g, 0.107 mmol) was dissolved in THF (2 mL) and added to a solution of [Ce{OSi(O*t*Bu)$_3$}$_3$]$_2$ (0.200 g, 0.215 mmol) in THF (2 mL). After being stirred for 1 h the solution was evaporated to dryness, re-dissolved in *n*-hexane (1.5 mL) and stored at −40 °C. After 8 d the supernatant solution was removed and the remaining solid was dried in vacuo giving **5^{hq}** as a dark purple solid. Yield: 0.107 g (46%). ^{1}H NMR (THF-d_8, 400.13 MHz, 26 °C): δ = 6.54 (s, 4 H, μ_2-$O_2C_6H_4$), 3.62 (m, 8 H, β-THF), 1.78 (m, 8 H, α-THF), 1.36 (s, 162 H, O*t*Bu) ppm; $^{13}C\{^1H\}$ NMR (THF-d_8, 100.61 MHz, 26 °C): δ = 166.9 (2 C, C_{arom}–O), 119.3 (4 C, C_{arom}–H), 72.5 (18 C, $C(CH_3)_3$), 32.6 (54 C, $C(CH_3)_3$) ppm; ^{29}Si NMR (from ^{1}H-^{29}Si HSQC, THF-d_8, 99.36 MHz, 26 °C): δ = –103.2 ppm; IR (DRIFT): $\tilde{v}$ = 2972 (vs), 2930 (m), 2873 (w), 1490 (s), 1473 (w), 1387 (m), 1364 (s), 1242 (vs), 1193 (vs), 1056 (vs), 1026 (vs), 986 (s), 924 (vs), 913 (vs), 836 (s), 801 (w), 698 (s), 626 (w), 539 (m), 513 (m), 499 (m), 492 (m), 455 (m), 429 (m) cm^{-1}; UV/Vis: 622 nm (ε = 4831 ± 224 L mol^{-1} cm^{-1}), 369 nm (ε = 3832 ± 286 L mol^{-1} cm^{-1}); elemental analysis (%) calcd. for $C_{86}H_{182}Ce_2O_{28}Si_6$ (2113.12): C 48.88, H 8.86; found: C 48.82, H 8.42; μ_{eff} = 0.82 BM ($6.90 \cdot 10^{-7}$ mol L^{-1}, Δ = 2.66 Hz).

Synthesis of [Ce{OSi(O*t*Bu)$_3$}$_3$(thf)]$_2$(μ_2-$O_2C_6Cl_4$) (5^{Cl4hq}). Tetrachloro-1,4-benzoquinone (0.0264 g, 0.107 mmol) was dissolved in THF (2 mL) and added to a solution of [Ce{OSi(O*t*Bu)$_3$}$_3$]$_2$ (0.200 g, 0.215 mmol) in THF (4 mL). After being stirred for 1 h the solution was stored at −40 °C. After 3 d the supernatant solution was removed and the remaining solid was dried in vacuo giving **5^{Cl4hq}** as a red solid. Yield: 0.0960 g (42%). ^{1}H NMR (THF-d_8, 400.13 MHz, 26 °C): δ = 3.62 (m, 8 H, β-THF), 1.78 (m, 8 H, α-THF), 1.36 (s, 162 H, O*t*Bu) ppm; $^{13}C\{^1H\}$ NMR (THF-d_8, 100.61 MHz, 26 °C): δ = 154.8 (2 C, C_{arom}–O), 121.3 (4 C, C_{arom}–Cl), 72.8 (18 C, $C(CH_3)_3$), 32.4 (54 C, $C(CH_3)_3$) ppm; ^{29}Si DEPT45 NMR (THF-d_8, 59.63 MHz, 26 °C): δ = −104.6 ppm; IR (DRIFT): $\tilde{v}$ = 2974 (vs), 2930 (m), 2902 (w), 2872 (w), 1471 (w), 1421 (s), 1388 (s), 1364 (s), 1242 (s), 1192 (vs), 1062 (s), 1053 (s), 1027 (s), 996 (m), 911 (s), 886 (s), 829 (m), 716 (w), 701 (m), 644 (vw), 513 (w), 495 (m), 442 (m), 431 (w) cm^{-1}; UV/Vis: 493 nm (ε = 3787 ± 433 L mol^{-1} cm^{-1}); elemental analysis (%) calcd. for $C_{86}H_{178}Ce_2Cl_4O_{28}Si_6$ (2250.88): C 45.89, H 7.97; found: C: 45.55, H 7.92; μ_{eff} = 0.54 BM ($6.46 \cdot 10^{-7}$ mol L^{-1}, Δ = 0.8 Hz).

Synthesis of [Ce{OSi(O*t*Bu)$_3$}$_3$(thf)]$_2$(μ_2-O$_2$C$_6$Cl$_2$(CN)$_2$) (5^{ddhq}). 2,3-Dichloro-5,6-dicyano-1,4-benzoquinone (0.0183 g, 0.0807 mmol) was dissolved in THF (2 mL) and added to a solution of [Ce{OSi(O*t*Bu)$_3$}$_3$]$_2$ (0.150 g, 0.161 mmol) in THF (4 mL). After being stirred for 1 h the solution was stored at −40 °C. After 3 d the supernatant solution was removed and the remaining solid was dried in vacuo giving **5^{ddhq}** as a red solid. Yield: 0.0833 g (46%). ^{1}H NMR (THF-d_8, 400.13 MHz, 26 °C): δ = 3.62 (m, 8 H, β-THF), 1.78 (m, 8 H, α-THF), 1.36 (s, 162 H, O*t*Bu) ppm; ^{13}C{^{1}H} NMR (THF-d_8, 100.61 MHz, 26 °C): δ = 160.8 (2 C, C$_{arom}$–O), 132.3 (2 C, C$_{arom}$–Cl), 115.1 (2 C, C$_{arom}$–*C*N), 99.3 (2 C, *C*$_{arom}$–CN), 73.0 (18 C, *C*(CH$_3$)$_3$), 32.5 (54 C, C(*C*H$_3$)$_3$) ppm; ^{29}Si DEPT45 NMR (THF-d_8, 59.63 MHz, 26 °C): δ = −105.3 ppm; IR (DRIFT): $\tilde{v}$ = 2974 (s), 2930 (w), 2872 (vw), 2227 (vw), 1472 (vw), 1457 (vw), 1417 (s), 1389 (m), 1364 (s), 1240 (m), 1213 (w), 1191 (s), 1058 (vs), 1026 (s), 976 (m), 887 (vs), 829 (w), 802 (vw), 701 (w), 630 (vw), 545 (w), 510 (vw), 492 (vw), 473 (vw), 444 (vw), 431 (vw) cm^{-1}; UV/Vis: 384 nm (ε = 2143 ± 375 L mol^{-1} cm^{-1}), 450 nm (ε = 2718 ± 326 L mol^{-1} cm^{-1}); elemental analysis (%) calcd. for $C_{88}H_{178}Ce_2Cl_2N_2O_{28}Si_6$ (2232.02): C 47.35, H 8.04, N 1.26; found: C: 47.40, H 7.74, N 1.40; μ_{eff} = 0.60 BM (4.59 10^{-6} mol L^{-1}, Δ = 0.72 Hz).

Synthesis of [Ce(OSi*i*Pr$_3$)$_3$(thf)]$_2$(μ_2-O$_2$C$_6$H$_4$) (6^{hq}). 1,4-Benzoquinone (0.0123 g, 0.114 mmol) was dissolved in THF (2 mL) and added to a solution of [Ce(OSi*i*Pr$_3$)$_3$]$_2$ (0.150 g, 0.114 mmol) in THF (2 mL). After being stirred for 1 h the solution was concentrated (to ~1 mL), *n*-hexane (1 mL) was added and stored at −40 °C. After 1 d the supernatant solution was removed and the remaining solid was dried in vacuo giving **6^{hq}** as a dark purple solid. The remaining crystals were dried in vacuo giving **6^{hq}** as a purple solid. Yield: 0.128 g (71%). ^{1}H NMR (THF-d_8, 400.13 MHz): δ = 6.01 (s, 4 H, μ_2-O$_2$C$_6$H$_4$), 1.14 (d, 108 H, CH(C*H*$_3$)$_2$, 1.10 (sept, 18 H, C*H*(CH$_3$) ppm; ^{29}Si DEPT45 NMR (THF-d_8, 59.63 MHz, 26 °C): δ = 7.0 ppm; due to low solubility, a ^{13}C NMR spectrum could not be obtained; IR (DRIFT): $\tilde{v}$ = 2939 (vs), 2863 (vs), 1482 (s), 1381 (vw), 1228 (s), 1070 (w), 1035 (m), 990 (m), 936 (m), 882 (s), 862 (s), 829 (s), 678 (s), 594 (vw), 519 (vw), 402 (m) cm^{-1}; UV/Vis: 526 nm (ε = 1402 ± 88 L mol^{-1} cm^{-1}); elemental analysis (%) calcd. for $C_{68}H_{146}Ce_2O_{10}Si_6$ (1572.65): C 51.93, H 9.36; found: C 52.19, H 9.03; μ_{eff} = 0.68 BM (3.29 10^{-6} mol L^{-1}, Δ = 0.76 Hz).

Synthesis of [Ce(OSi*i*Pr$_3$)$_3$(thf)]$_2$(μ_2-O$_2$C$_6$Cl$_4$) (6^{Cl4hq}). Tetrachloro-1,4-benzoquinone (0.0279 g, 0.114 mmol) was dissolved in THF (2 mL) and added to a solution of [Ce(OSi*i*Pr$_3$)$_3$]$_2$ (0.150 g, 0.114 mmol) in THF (4 mL). After being stirred for 1 h the solution was stored at −40 °C. After 3 d the supernatant solution was removed and the remaining solid was dried in vacuo giving **6^{Cl4hq}** as a red solid. Yield: 0.110 g (56%). ^{1}H NMR (THF-d_8, 400.13 MHz): δ = 1.12 (d, $^3J_{HH}$ = 6.04 Hz, 108 H, CH(C*H*$_3$)$_2$, 1.05 (sept, $^3J_{HH}$ = 5.87 Hz,18 H, C*H*(CH$_3$) ppm; ^{13}C{^{1}H} NMR (THF-d_8, 100.61 MHz, 26 °C): δ = 153.5 (2 C, C$_{arom}$–O), 120.3 (4 C, C$_{arom}$–Cl), 18.0 (36 C, *C*H(CH$_3$)$_2$), 14.0 (18 C, CH(*C*H$_3$)$_2$) ppm; ^{29}Si DEPT45 NMR (THF-d_8, 59.63 MHz, 26 °C): δ = 9.6 ppm; IR (DRIFT): $\tilde{v}$ = 2940 (vs), 2863 (vs), 1462 (m), 1421 (s), 1379 (w), 1241 (vw), 1205 (vw), 1176 (vw), 1030 (w), 992 (w), 956 (w), 905 (vs), 883 (vs), 833 (vs), 714 (m), 676 (s), 493 (m), 451

(vw), 413 (w) cm^{-1}; UV/Vis: 511 nm (ε = 3203 ± 287 L mol^{-1} cm^{-1}); elemental analysis (%) calcd. for $C_{68}H_{142}Ce_2Cl_4O_{10}Si_6$ (1720.42): C 47.75, H 8.37; found: C: 47.71, H 8.34; μ_{eff} = 0.50 BM (6.14 10^{-6} mol L^{-1}, Δ = 0.51 Hz).

Synthesis of [Ce(OSi*i*Pr$_3$)$_3$(thf)]$_2$(μ_2-O$_2$C$_6$Cl$_2$(CN)$_2$) (6^{ddhq}). 2,3-Dichloro-5,6-dicyano-1,4-benzoquinone (0.0258 g, 0.114 mmol) was dissolved in THF (2 mL) and added to a solution of [Ce(OSi*i*Pr$_3$)$_3$]$_2$ (0.150 g, 0.114 mmol) in THF (4 mL). After being stirred for 1 h the solution was stored at −40 °C. After 3 d the supernatant solution was removed and the remaining solid was dried in vacuo giving **6^{ddhq}** as a red solid. Yield: 0.107 g (55%). ^{1}H NMR (THF-d_8, 400.13 MHz): δ = 1.12 (d, $^{3}J_{HH}$ = 6.94 Hz, 108 H, CH(C*H*$_3$)$_2$, 1.05 (sept, $^{3}J_{HH}$ = 6.62 Hz,18 H, C*H*(CH$_3$) ppm; ^{13}C{^{1}H} NMR (THF-d_8, 100.61 MHz, 26 °C): δ = 161.1 (2 C, C$_{arom}$–O), 131.2 (2 C, C$_{arom}$–Cl), 117.7 (2 C, C$_{arom}$–*C*N), 99.1 (2 C, *C*$_{arom}$–CN), 19.3 (12 C, *C*H(CH$_3$)$_2$), 19.1 (24 C, *C*H(CH$_3$)$_2$), 15.5 (6 C, CH(*C*H$_3$)$_2$), 15.1 (12 C, CH(*C*H$_3$)$_2$) ppm; ^{29}Si INEPTND NMR (THF-d_8, 59.63 MHz, 26 °C): δ = 10.7 ppm; IR (DRIFT): $\tilde{v}$ = 2941 (vs), 2863 (vs), 2222 (w), 1461 (m), 1415 (s), 1228 (w), 1085 (vw), 1025 (w), 990 (w), 939 (m), 883 (s), 828 (vs), 679 (s), 599 (vw), 540 (w), 414 (vw) cm^{-1}; UV/Vis: 470 nm (ε = 4298 ± 125 L mol^{-1} cm^{-1}), 381 nm (ε = 10725 ± 214 L mol^{-1} cm^{-1}); elemental analysis (%) calcd. for $C_{70}H_{142}Ce_2Cl_2N_2O_{10}Si_6$ (1691.55): C 49.70, H 8.46, N 1.66; found: C: 49.83, H 8.31, N 1.86; μ_{eff} = 0.66 BM (6.93 10^{-6} mol L^{-1}, Δ = 1.52 Hz)).

Synthesis of [Ce(OSi*i*Pr$_3$)$_3$(thf)]$_2$(μ_2-O$_2$C$_6$*t*Bu$_2$H$_2$)] (6^{tBu2hq}). [Ce(OSi*i*Pr$_3$)$_3$]$_2$ (0.100 g, 0.0760 mmol) was dissolved in THF (3 mL) and added to a suspension of 2,5-di-*tert*-butyl-1,4-benzoquinone (0.0167 g, 0.0760 mmol) in THF (2 mL). After being stirred for 2 h the solution was stored at −40 °C and after 2 d the supernatant solution was removed. The remaining dark purple solid was dried in vacuo giving **6^{tBu2hq}** as the crude product. Yield: 0.0543 g (42%). ^{1}H NMR (THF-d_8, 500.13 MHz, 233 K): δ = 6.51 (s, 2 H, μ_2-O$_2$C$_6$*t*Bu$_2$*H*$_2$), 1.25 (s, 18 H, μ_2-O$_2$C$_6$*tBu*$_2$H$_2$), 1.11 (d, 108 H, CH(C*H*$_3$)$_2$, 1.02 (sept, 18 H, C*H*(CH$_3$) ppm; IR (DRIFT): $\tilde{v}$ = 2958 (m), 2940 (m), 2887 (m), 2862 (m), 1487 (w), 1462 (w), 1392 (vw), 1366 (w), 1240 (vw), 1208 (w), 1119 (vw), 1034 (vw), 991 (w), 947 (m), 916 (w), 876 (s), 842(vs), 677 (m), 592 (vw), 570 (vw), 519 (vw), 446 (w), 412 (w) cm^{-1}; elemental analysis (%) calcd. for $C_{76}H_{162}Ce_2O_{10}Si_6$ (1684.86): C 54.18, H 9.69, found: C 53.93, H 9.42.

Reduction of [Ce{N(SiMe$_3$)$_2$}$_3$]$_2$(μ_2-O$_2$C$_6$H$_4$) (4^{hq}) with cobaltocene (CoCp$_2$)

Compound **4^{hq}** (32.2 mg, 23.9 µmol) was dissolved in THF (2 mL) and CoCp$_2$ (9.0 mg, 47.8 µmol) in THF (2 mL) was added. The reaction mixture turned from dark brown to pale yellow. After being stirred for 10 min the solution was concentrated and then stored at −40 °C. After 3 d the supernatant was removed and [(Ce{N(SiMe$_3$)$_2$}$_3$)$_2$(μ_2-O$_2$C$_6$H$_4$)][CoCp$_2$]$_2$·THF (**7**) was obtained as light brown crystals. Yield: 0.028 g (67%). ^{1}H NMR (THF-d_8, 400.13 MHz): δ = 3.88 (s, 4 H, hq), 3.79 (s, 20 H, Cp), −1.11 (s, 108 H, SiMe$_3$) ppm; IR (DRIFT): $\tilde{v}$ = 3111 (vw), 2943 (m), 2889 (w), 1486 (s), 1416 (w), 1293 (vw), 1241 (s), 1066 (vw), 998 (vs), 867 (s), 830 (vs), 768 (m), 666 (m), 593 (w), 458 (vw) cm^{-1}; elemental analysis (%) calcd. for $C_{66}H_{140}Ce_2Co_2N_6O_3Si_{12}$ (1801.06): C 44.02, H 7.84, N 4.67; found: C: 45.08, H 7.53, N

4.32. EA calculated for one more molecule of THF in the crystal lattice: C 44.98, H 7.77, N 4.50; μ_{eff} = 3.26 B.M. (5.40 10^{-6} mol L^{-1}, Δ = 32.19 Hz).

Synthesis of [Ce{OSi(O*t*Bu)$_3$}$_2$(thf)$_2$(μ_2-$O_2C_6Me_4$)]$_2$ (8). Tetramethyl-1,4-benzoquinone (0.018 g, 0.11 mmol) was dissolved in THF (2 mL) and added to a solution of [Ce{OSi(O*t*Bu)$_3$}$_3$]$_2$ (0.200 g, 0.22 mmol) in THF (2 mL). After being stirred for 2 h the solution was stored at −40 °C. After 7 d dark turquoise crystals were handpicked and analysed by X-ray diffraction, indicating the formation of **8**. The remaining crystals were evaporated to dryness giving **8** together with [Ce{OSi(O*t*Bu)$_3$}$_4$], [Ce{OSi(O*t*Bu)$_3$}$_3$] and Me_4bq.

Synthesis of [Ce(OSi*i*Pr$_3$)$_2$(thf)$_2$)(μ_2-$O_2C_6Me_4$)]$_2$ (9). Tetramethyl-1,4-benzoquinone (0.018 g, 0.11 mmol) was dissolved in THF (2 mL) and added to a solution of [Ce(OSi*i*Pr$_3$)$_3$]$_2$ (0.200 g, 0.22 mmol) in THF (2 mL). After being stirred for 2 h the solution was stored at −40 °C. After 7 d dark turquoise crystals were handpicked and analysed by X-ray diffraction, indicating the formation of **9**. The remaining crystals were evaporated to dryness giving **9** together with [Ce(OSi*i*Pr$_3$)$_4$], [Ce(OSi*i*Pr$_3$)$_3$]$_2$ and Me_4bq.

Preliminary tests with pyrazolate complexes:

Reaction between $Ce_4(Me_2pz)_{12}$ and 9,10-anthraquinone, and isolation of [Ce$_3$(bpad)(pasq)(Me$_2$pz)$_6$(thf)] (bpad = 1,4-bis(3,5-dimethylpyrazol-1-yl)anthra-1,4-diolato; pasq = 1-(3,5-dimethylpyrazol-1-yl)anthra-1,4-semiqinolato). $Ce_4(Me_2pz)_{12}$·1/4 *n*-hexane·(0.0477 g, 0.028 mmol, trace toluene impurities not included) and 9,10-anthraquinone (0.0109 g, 0.0532 mmol) were combined in THF-d_8 (NMR-scale experiment, using a J. Young-valved NMR tube). No colour change was observed and the emergence of a broad peak in the NMR occurred. ^{1}H NMR (THF-d_8, 400.13 MHz, integration for anthracene is low due to poor solubility in THF-d_8, solvent peaks not included): δ = 11.92 (br s, 3 H, Me$_2$pz-CH), 8.27 (br s, μ_2-$O_2C_{14}H_8$), 6.38 (br s, 18 H, Me$_2$pz-CH$_3$) ppm. The solution was transferred to a vial, and the solvent removed under vacuum. Toluene was added and the light-yellow solution was filtered. Upon storage at −35 °C, crystals of **[Ce$_3$(bpad)(pasq)(Me$_2$pz)$_6$(thf)]** formed. The supernatant solution was removed (see below), and the crystals were dried in vacuo, giving an off-white powder of **[Ce$_3$(bpad)(pasq)(Me$_2$pz)$_6$(thf)]** (0.0527 g, 84%); IR (Nujol) $\tilde{v}$ = 1665 (m), 1597 (w), 1583 (w), 1552 (m), 1518 (vs), 1317 (vs), 1276 (s), 1243 (s), 1196 (w), 1176 (w), 1157 (w), 1066 (s), 1035 (vs), 1007 (s), 957 (vw), 930 (s), 890 (m), 855 (vw), 770 (s), 746 (m), 721 (vs), 695 (w), 683 (vw) cm^{-1}; Elemental analysis calcd. (%) for $C_{77}H_{87}Ce_3N_{18}O_5$ (1763.43): C 52.40, H 4.97, N 14.28; Found: C 51.96, H 3.98, N 14.09. The light-yellow supernatant solution was concentrated and THF and hexane was added. The colour of the solution turned to an emerald green. Upon storage at −35 °C crystals of semiquinolate **[Ce(Me$_2$pz)$_2$(thf)$_2$(asq)]$_2$** formed amongst colourless crystals.

Isolation of [Ce(*t*Bu$_2$pz)$_3$]$_2$(μ_2-$O_2C_6Me_4$). Ce[N(SiMe$_3$)$_2$]$_3$ (0.081 g, 0.13 mmol), and *t*Bu$_2$pzH (0.074 g, 0.41 mmol, in slight excess), were stirred in THF (5 mL) for 10 minutes. The solution was evaporated to dryness leaving a pale-yellow powder. Cyclohexane (4 mL) was added and

the powder dissolved giving a pale-yellow solution. Tetramethyl-1,4-benzoquinone (0.0070 g, 0.043 mmol) was added with stirring and the solution turned immediately dark green. The solution was reduced under vacuum and toluene was added (1 mL). The mixture slowly crystallised over two days. The supernatant solution was decanted, and the crystals were submerged in *n*-paratone crystallography oil. Examination under the microscope indicated a mixture of species, colourless needle crystals, large colourless block crystals and dark red/brown crystals. The dark red/brown block crystals were analysed as **$[Ce(tBu_2pz)_3]_2(\mu_2\text{-}O_2C_6Me_4)$**.

Stability tests of putative $[(Ce\{N(SiHMe_2)_2\}_3)_2(\mu_2\text{-}O_2C_6R_4)]$ (R = H, Me) species:

Reaction between $[Ce\{N(SiHMe_2)_2\}_3]_2$ and 1,4-benzoquinone. Cerous $[Ce\{N(SiHMe_2)_2\}_3]_2$ (20 mg, 0.019 mmol) and 1,4-benzoquinone (2.0 mg, 0.019 mmol) were combined in each C_6D_6 and THF-d_8 (NMR-scale experiments, using a J. Young-valved NMR tube). An immediate colour change from pale yellow to dark brown was observed in both solvents. ^{1}H NMR spectra were recorded directly after addition of 1,4-benzoquinone and after 24 h to investigate on the stability of the formed product.

Reaction between $[Ce\{N(SiHMe_2)_2\}_3]_2$ and tetramethyl-1,4-benzoquinone. Cerous $[Ce\{N(SiHMe_2)_2\}_3]_2$ (20 mg, 0.019 mmol) and tetramethyl-1,4-benzoquinone (3.1 mg, 0.019 mmol) were combined in each C_6D_6 and THF-d_8 (NMR-scale experiments, using a J. Young-valved NMR tube). An immediate colour change from pale yellow to dark brownish green was observed in both solvents. ^{1}H NMR spectra were recorded directly after addition of tetramethyl-1,4-benzoquinone and after 24 h to investigate on the stability of the formed product.

NMR Spectra (solvent signals are marked with *)

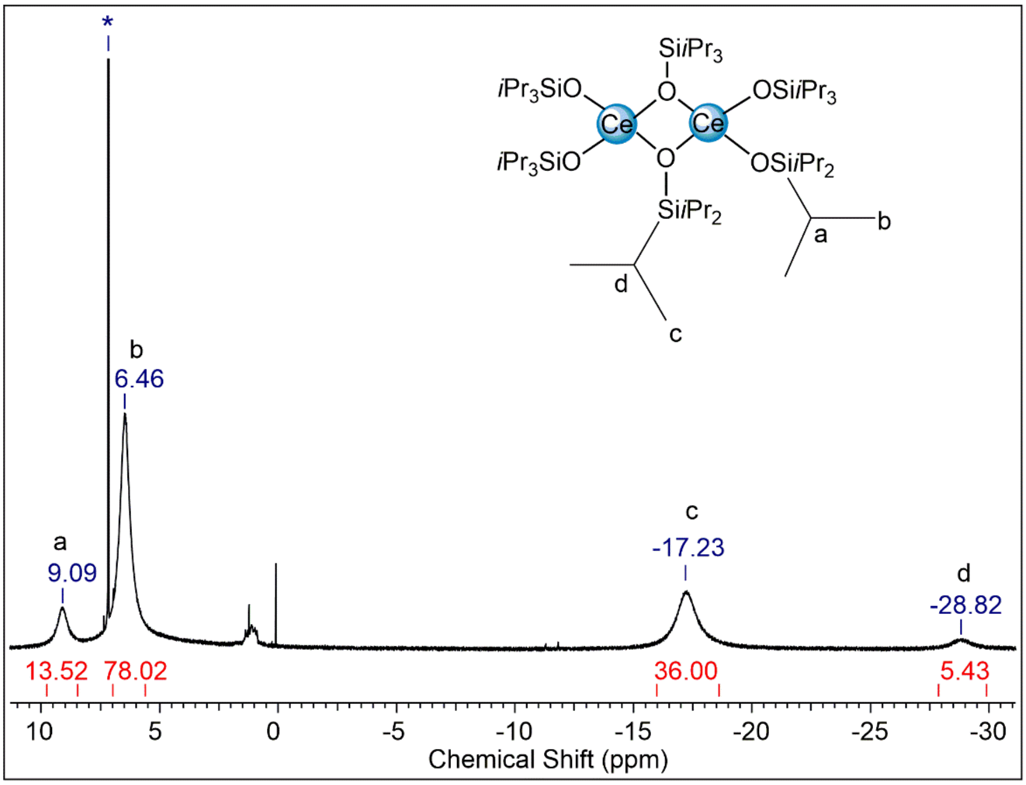

Figure S1. ^{1}H NMR spectrum (400.13 MHz, C_6D_6, 26 °C) of $[Ce(OSi\textit{i}Pr_3)_3]_2$ (**3**).

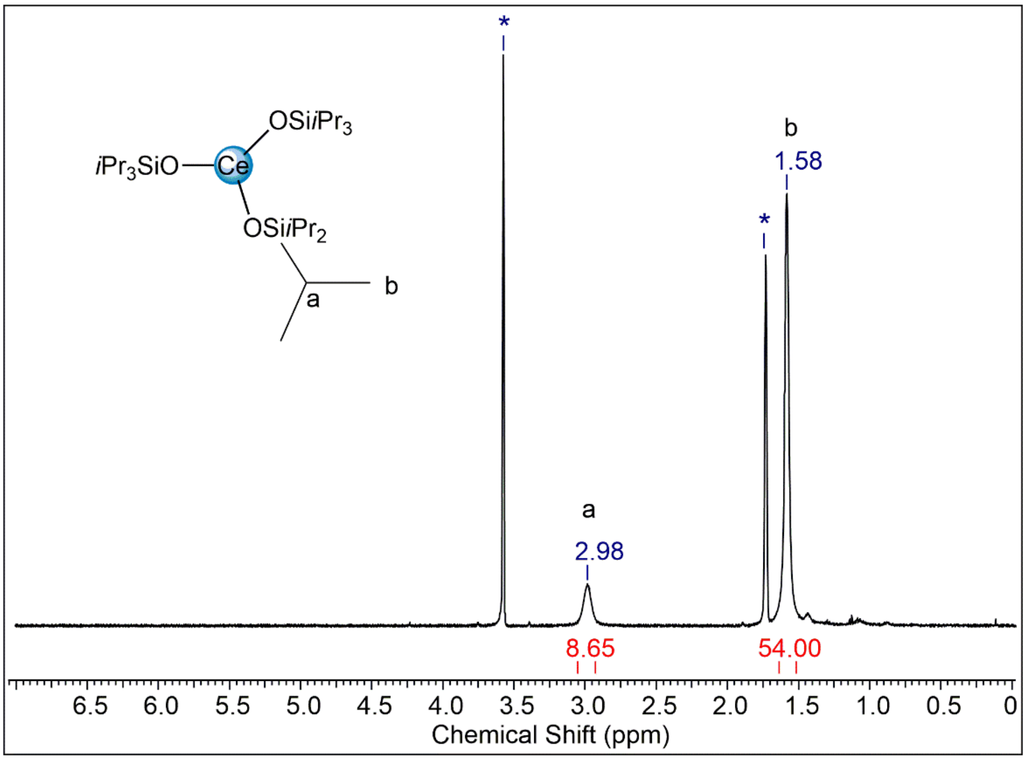

Figure S2. ^{1}H NMR spectrum (400.13 MHz, THF-d_8, 26 °C) of $[Ce(OSi\textit{i}Pr_3)_3]_2$ (**3**).

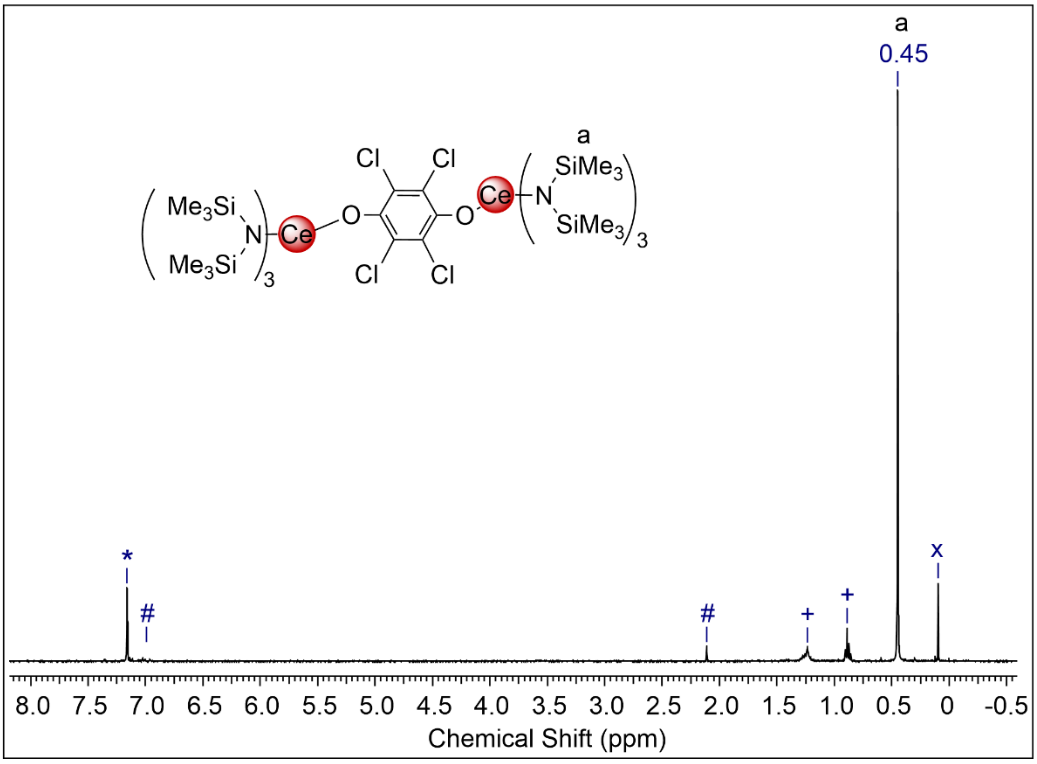

Figure S3. ^{1}H NMR spectrum (400.13 MHz, C_6D_6, 26 °C) of $[Ce\{N(SiMe_3)_2\}_3]_2(\mu_2\text{-}O_2C_6Cl_4)$ (**4**$^{\mathrm{Cl4hq}}$). Trace impurities of toluene, *n*-hexane and $[KN(SiMe_3)_2]$ are marked with #, + and x.

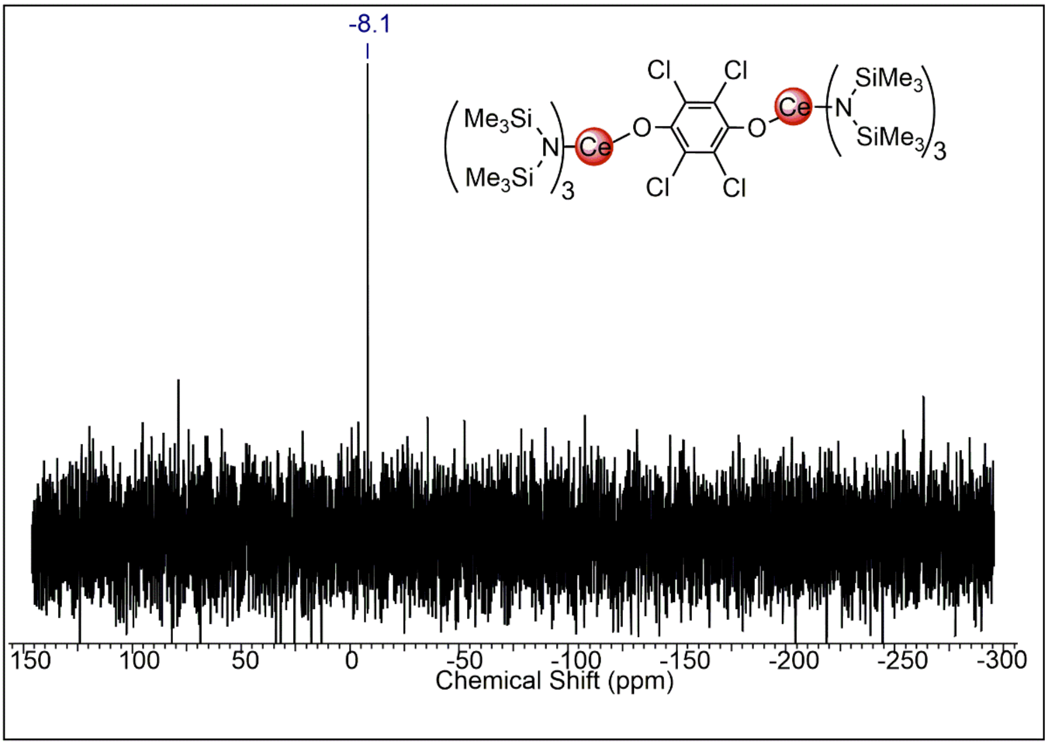

Figure S4. ^{29}Si DEPT45 NMR spectrum (69.63 MHz, C_6D_6, 26 °C) of $[Ce\{N(SiMe_3)_2\}_3]_2(\mu_2\text{-}O_2C_6Cl_4)$ (**4**$^{\mathrm{Cl4hq}}$).

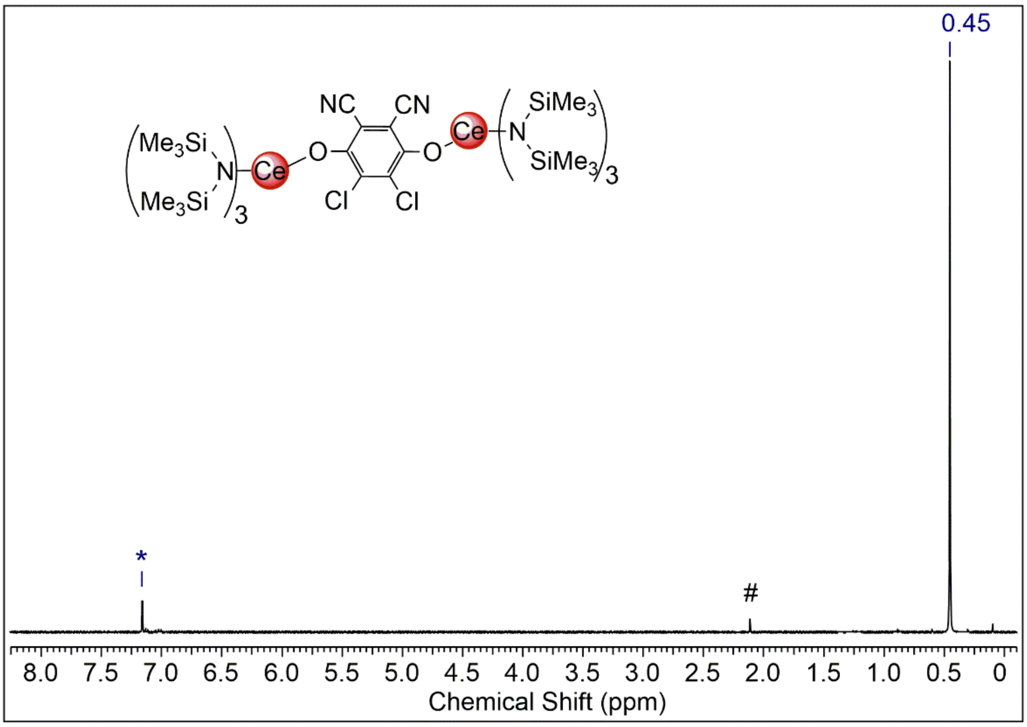

Figure S5. ^{1}H NMR spectrum (500.13 MHz, C_6D_6, 26 °C) of $[Ce\{N(SiMe_3)_2\}_3]_2(\mu_2\text{-}O_2C_6Cl_2(CN)_2)$ (**4**^{ddhq}). Trace impurities of toluene are marked with #.

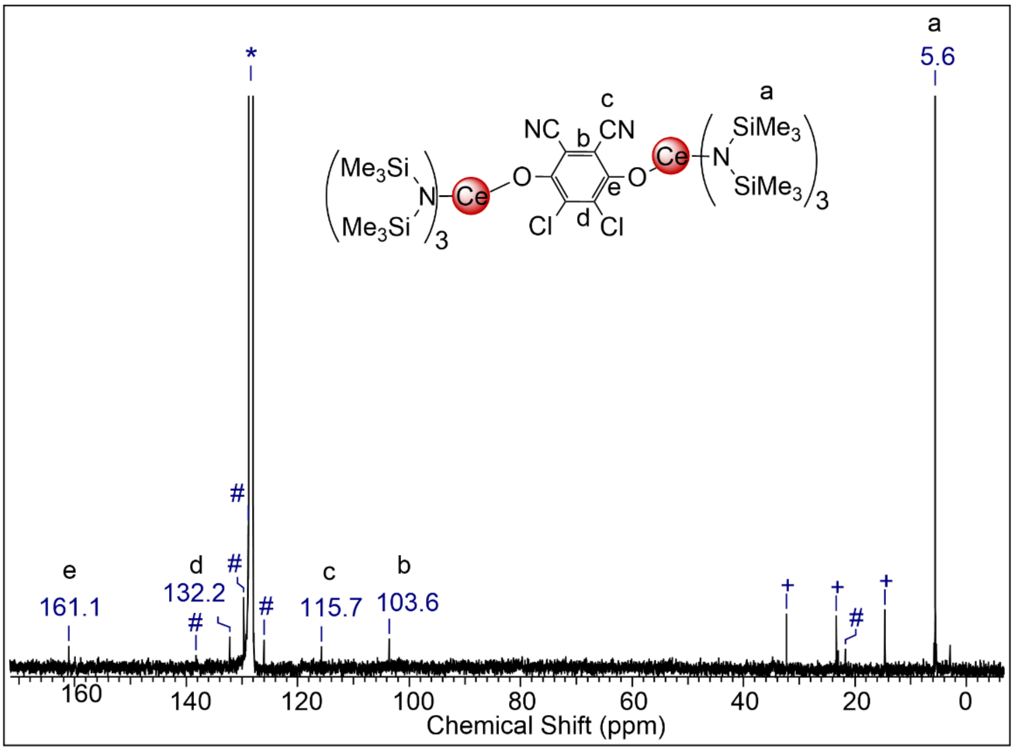

Figure S6. $^{13}C\{^{1}H\}$ NMR spectrum (125.76 MHz, C_6D_6, 26 °C) of $[Ce\{N(SiMe_3)_2\}_3]_2(\mu_2\text{-}O_2C_6Cl_2(CN)_2)$ (**4**^{ddhq}). Trace impurities of toluene and *n*-hexane are marked with # and +.

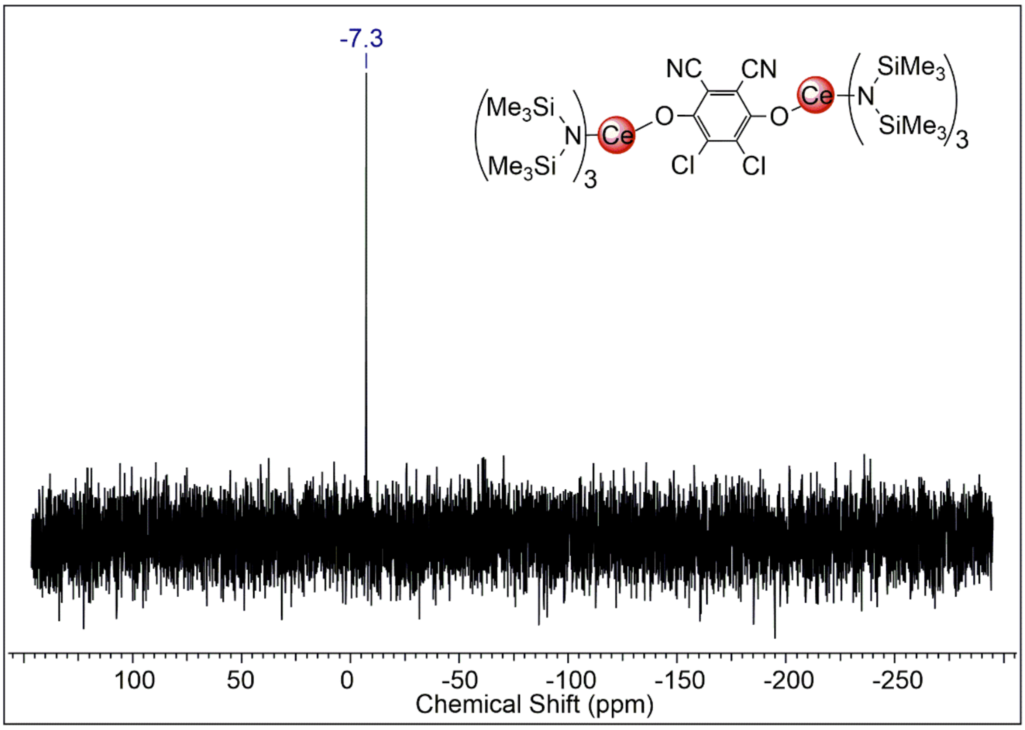

Figure S7. ^{29}Si DEPT45 NMR spectrum (69.63 MHz, C_6D_6, 26 °C) of $[Ce\{N(SiMe_3)_2\}_3]_2(\mu_2\text{-}O_2C_6Cl_2(CN)_2)$ (**4**$^{\mathbf{ddhq}}$).

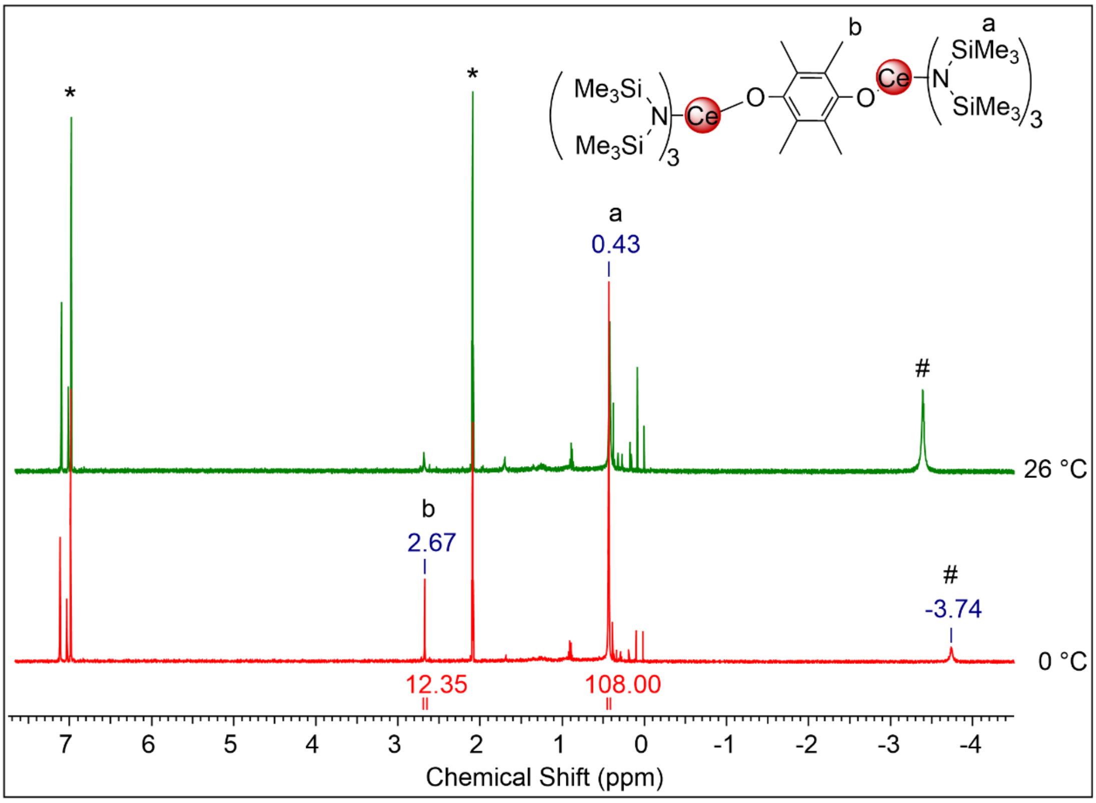

Figure S8. Stacked ^{1}H NMR spectra (500.13 MHz, toluene-d_8) of $[Ce\{N(SiMe_3)_2\}_3]_2(\mu_2\text{-}O_2C_6Me_4)$ (**4**$^{\mathbf{Me4hq}}$) at 0 °C (red) and 26 °C (green), indicating decomposition in solution and formation of $Ce[N(SiMe_3)_2]_3$ (marked with #).

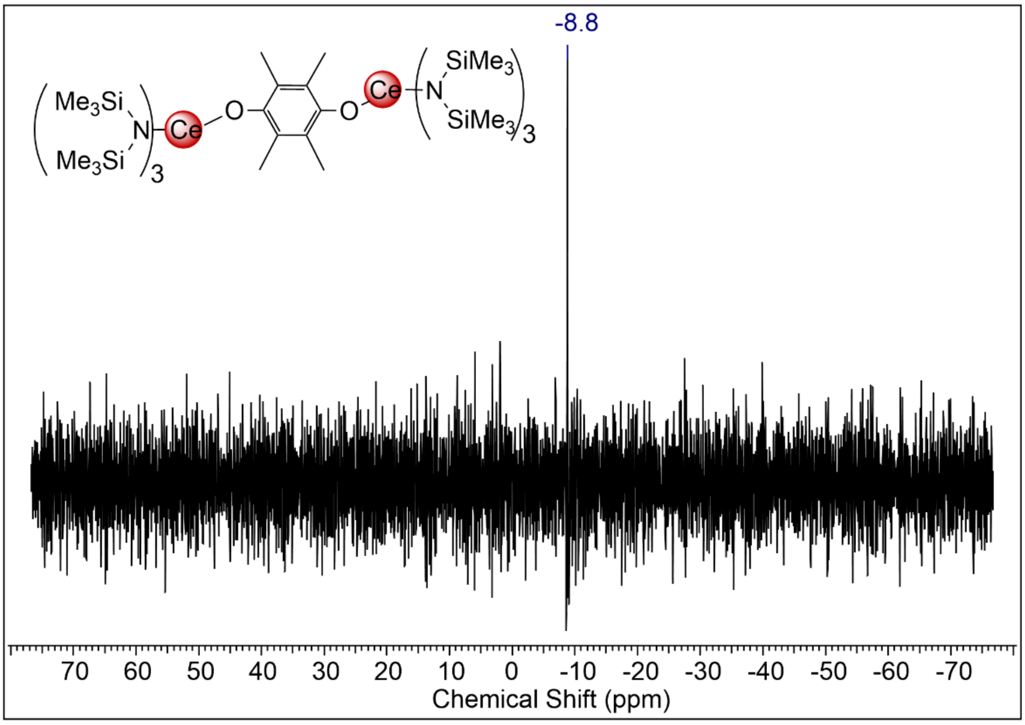

Figure S9. ^{29}Si INEPTND NMR spectrum (99.36 MHz, C_6D_6, 26 °C) of $[Ce\{N(SiMe_3)_2\}_3]_2(\mu_2\text{-}O_2C_6Me_4)$ ($\mathbf{4}^{\mathbf{Me4hq}}$).

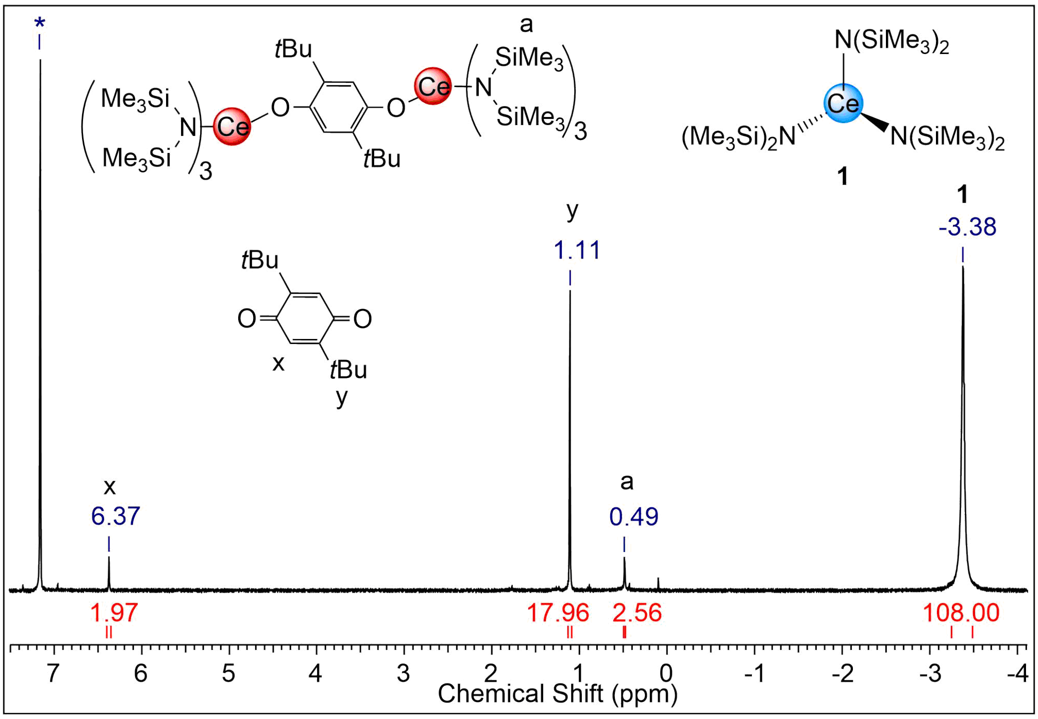

Figure S10. ^{1}H NMR spectrum (400.13 MHz, C_6D_6, 26 °C) of $[Ce\{N(SiMe_3)_2\}_3]_2(\mu_2\text{-}O_2C_6tBu_2H_2)$ ($\mathbf{4}^{\mathbf{tBu2hq}}$).

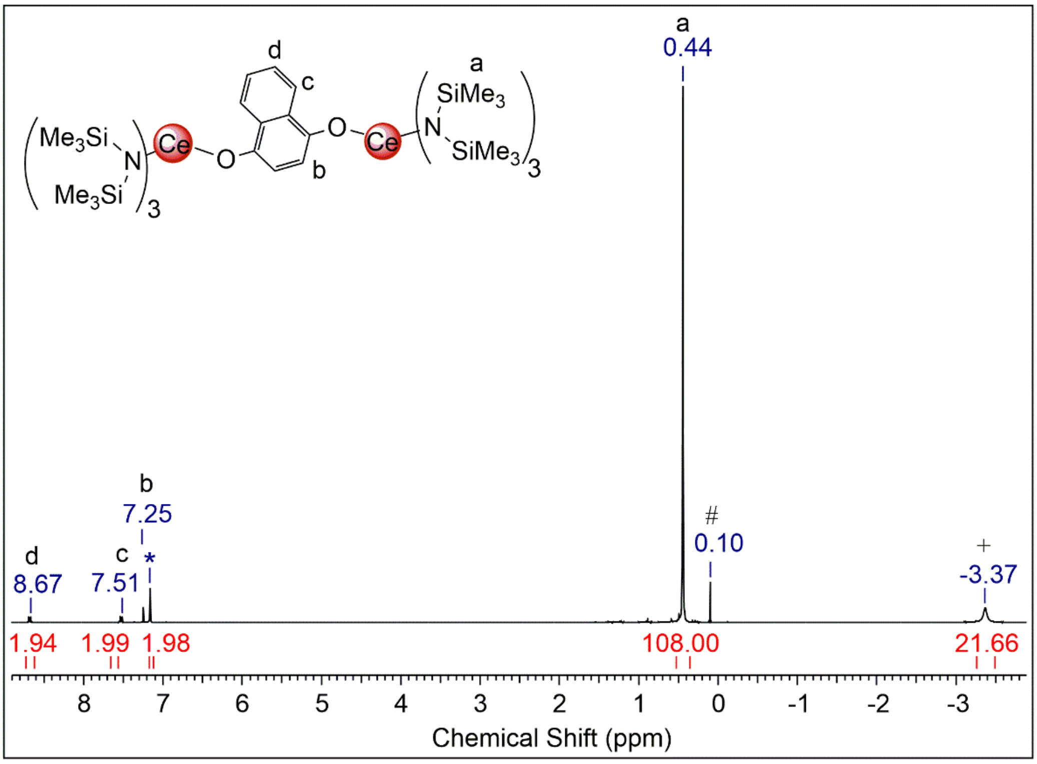

Figure S11. ^{1}H NMR spectrum (400.13 MHz, C_6D_6, 26 °C) of $[Ce\{N(SiMe_3)_2\}_3]_2(\mu_2\text{-}O_2C_{10}H_6)$ (**4**^{nhq}). Impurities of $KN(SiMe_3)_2$ are marked with # and $Ce[N(SiMe_3)_2]_3$ with +.

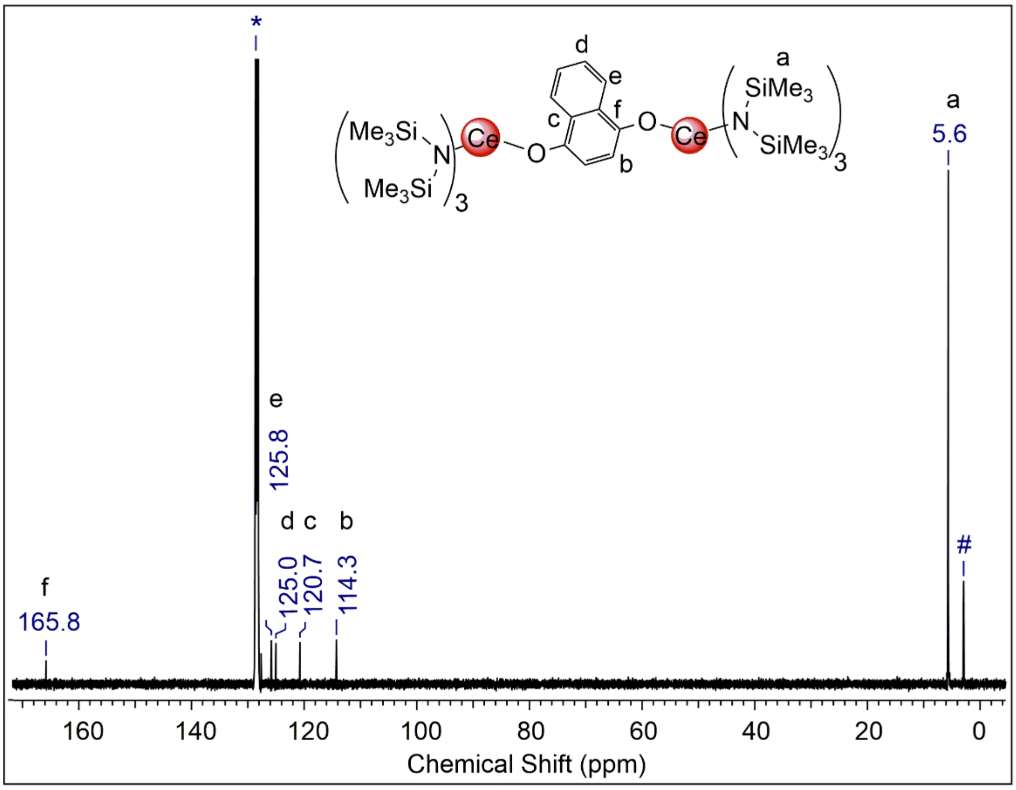

Figure S12. $^{13}C\{^{1}H\}$ NMR spectrum (100.61 MHz, C_6D_6, 26 °C) of $[Ce\{N(SiMe_3)_2\}_3]_2(\mu_2\text{-}O_2C_{10}H_6)$ (**4**^{nhq}). Impurities of $KN(SiMe_3)_2$ are marked with #.

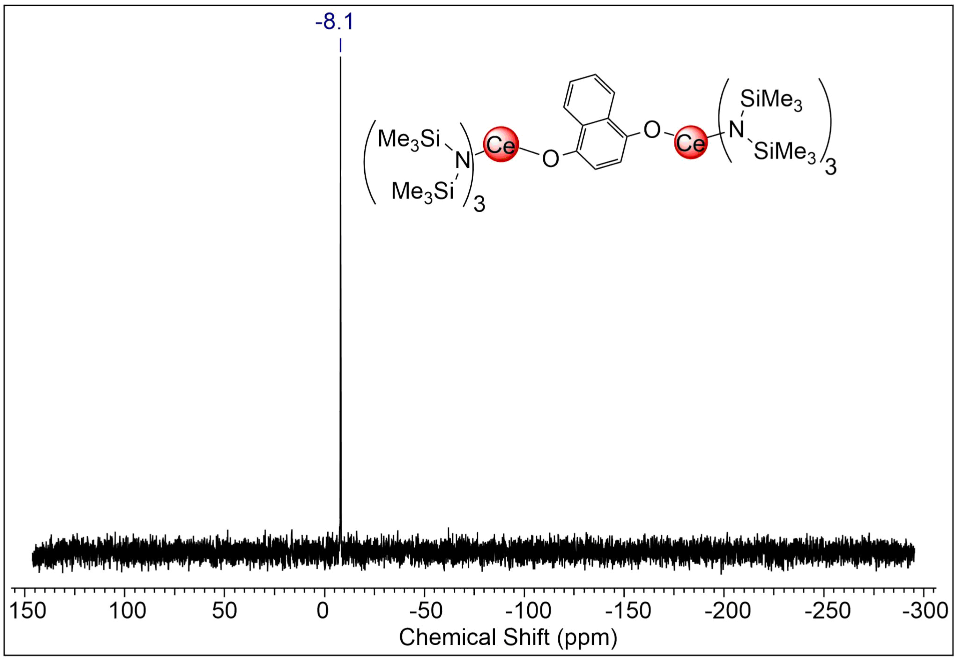

Figure S13. ^{29}Si DEPT45 NMR spectrum (69.63 MHz, C_6D_6, 26 °C) of $[Ce\{N(SiMe_3)_2\}_3]_2(\mu_2\text{-}O_2C_{10}H_6)$ (**4**^{nhq}).

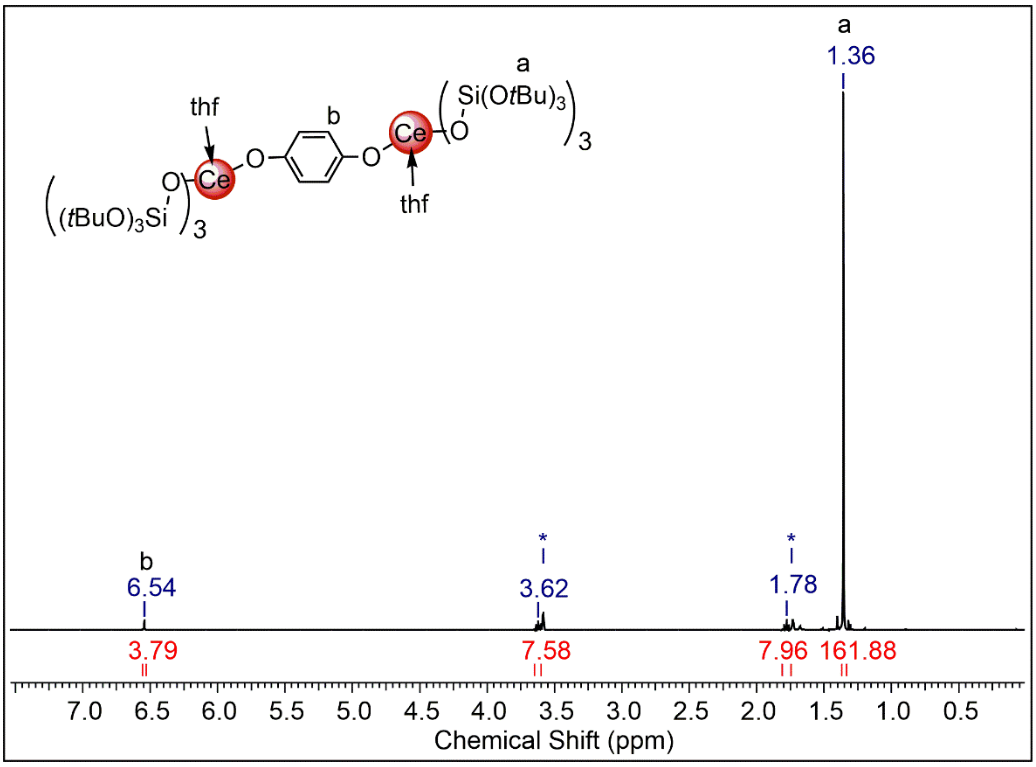

Figure S14. ^{1}H NMR spectrum (400.13 MHz, THF-d_8, 26 °C) of $[Ce\{OSi(OtBu)_3\}_3(thf)]_2(\mu_2\text{-}O_2C_6H_4)$ (**5**^{hq}).

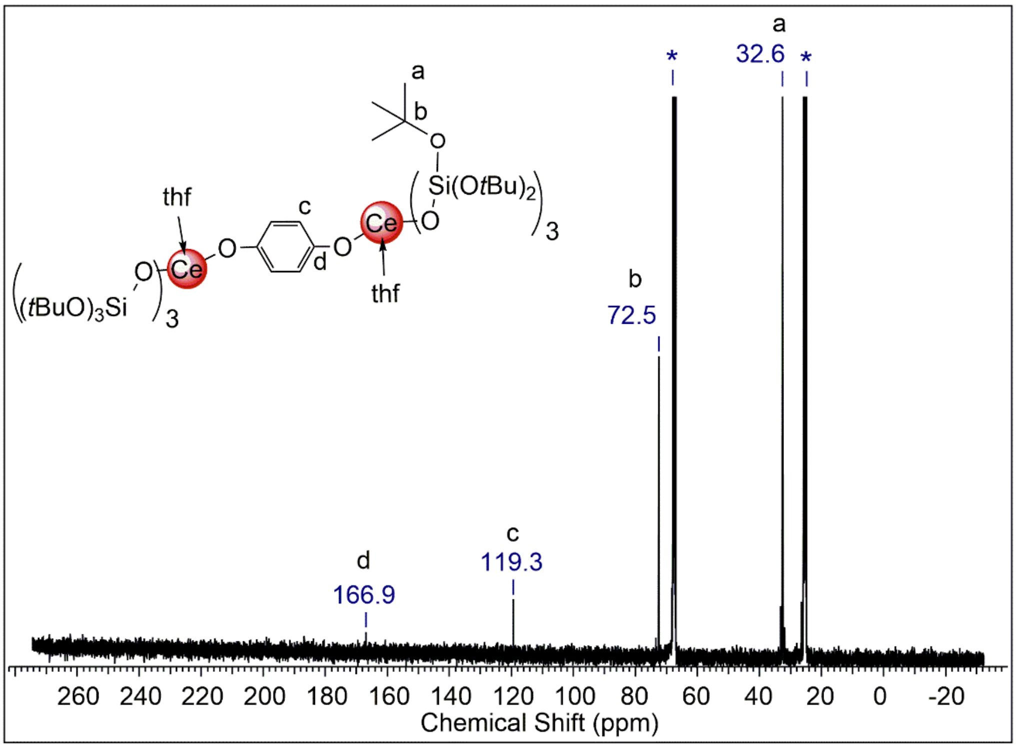

Figure S15. $^{13}C\{^1H\}$ NMR spectrum (100.61 MHz, THF-d_8, 26 °C) of $[Ce\{OSi(OtBu)_3\}_3(thf)]_2(\mu_2\text{-}O_2C_6H_4)$ (**5**[hq]).

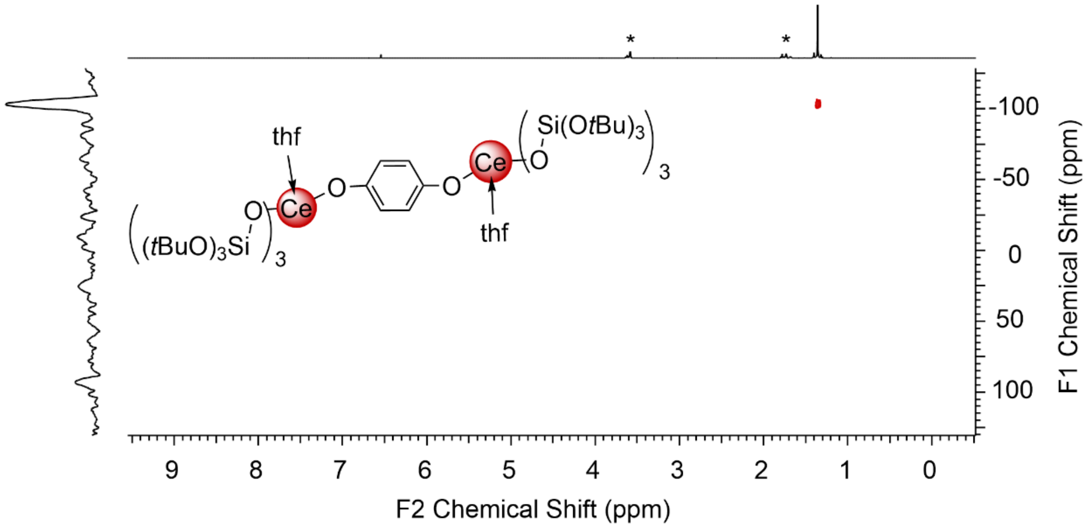

Figure S16. $^1H\text{-}^{29}Si$ HSQC NMR spectrum (99.36 MHz, THF-d_8, 26 °C) of $[Ce\{OSi(OtBu)_3\}_3(thf)]_2(\mu_2\text{-}O_2C_6H_4)$ (**5**[hq]).

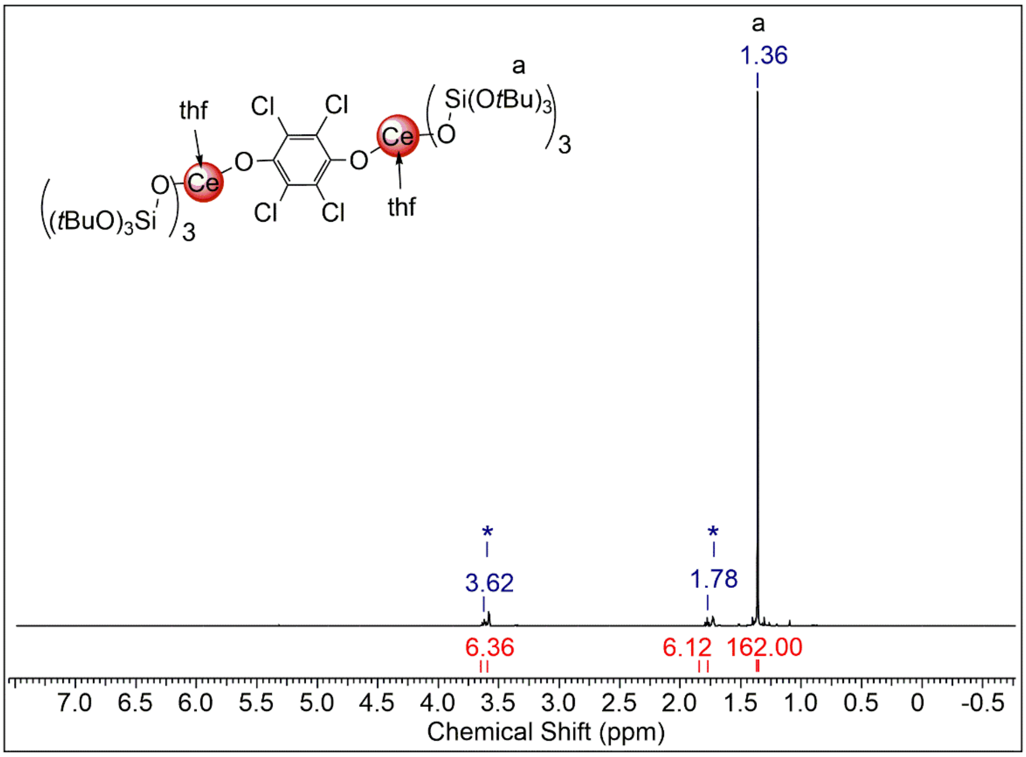

Figure S17. ^{1}H NMR spectrum (400.13 MHz, THF-d_8, 26 °C) of [Ce{OSi(O*t*Bu)$_3$}$_3$(thf)]$_2$(μ_2-O$_2$C$_6$Cl$_4$) (**5**$^{\mathbf{Cl4hq}}$).

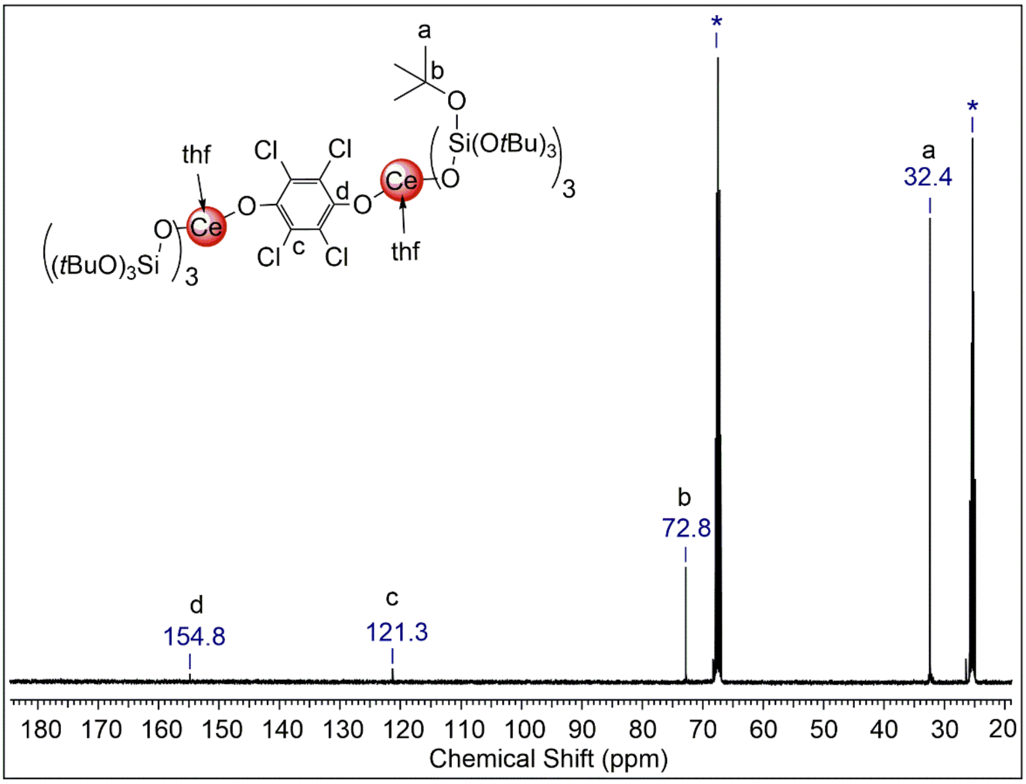

Figure S18. ^{13}C{^{1}H} NMR spectrum (100.61 MHz, THF-d_8, 26 °C) [Ce{OSi(O*t*Bu)$_3$}$_3$(thf)]$_2$(μ_2-O$_2$C$_6$Cl$_4$) (**5**$^{\mathbf{Cl4hq}}$).

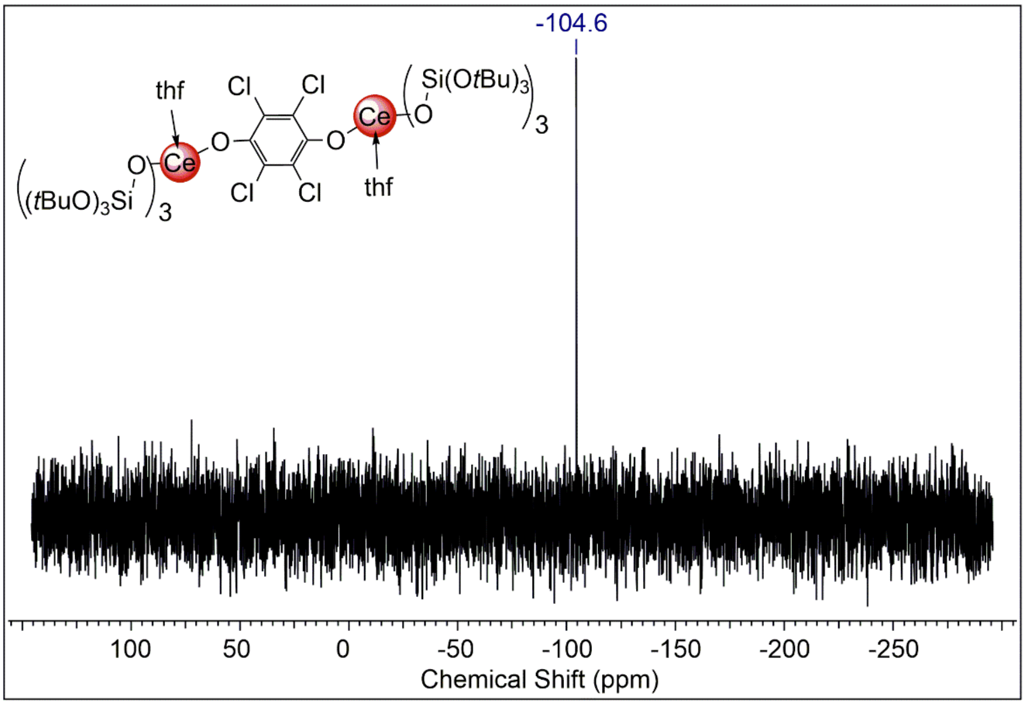

Figure S19. ^{29}Si DEPT45 NMR spectrum (69.63 MHz, THF-d_8, 26 °C) of [Ce{OSi(O*t*Bu)$_3$}$_3$(thf)]$_2$(μ_2-$O_2C_6Cl_4$) (**5**$^{\mathbf{Cl4hq}}$).

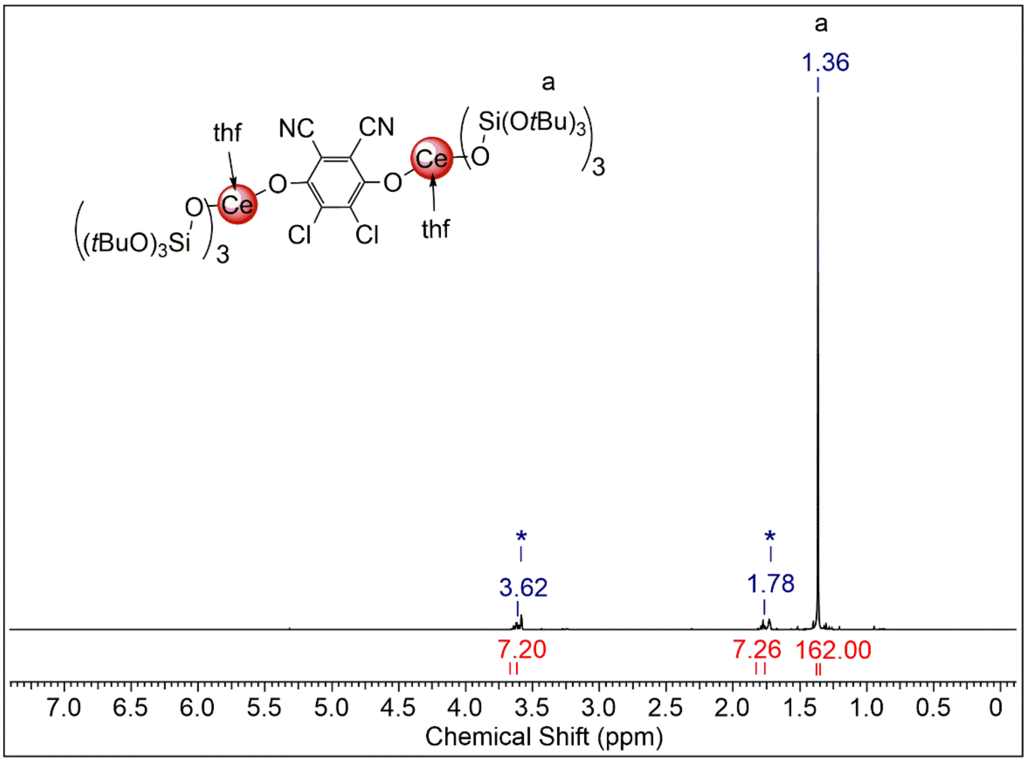

Figure S20. ^{1}H NMR spectrum (400.13 MHz, THF-d_8, 26 °C) of [Ce{OSi(O*t*Bu)$_3$}$_3$(thf)]$_2$(μ_2-$O_2C_6Cl_2(CN)_2$) (**5**$^{\mathbf{ddhq}}$).

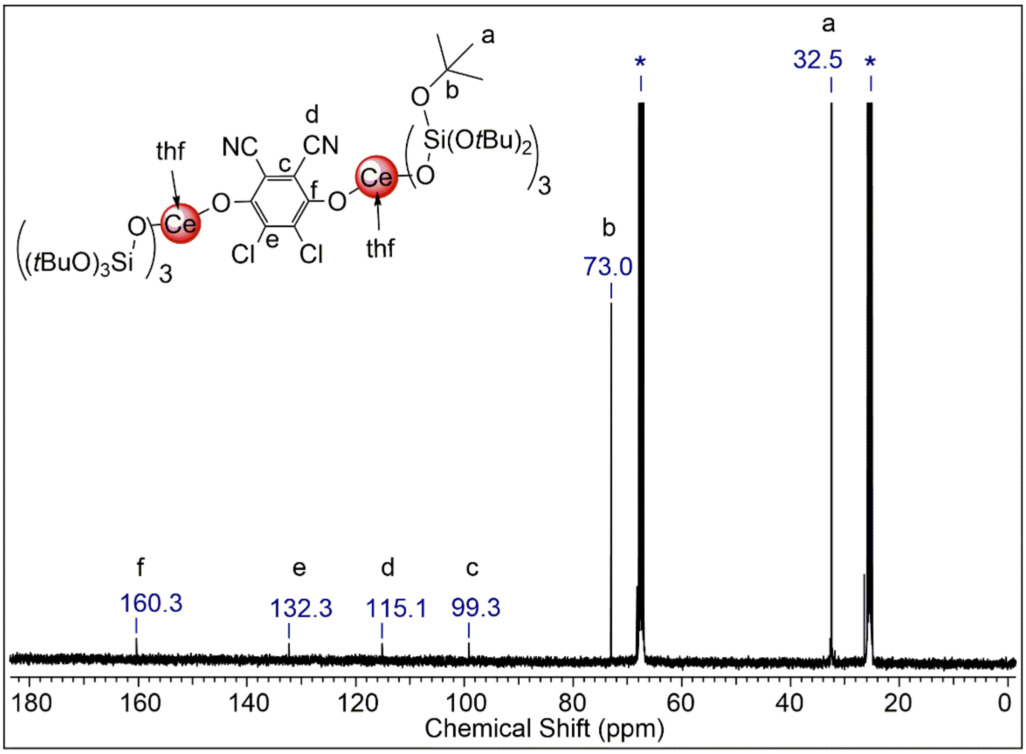

Figure S21. $^{13}C\{^{1}H\}$ NMR spectrum (100.61 MHz, THF-d_8, 26 °C) [Ce{OSi(O*t*Bu)$_3$}$_3$(thf)]$_2$(μ_2-O$_2$C$_6$Cl$_2$(CN)$_2$) (**5**[ddhq]).

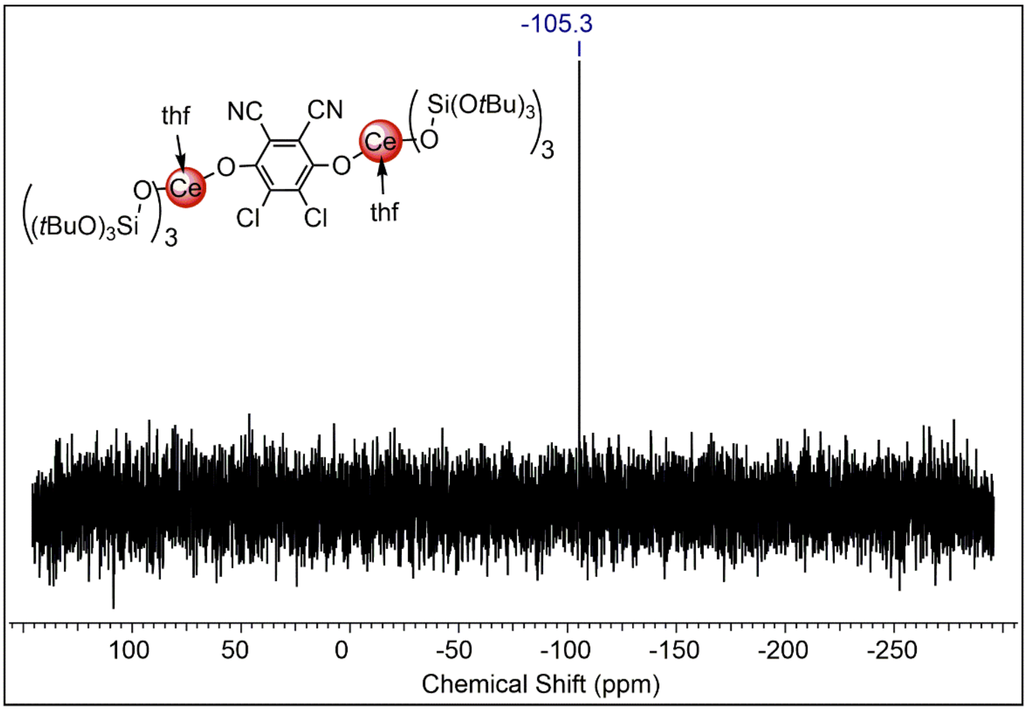

Figure S22. ^{29}Si DEPT45 NMR spectrum (69.63 MHz, THF-d_8, 26 °C) of [Ce{OSi(O*t*Bu)$_3$}$_3$(thf)]$_2$(μ_2-O$_2$C$_6$Cl$_2$(CN)$_2$) (**5**[ddhq]).

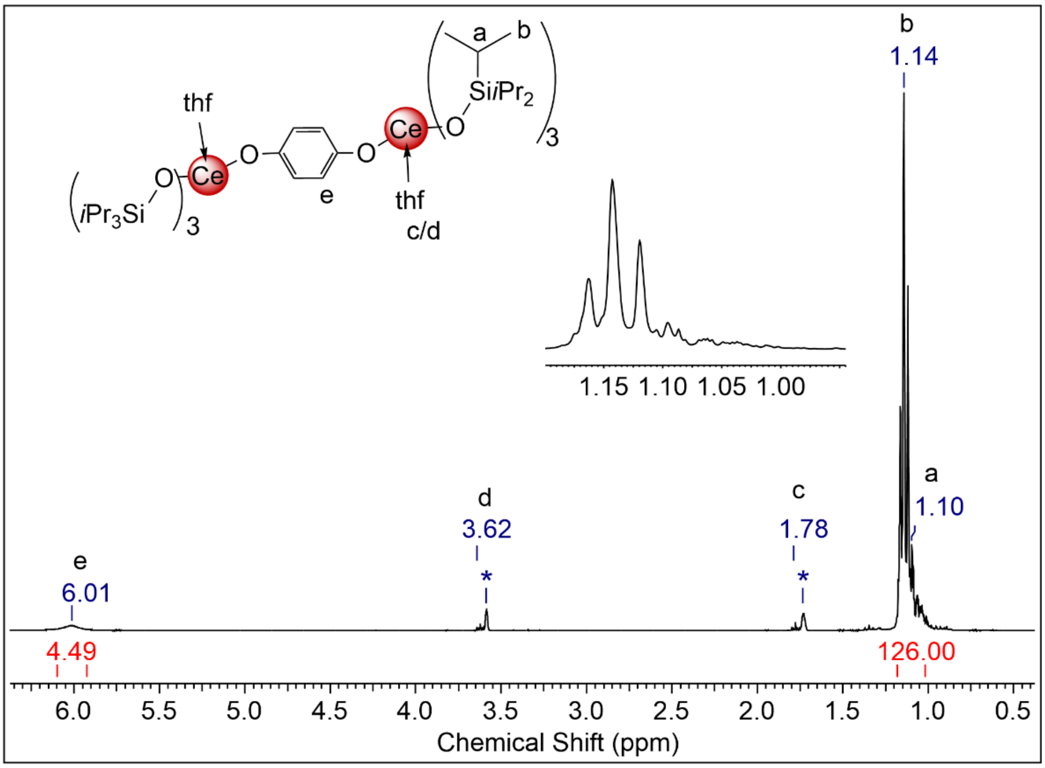

Figure S23. ^{1}H NMR spectrum (400.13 MHz, THF-d_8, 26 °C) of $[Ce(OSi\textit{i}Pr_3)_3(thf)]_2(\mu_2\text{-}O_2C_6H_4)$ (**6**$^{\mathbf{hq}}$).

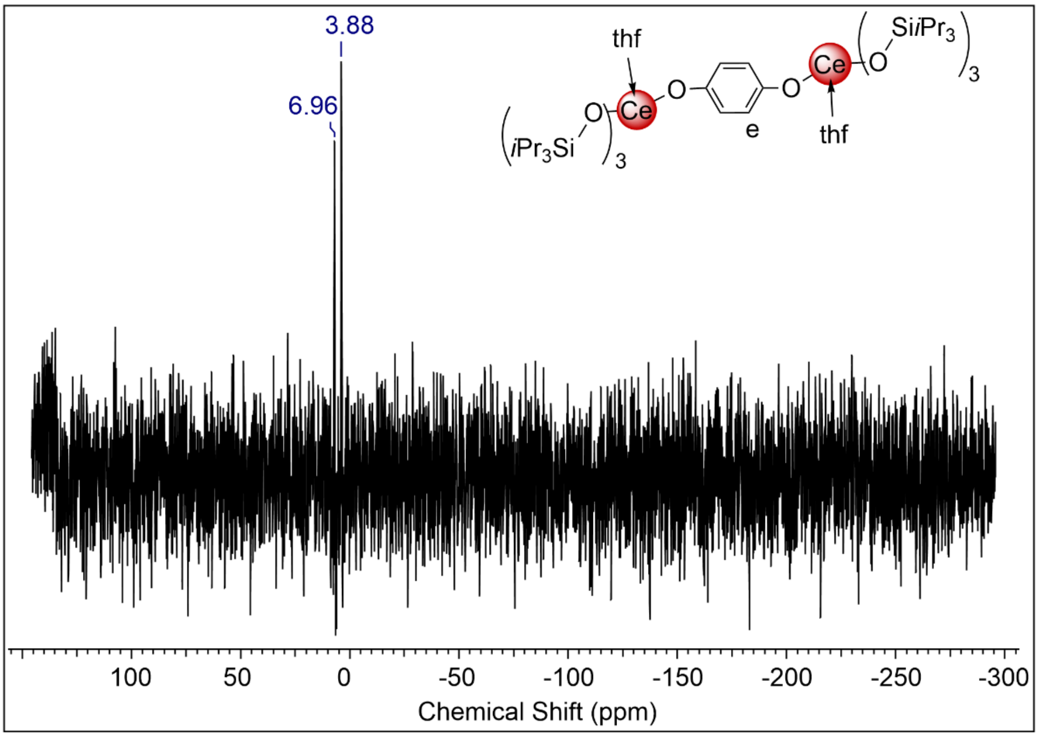

Figure S24. ^{29}Si INEPTND NMR spectrum (69.63 MHz, THF-d_8, 26 °C) of $[Ce(OSi\textit{i}Pr_3)_3(thf)]_2(\mu_2\text{-}O_2C_6H_4)$ (**6**$^{\mathbf{hq}}$).

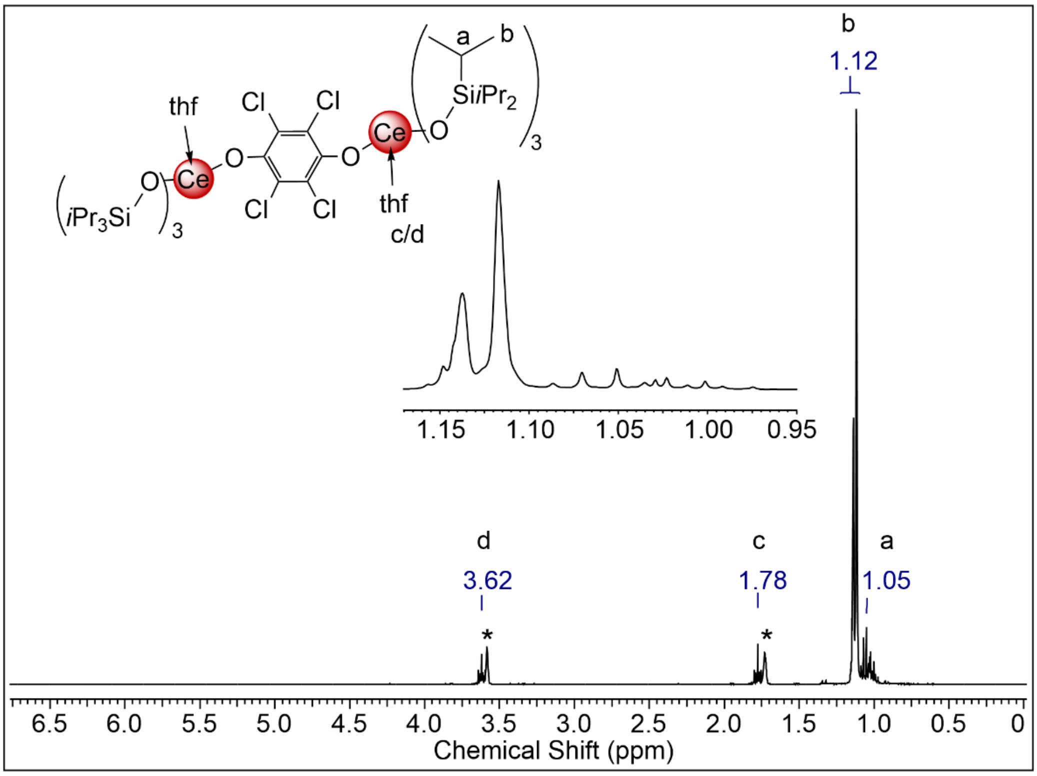

Figure S25. ^{1}H NMR spectrum (400.13 MHz, THF-d_8, 26 °C) of $[Ce(OSi\mathit{i}Pr_3)_3(thf)]_2(\mu_2\text{-}O_2C_6Cl_4)$ (**6**$^{\mathbf{Cl4hq}}$).

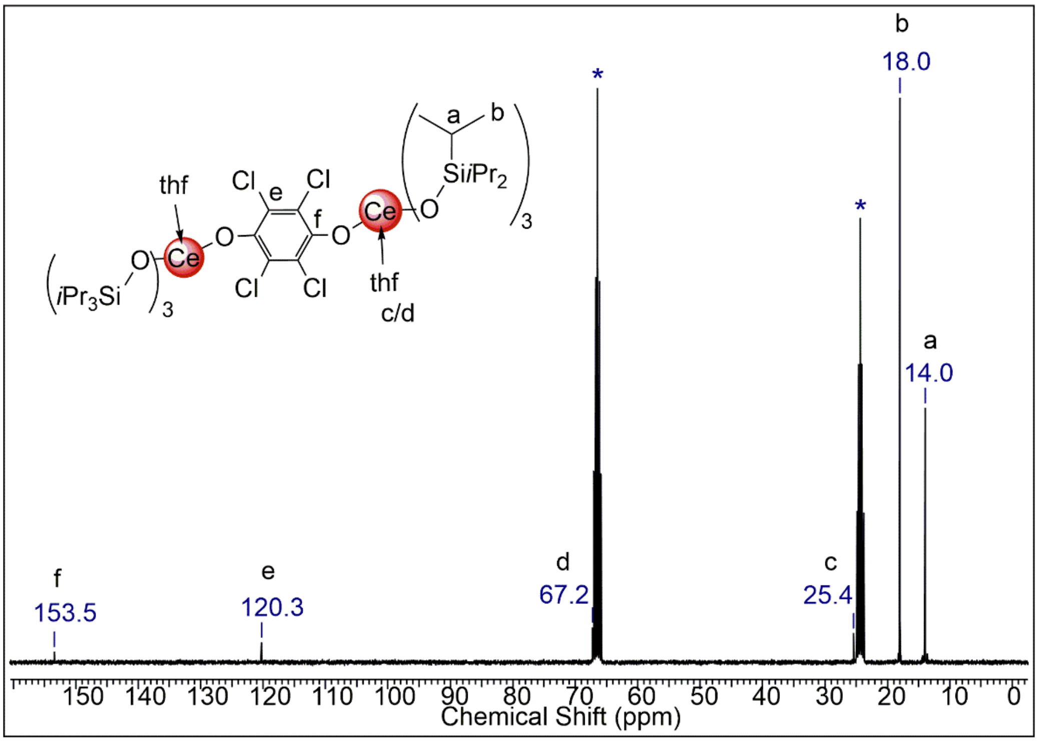

Figure S26. $^{13}C\{^{1}H\}$ NMR spectrum (100.61 MHz, THF-d_8, 26 °C) $[Ce(OSi\mathit{i}Pr_3)_3(thf)]_2(\mu_2\text{-}O_2C_6Cl_4)$ (**6**$^{\mathbf{Cl4hq}}$).

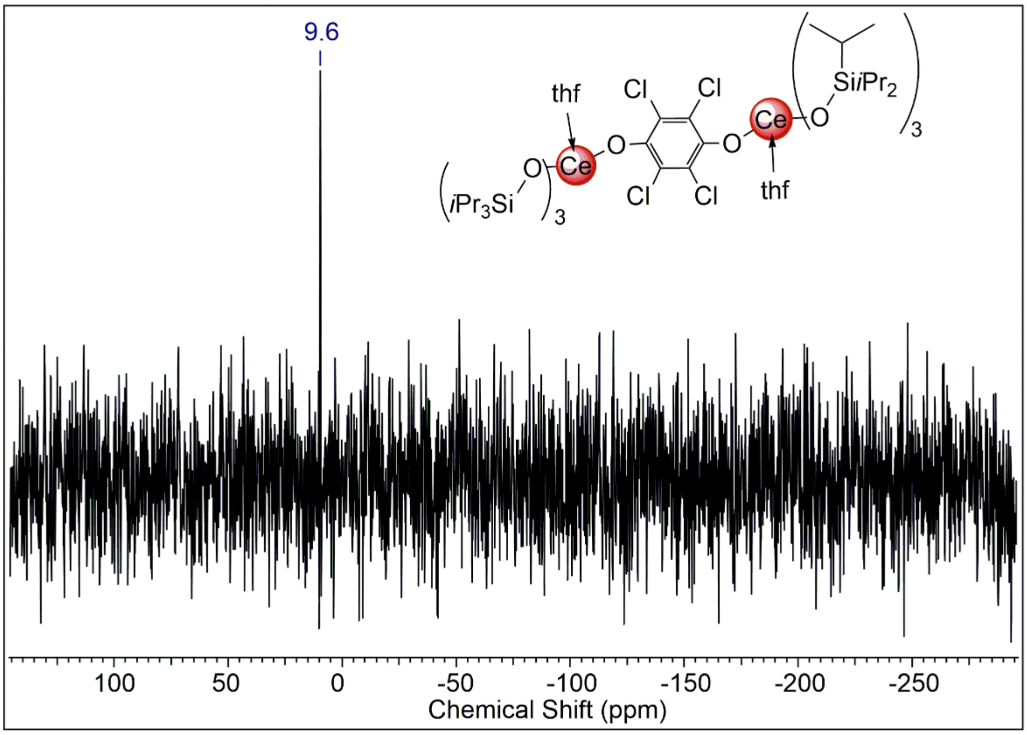

Figure S27. ^{29}Si INEPTND NMR spectrum (69.63 MHz, THF-d_8, 26 °C) of [Ce(OSi*i*Pr$_3$)$_3$(thf)]$_2$(μ_2-O$_2$C$_6$Cl$_4$) (**6**$^{\mathbf{Cl4hq}}$).

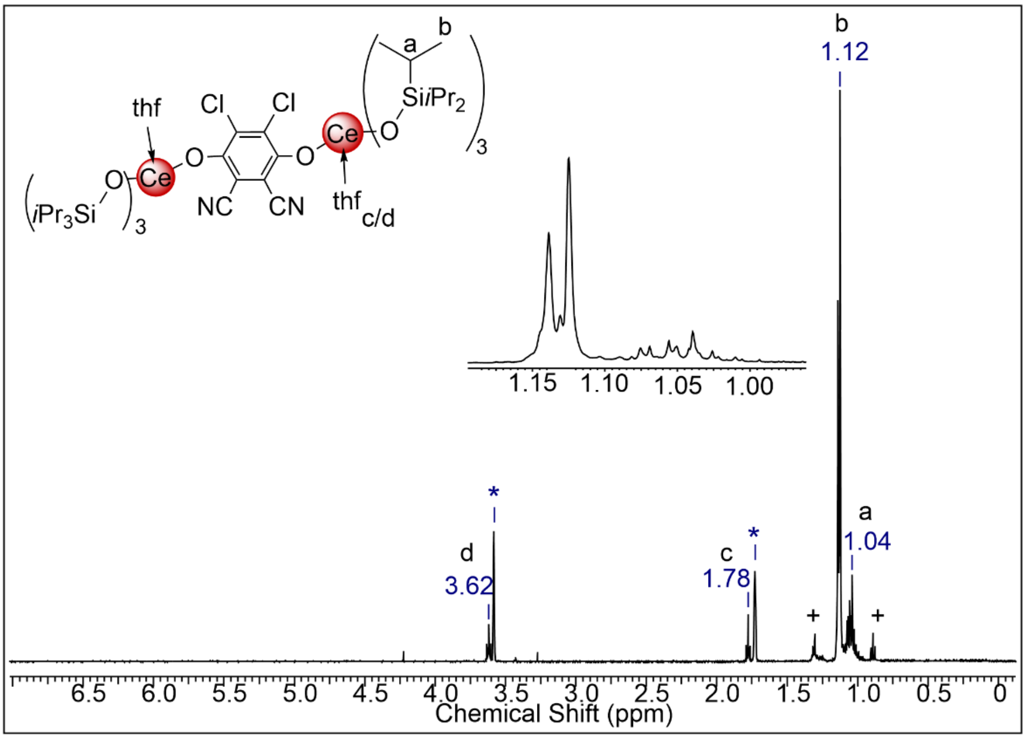

Figure S28. ^{1}H NMR spectrum (500.13 MHz, THF-d_8, 26 °C) of [Ce(OSi*i*Pr$_3$)$_3$(thf)]$_2$(μ_2-O$_2$C$_6$Cl$_2$(CN)$_2$) (**6**$^{\mathbf{ddhq}}$).

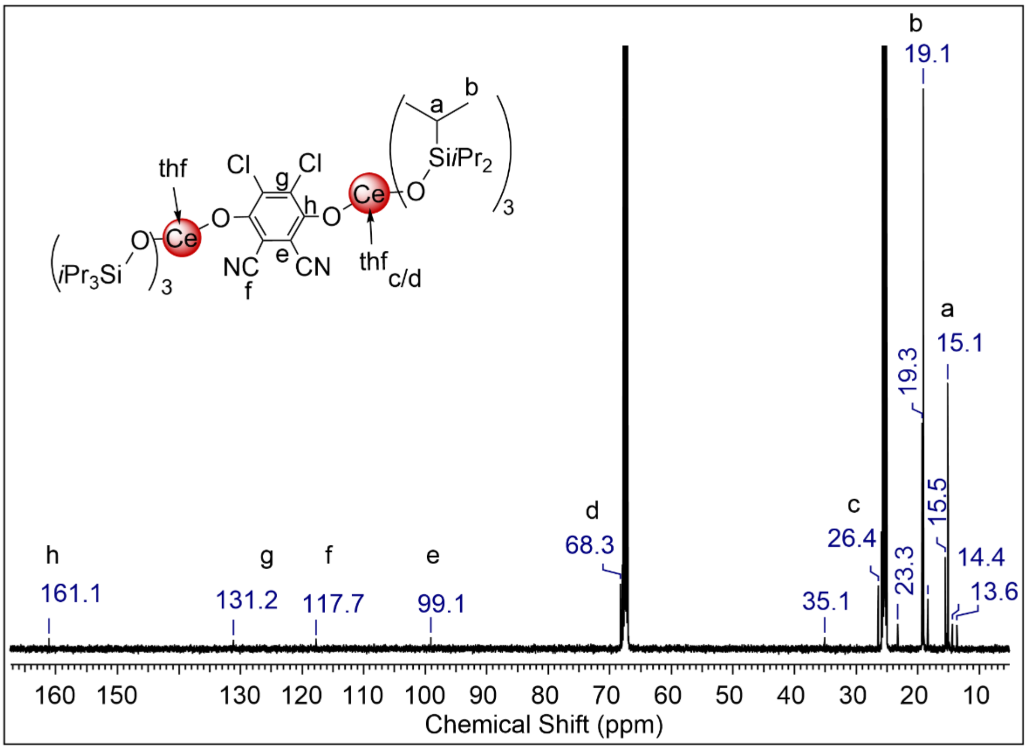

Figure S29. $^{13}C\{^{1}H\}$ NMR spectrum (125.76 MHz, THF-d_8, 26 °C) $[Ce(OSi\textit{i}Pr_3)_3(thf)]_2(\mu_2\text{-}O_2C_6Cl_2(CN)_2)$ ($\mathbf{6}^{\mathbf{ddhq}}$).

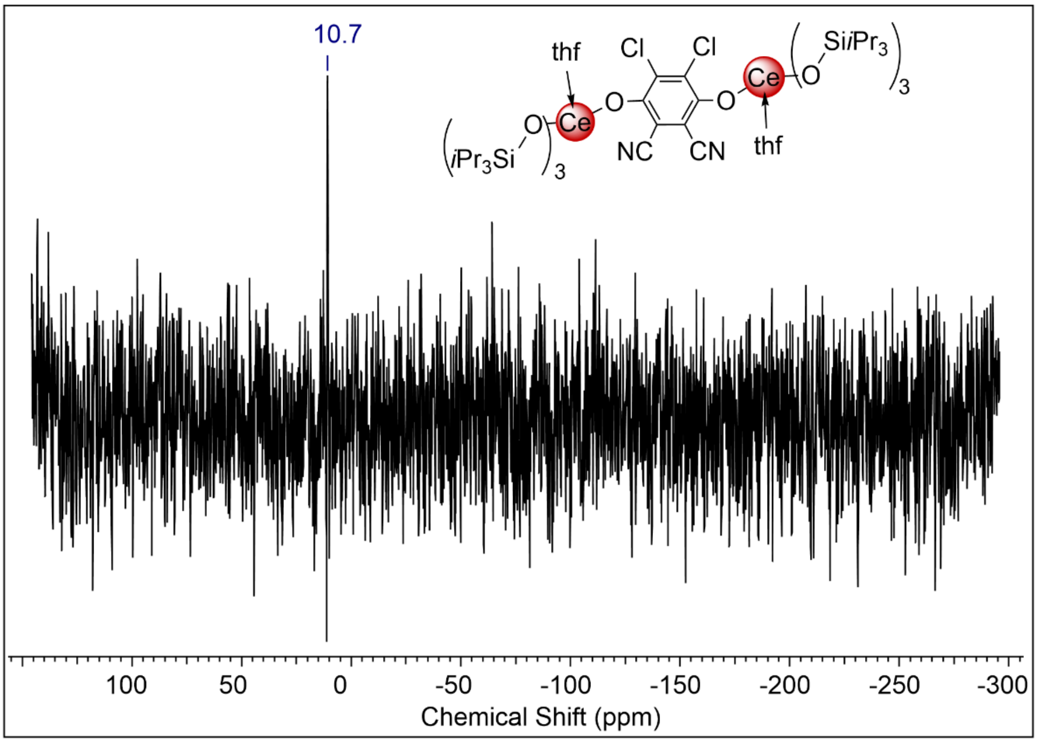

Figure S30. ^{29}Si INEPTND NMR spectrum (69.63 MHz, THF-d_8, 26 °C) of $[Ce(OSi\textit{i}Pr_3)_3(thf)]_2(\mu_2\text{-}O_2C_6Cl_2(CN)_2)$ ($\mathbf{6}^{\mathbf{ddhq}}$).

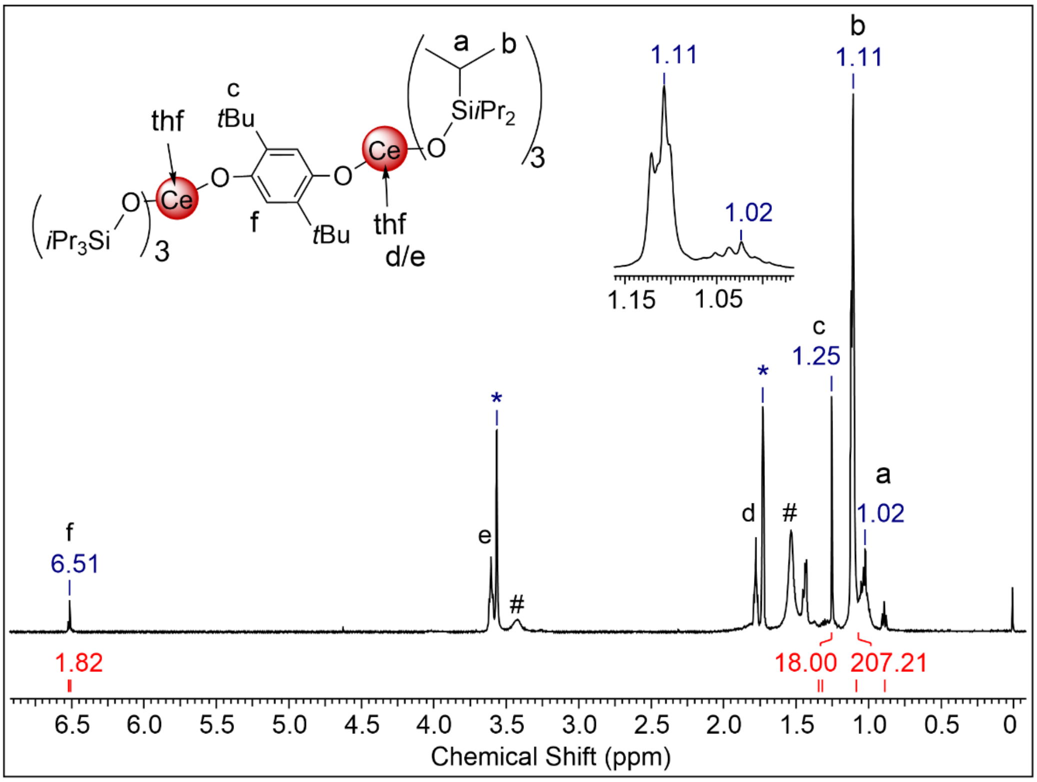

Figure S31. ^{1}H NMR spectrum (500.13 MHz, THF-d_8, 26 °C) of [Ce(OSi*i*Pr$_3$)$_3$(thf)]$_2$(μ_2-O$_2$C$_6$*t*Bu$_2$H$_2$) (**6**$^{t\mathrm{Bu2hq}}$). The high integral for the *iso*propyl groups indicates the formation of a second tetravalent cerium tri*iso*propyl siloxide species. Impurities of [Ce(OSi*i*Pr$_3$)$_3$]$_2$ (**3**) are marked with #.

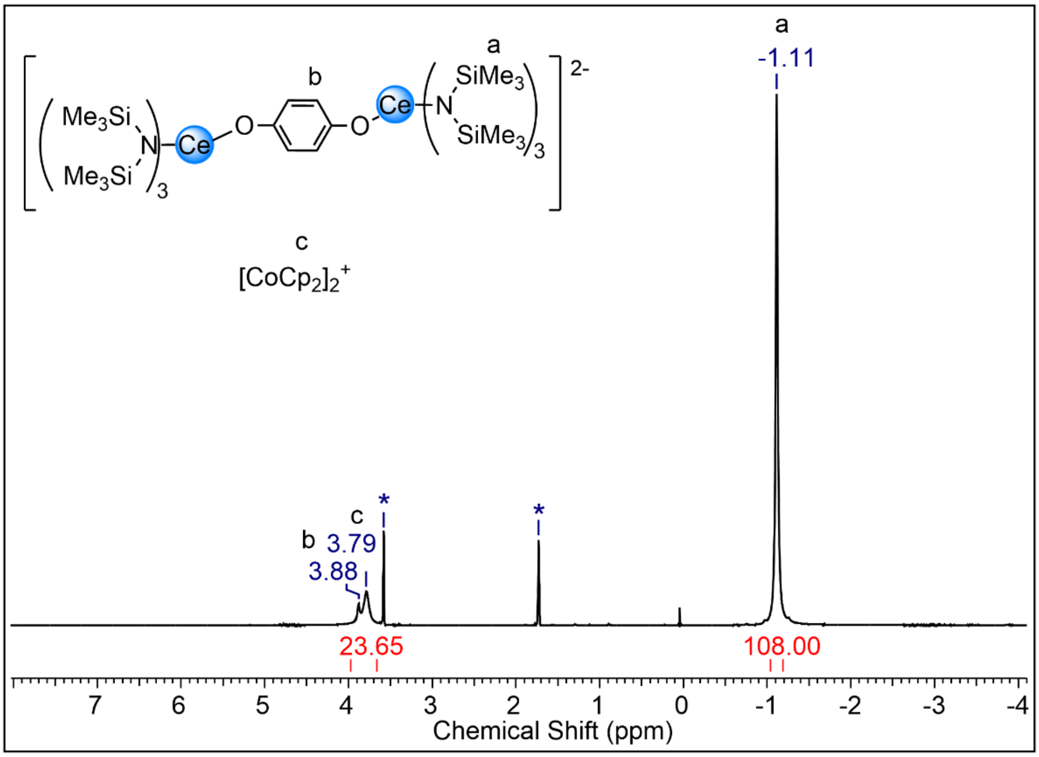

Figure S32. ^{1}H NMR spectrum (400.13 MHz, THF-d_8, 26 °C) of [(Ce{N(SiMe$_3$)$_2$}$_3$)$_2$(μ_2-O$_2$C$_6$H$_4$)][CoCp$_2$]$_2$ (**7**).

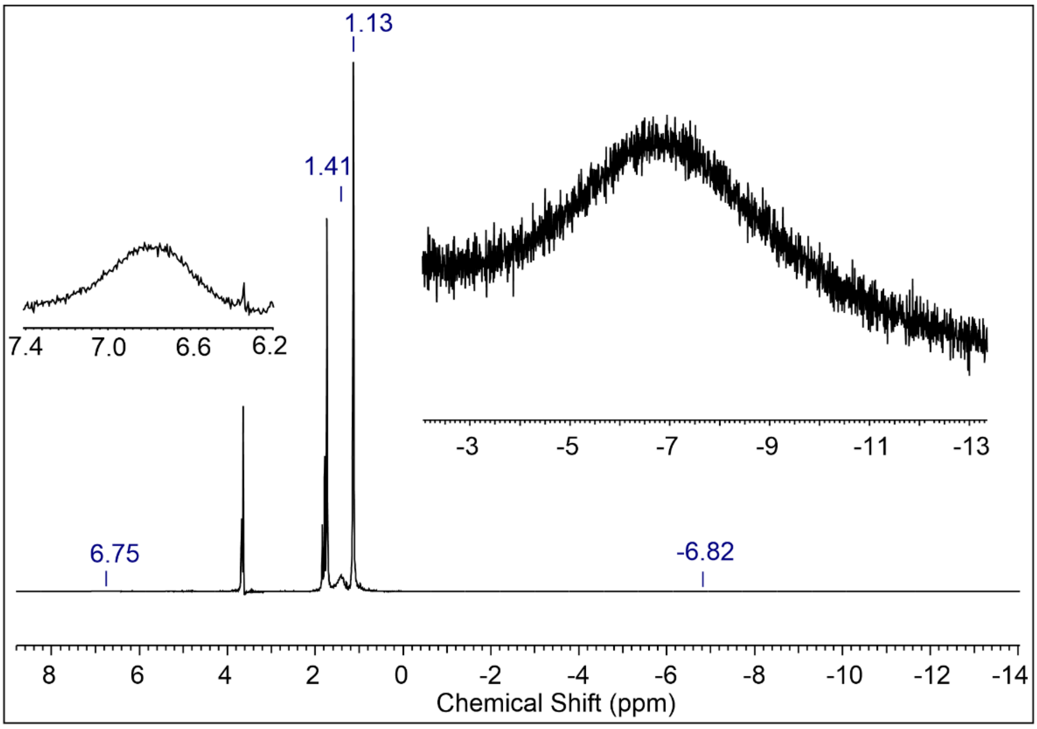

Figure S33. ^{1}H NMR spectrum (400.13 MHz, THF-d_8, 26 °C) of the reaction mixture of $[Ce\{OSi(OtBu)_3\}_3(thf)]_2(\mu_2\text{-}O_2C_6H_4)$ (**5^{hq}**) with two equivalents of $CoCp_2$.

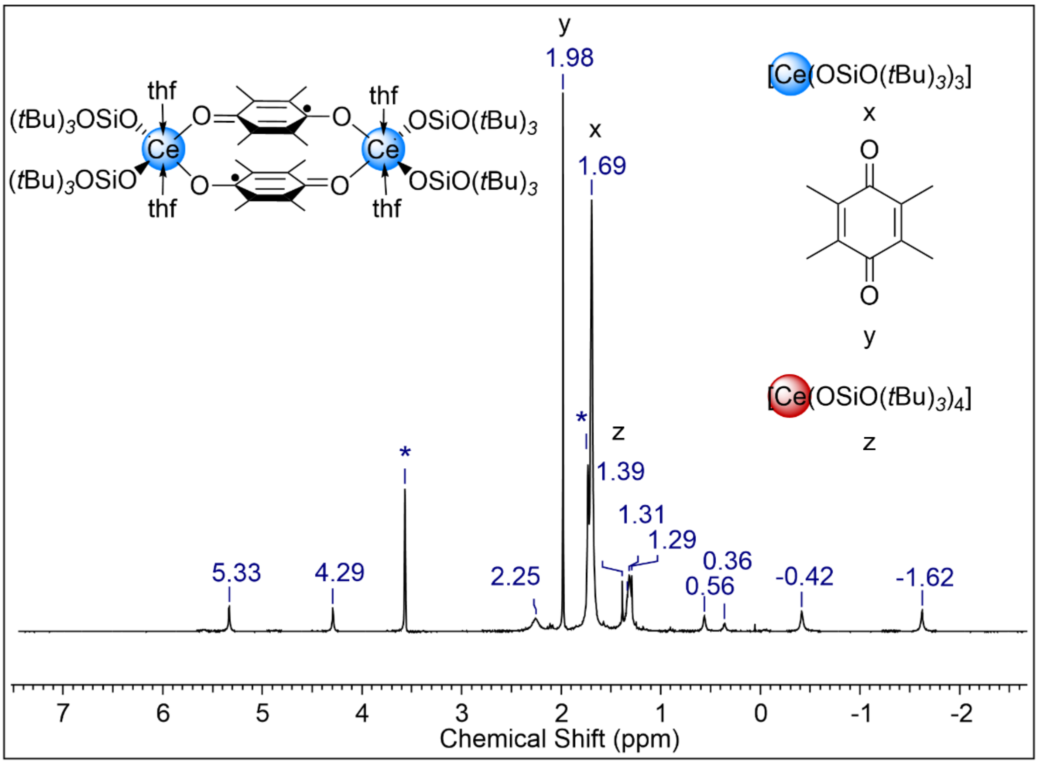

Figure S34. ^{1}H NMR spectrum (400.13 MHz, THF-d_8, 26 °C) of $[Ce\{OSi(OtBu)_3\}_2(thf)_2(\mu_2\text{-}O_2C_6Me_4)]_2$ (**8**). Signals assigned to starting material $[Ce\{OSi(OtBu)_3\}_3]_2$ and Me_4bq, and co-product $Ce[OSi(OtBu)_3]_4$ are marked with x, y and z.

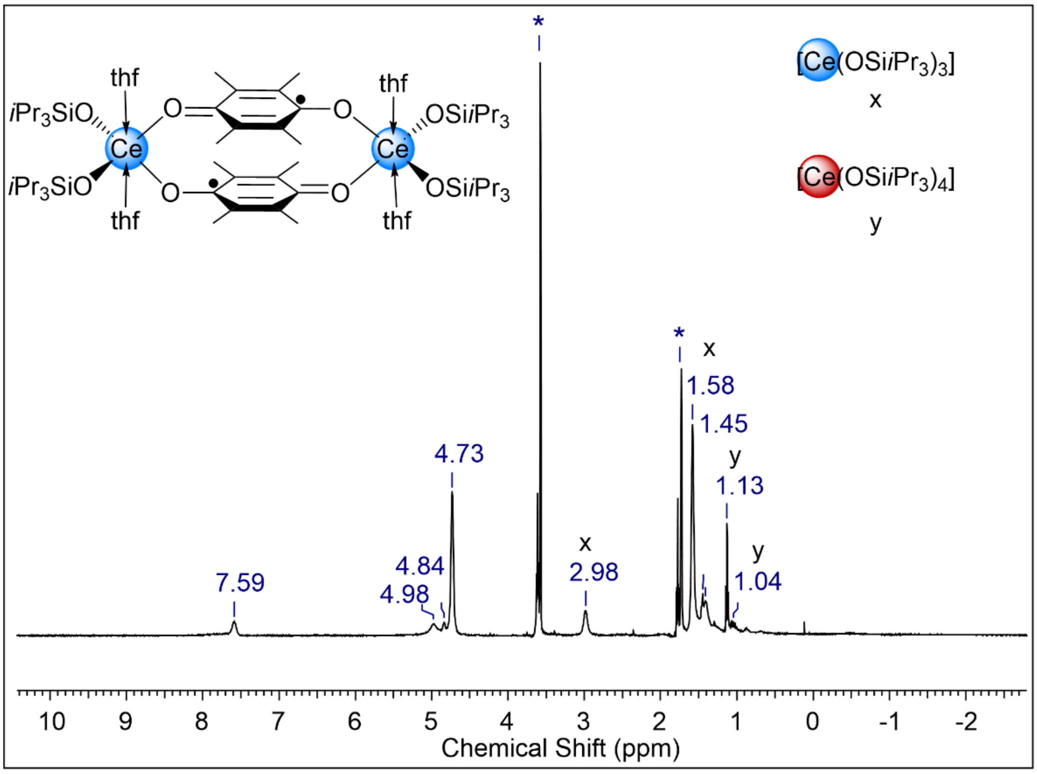

Figure S35. ^{1}H NMR spectrum (400.13 MHz, THF-d_8, 26 °C) of [Ce(OSi*i*Pr$_3$)$_2$(thf)$_2$(μ_2-O$_2$C$_6$Me$_4$)]$_2$ (**9**). Signals assigned to starting material [Ce(OSi*i*Pr$_3$)$_3$]$_2$ and co-product [Ce(OSi*i*Pr$_3$)$_4$] are marked with x and y.

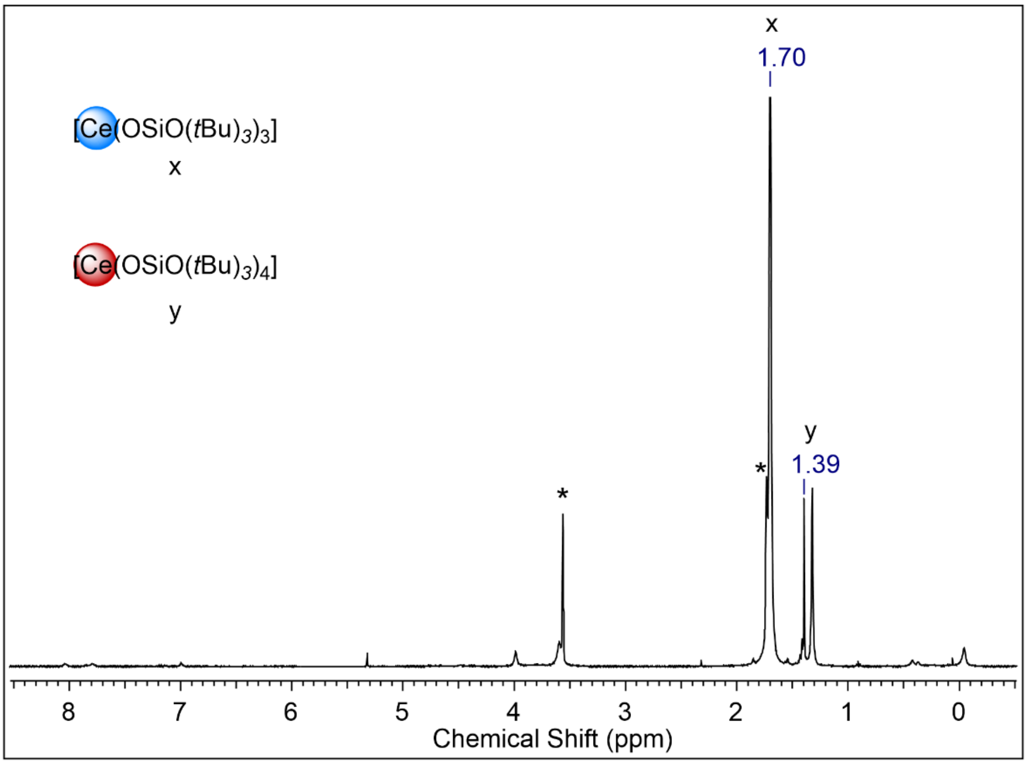

Figure S36. ^{1}H NMR spectrum (400.13 MHz, THF-d_8, 26 °C) of the reaction of [Ce(OSi(O*t*Bu)$_3$]$_2$ (**2**) with 1,4-naphthoquinone. Signals assigned to starting material **2** and co-product Ce[OSi(O*t*Bu)$_3$]$_4$ are marked with x and y.

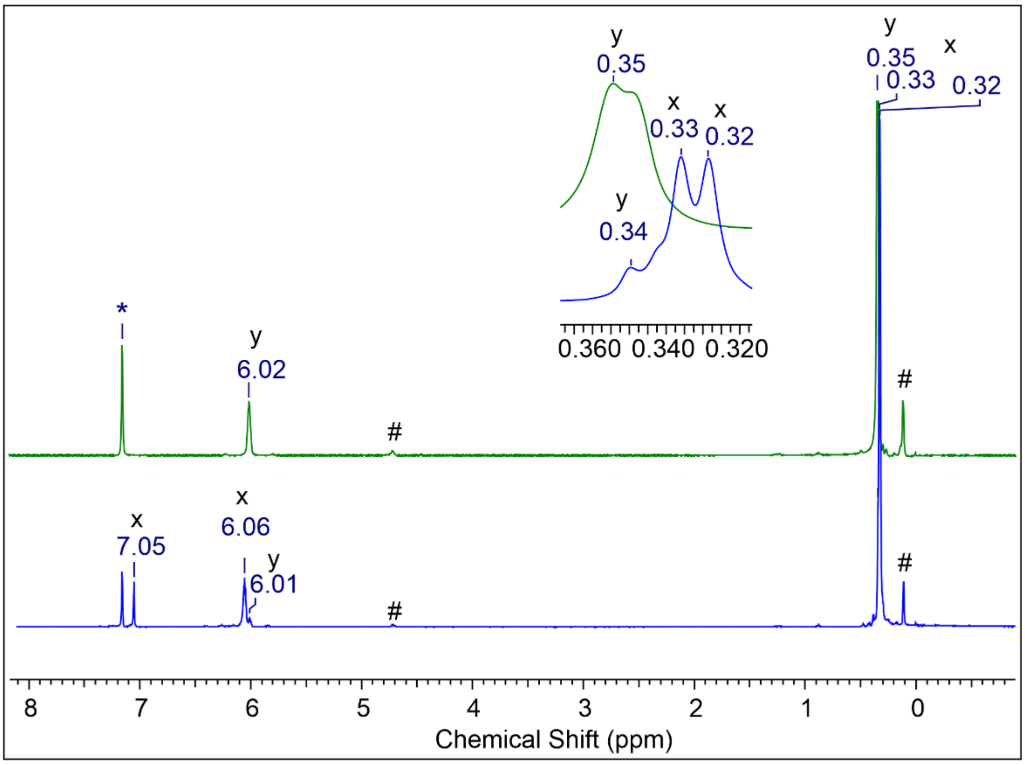

Figure S37. ^{1}H NMR spectrum (400.13 MHz, C_6D_6, 26 °C) of the reaction of [Ce{N(SiHMe$_2$)$_2$}$_3$]$_2$ with 1,4-benzoquinone recorded after 10 min (blue, bottom) and after 24 h (green, top). Signals assigned to putative [Ce{N(SiHMe$_2$)$_2$}$_3$]$_2$(μ_2-$O_2C_6H_4$) and co-product Ce[N(SiHMe$_2$)$_2$]$_4$ are marked with x and y.

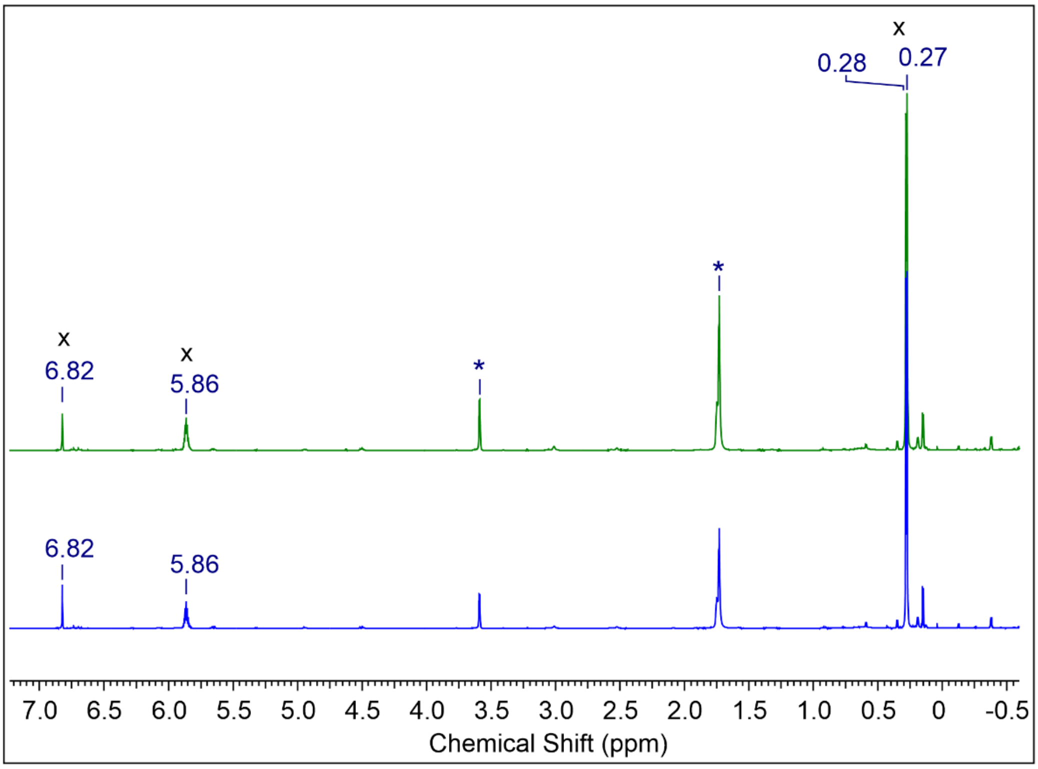

Figure S38. ^{1}H NMR spectrum (400.13 MHz, THF-d_8, 26 °C) of the reaction of [Ce{N(SiHMe$_2$)$_2$}$_3$]$_2$ with 1,4-benzoquinone recorded after 10 min (blue, bottom) and after 24 h (green, top). Signals assigned to putative [Ce{N(SiHMe$_2$)$_2$}$_3$]$_2$(μ_2-$O_2C_6H_4$) are marked with x.

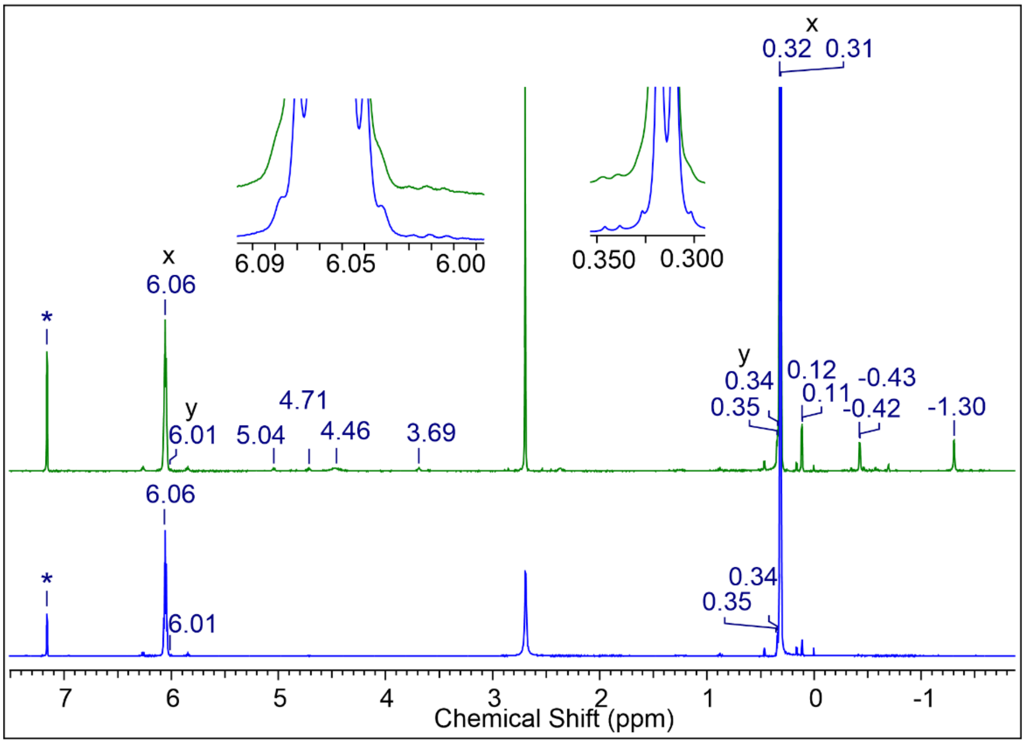

Figure S39. ^{1}H NMR spectrum (400.13 MHz, C_6D_6, 26 °C) of the reaction of $[Ce\{N(SiHMe_2)_2\}_3]_2$ with tetramethyl-1,4-benzoquinone recorded after 10 min (blue, bottom) and after 24 h (green, top). Signals assigned to putative $[Ce\{N(SiHMe_2)_2\}_3]_2(\mu_2\text{-}O_2C_6H_4)$ and co-product $Ce[N(SiHMe_2)_2]_4$ are marked with x and y.

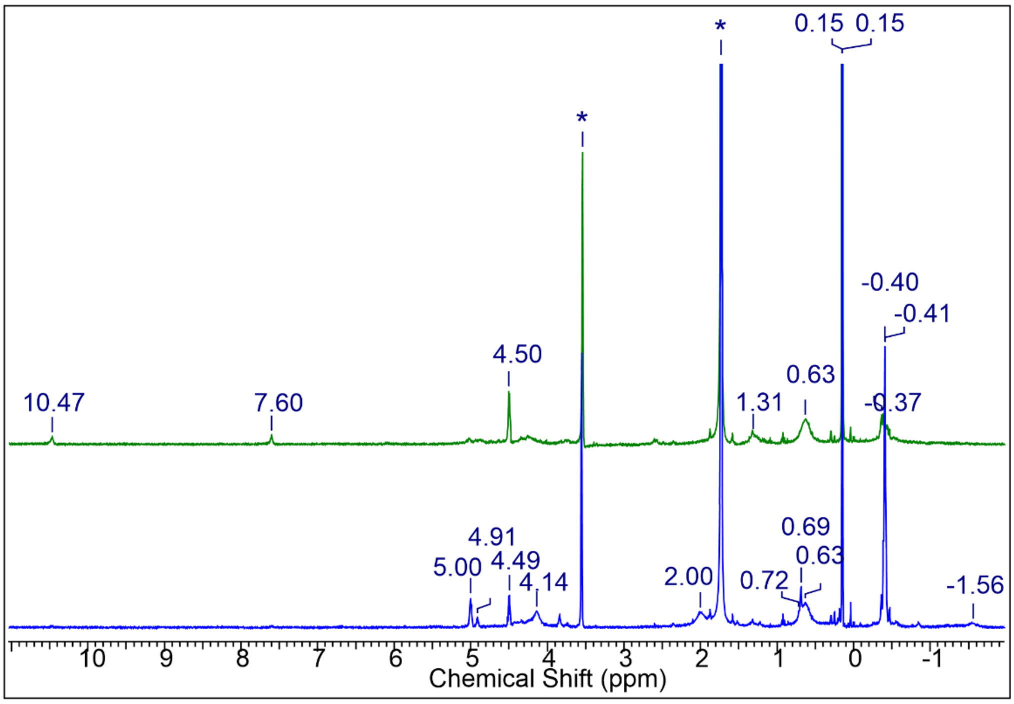

Figure S40. ^{1}H NMR spectrum (400.13 MHz, THF-d_8, 26 °C) of the reaction of $[Ce\{N(SiHMe_2)_2\}_3]_2$ with tetramethyl-1,4-benzoquinone recorded after 10 min (blue, bottom) and after 24 h (green, top).

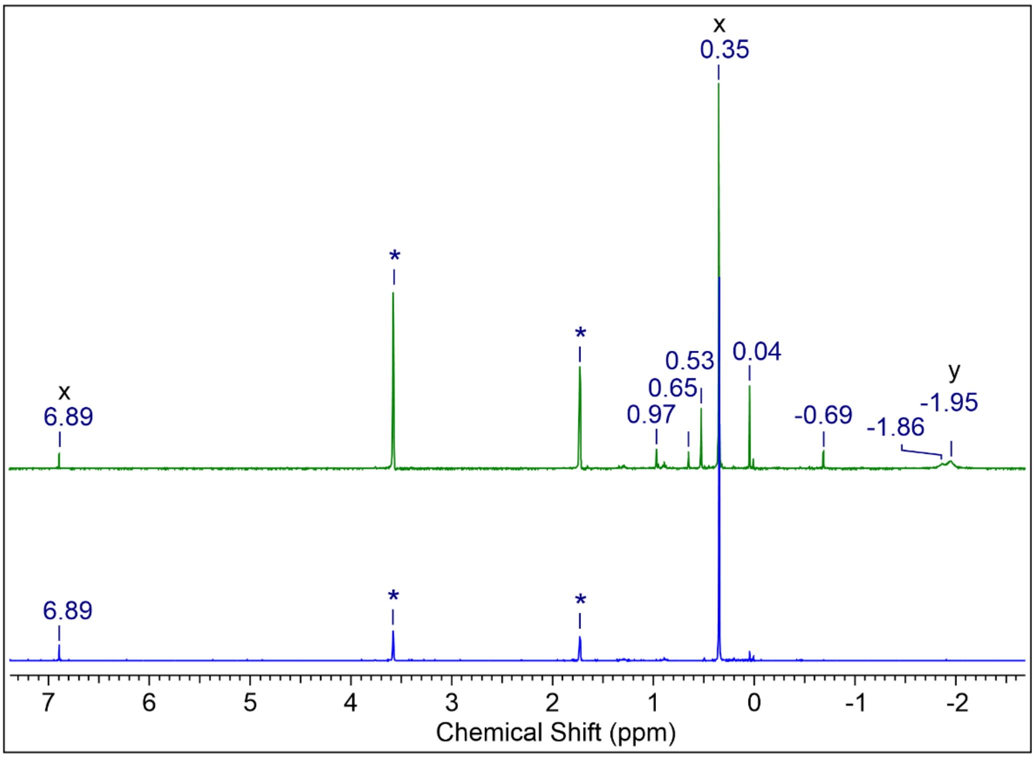

Figure S41. ^{1}H NMR spectrum (400.13 MHz, THF-d_8, 26 °C) of $\mathbf{4^{hq}}$ recorded after 10 min (blue, bottom) and after 24 h (green, top). Signals assigned to $\mathbf{4^{hq}}$ and co-product $Ce[N(SiMe_3)_2]_3$ are marked with x and y.

IR Spectra

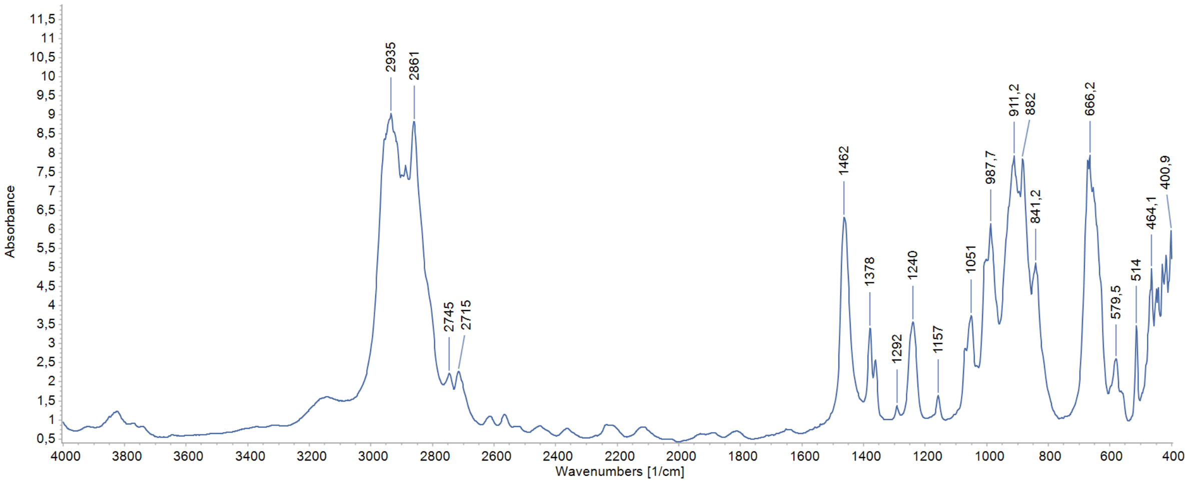

Figure S42. DRIFT spectrum of [Ce{(OSi*i*Pr$_3$)$_3$]$_2$ (**3**) at 25 °C.

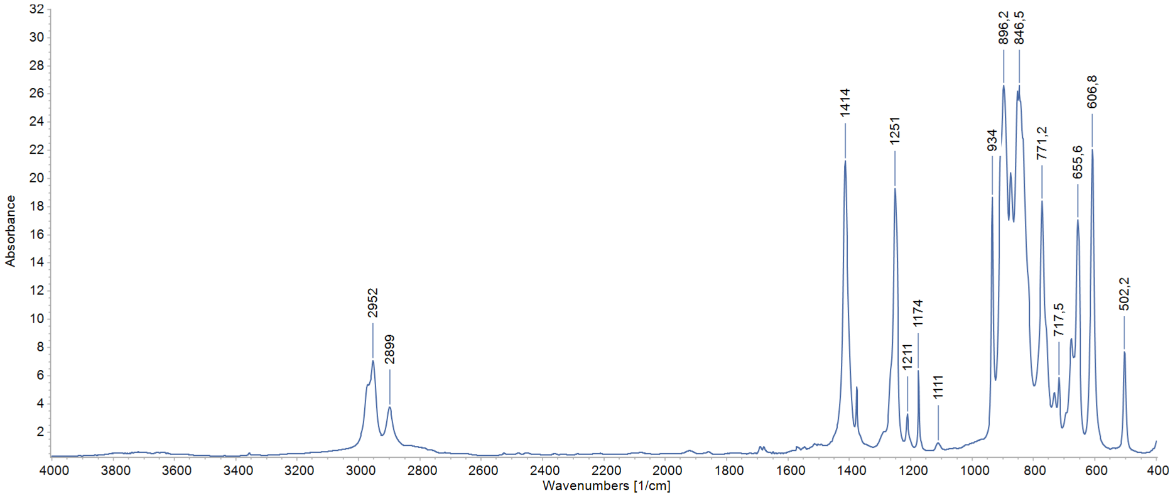

Figure S43. DRIFT spectrum of [Ce{N(SiMe$_3$)$_2$}$_3$]$_2$(μ_2-O$_2$C$_6$Cl$_4$) (**4^{Cl4hq}**) at 25 °C.

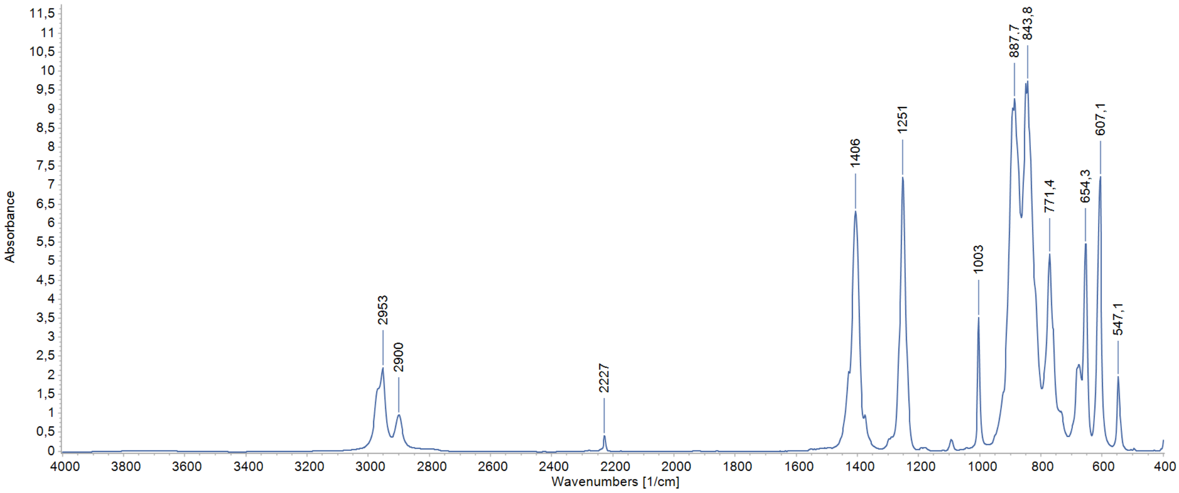

Figure S44. DRIFT spectrum of [Ce{N(SiMe$_3$)$_2$}$_3$]$_2$(μ_2-O$_2$C$_6$Cl$_2$(CN)$_2$) (**4^{ddhq}**) at 25 °C.

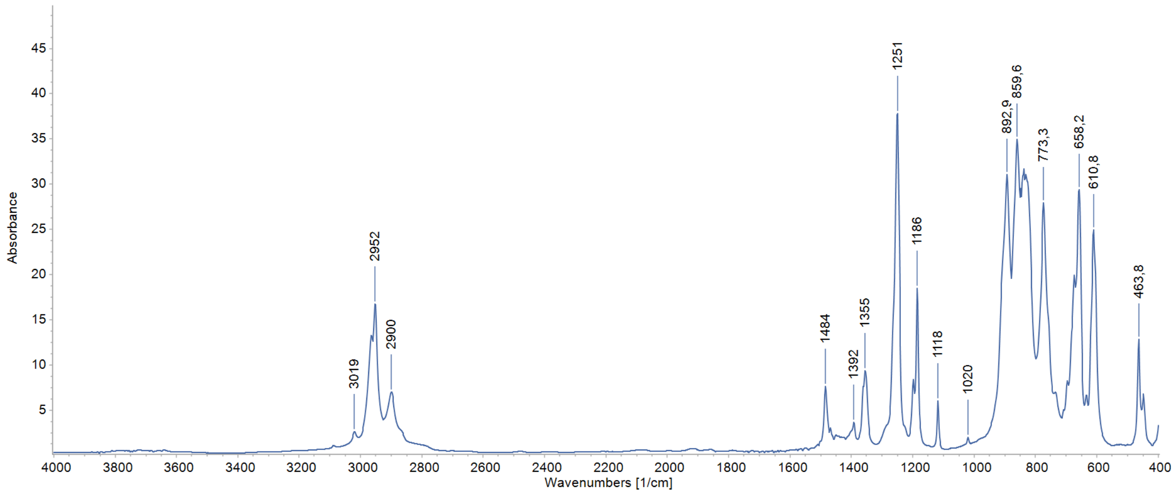

Figure S45. DRIFT spectrum of [Ce{N(SiMe$_3$)$_2$}$_3$]$_2$(μ_2-O$_2$C$_6$*t*Bu$_2$H$_2$) (**4**^{tBu2hq}) at 25 °C.

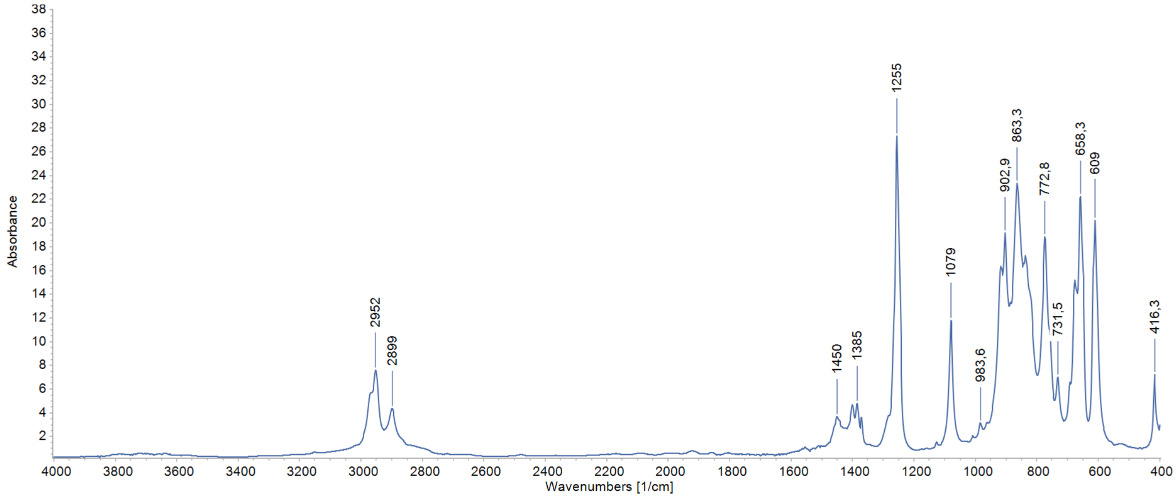

Figure S46. DRIFT spectrum of [Ce{N(SiMe$_3$)$_2$}$_3$]$_2$(μ_2-O$_2$C$_6$Me$_4$) (**4**^{Me4hq}) at 25 °C.

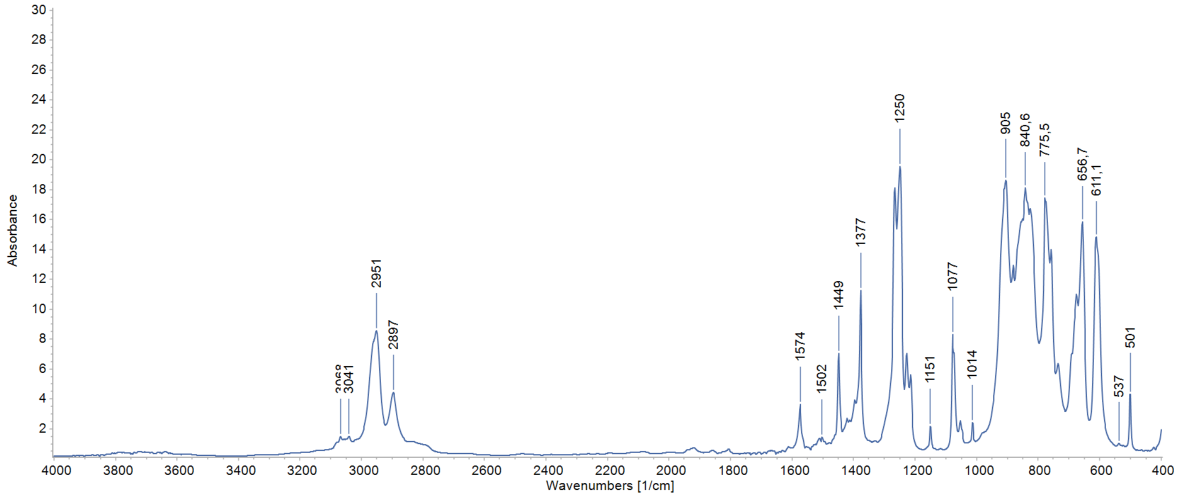

Figure S47. DRIFT spectrum of [Ce{N(SiMe$_3$)$_2$}$_3$]$_2$(μ_2-O$_2$C$_{10}$H$_6$) (**4**^{nq}) at 25 °C.

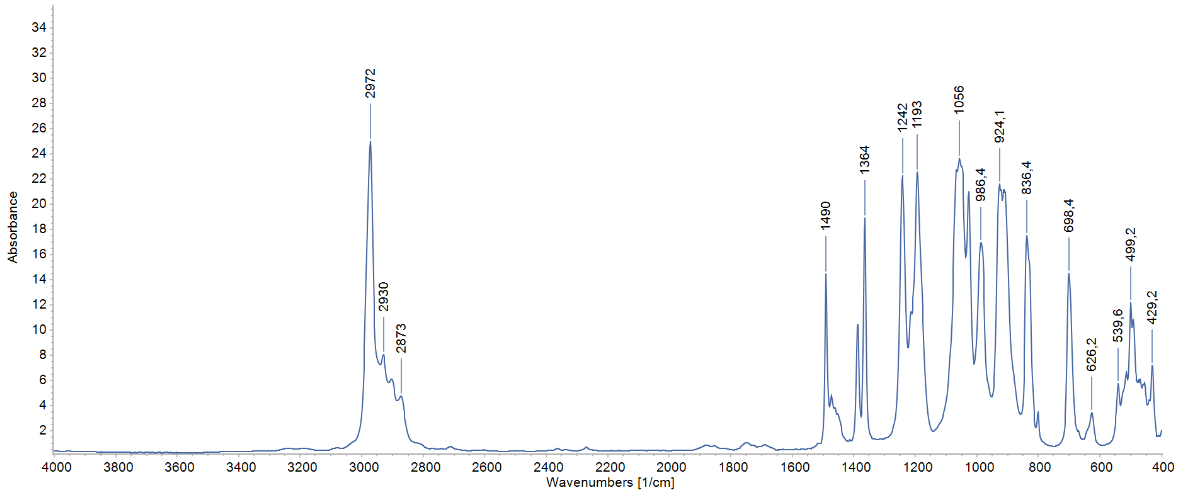

Figure S48. DRIFT spectrum of $[Ce\{OSi(O\mathit{t}Bu)_3\}_3(thf)]_2(\mu_2\text{-}O_2C_6H_4)$ ($\mathbf{5}^{\mathbf{hq}}$) at 25 °C.

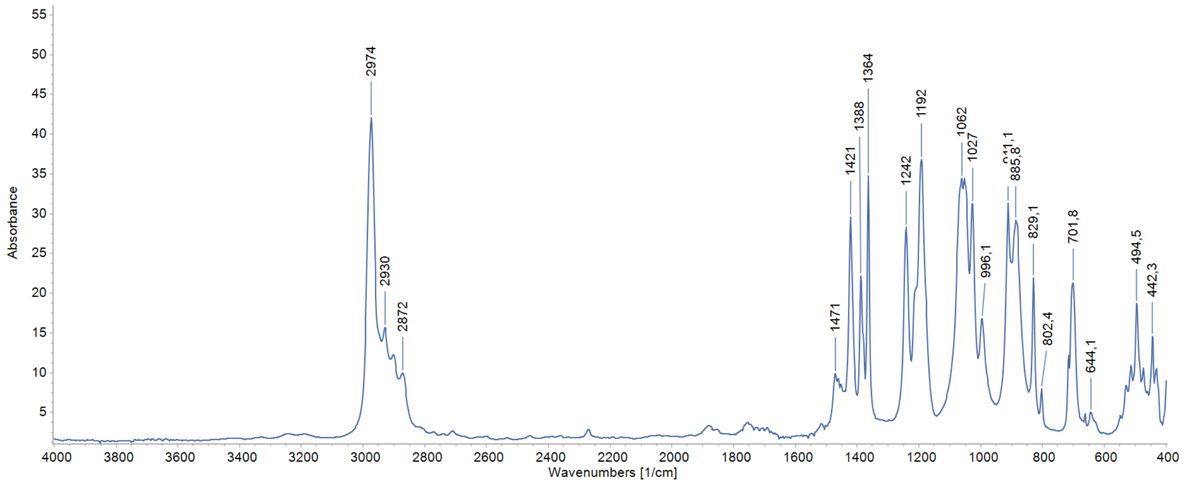

Figure S49. DRIFT spectrum of $[Ce\{OSi(O\mathit{t}Bu)_3\}_3(thf)]_2(\mu_2\text{-}O_2C_6Cl_4)$ ($\mathbf{5}^{\mathbf{Cl4hq}}$) at 25 °C.

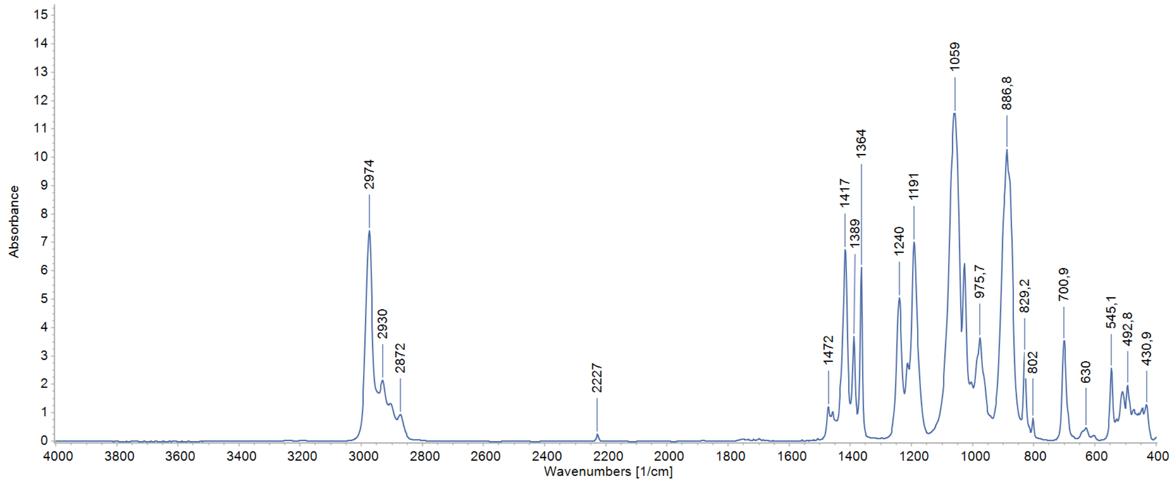

Figure S50. DRIFT spectrum of $[Ce\{OSi(O\mathit{t}Bu)_3\}_3(thf)]_2(\mu_2\text{-}O_2C_6Cl_2(CN)_2)$ ($\mathbf{5}^{\mathbf{ddhq}}$) at 25 °C.

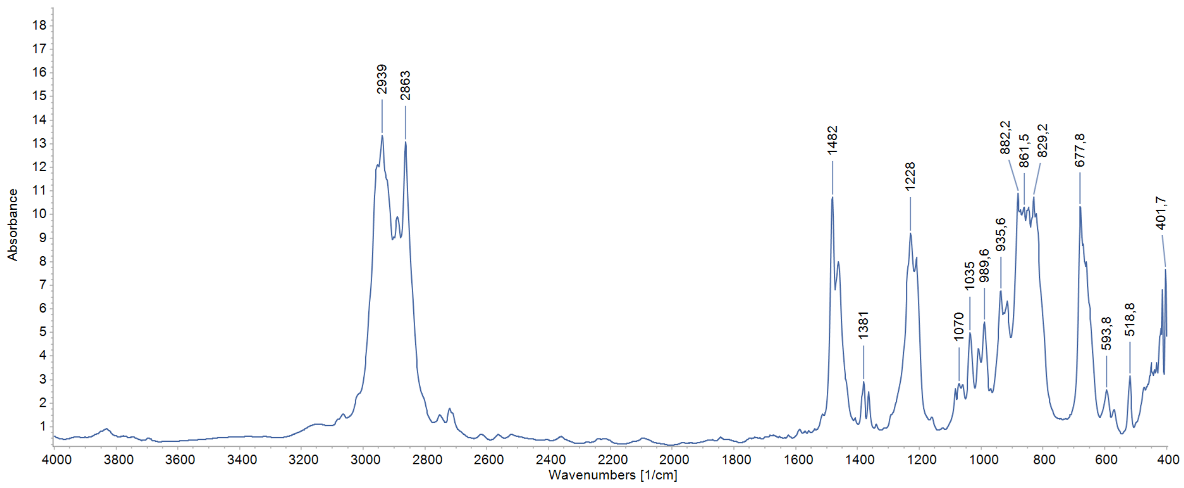

Figure S51. DRIFT spectrum of [Ce(OSi*i*Pr$_3$)$_3$(thf)]$_2$(μ_2-O$_2$C$_6$H$_4$) (**6**^{hq}) at 25 °C.

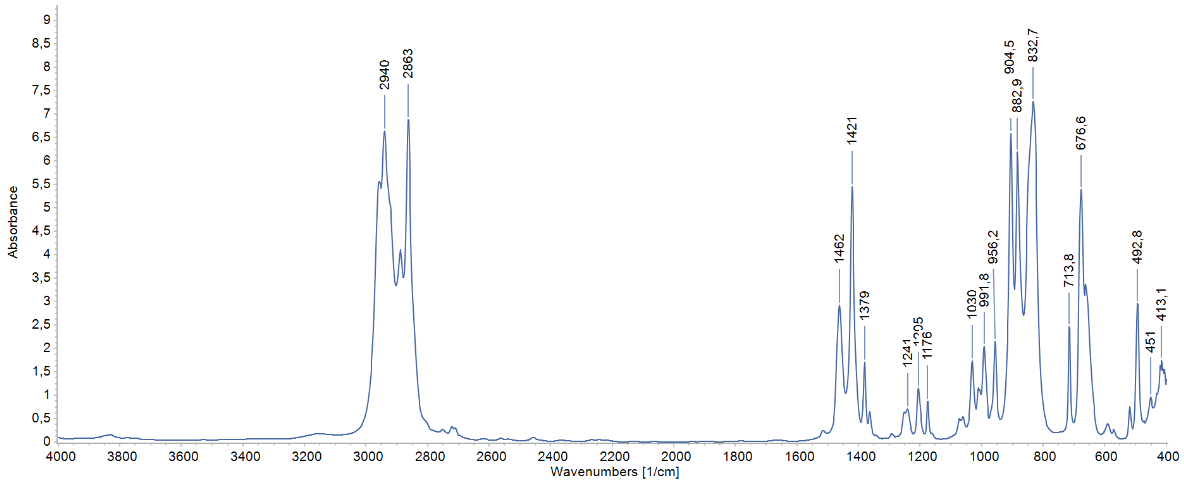

Figure S52. DRIFT spectrum of [Ce(OSi*i*Pr$_3$)$_3$(thf)]$_2$(μ_2-O$_2$C$_6$Cl$_4$) (**6**^{Cl4hq}) at 25 °C.

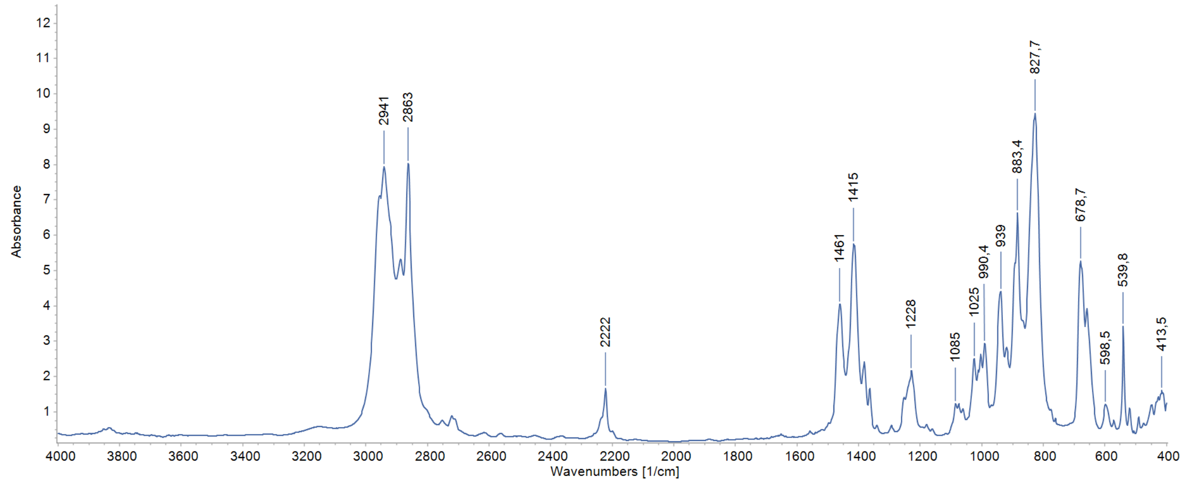

Figure S53. DRIFT spectrum of [Ce(OSi*i*Pr$_3$)$_3$(thf)]$_2$(μ_2-O$_2$C$_6$Cl$_2$(CN)$_2$) (**6**^{ddhq}) at 25 °C.

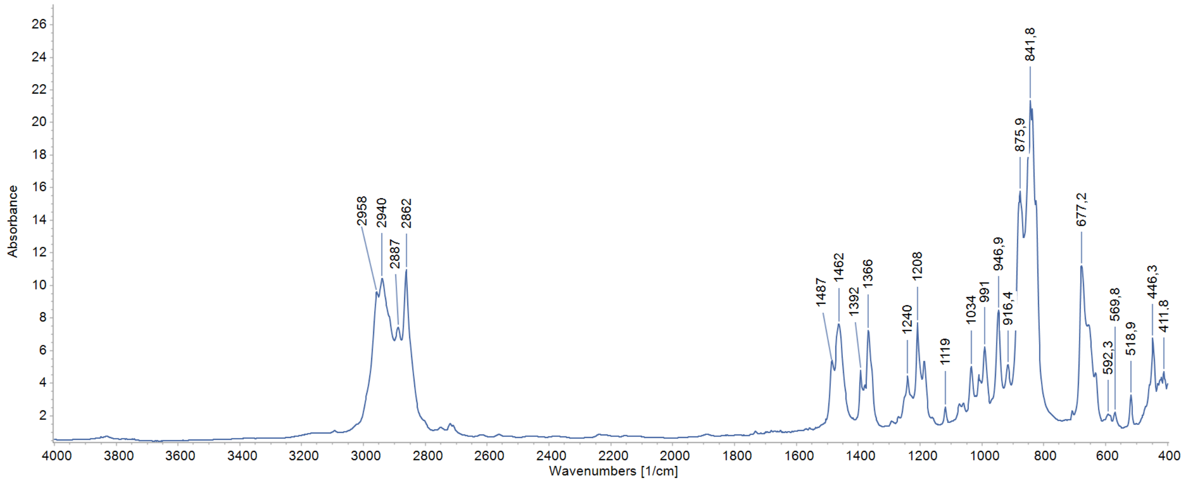

Figure S54. DRIFT spectrum of [Ce(OSi*i*Pr$_3$)$_3$(thf)]$_2$(μ_2-O$_2$C$_6$*t*Bu$_2$H$_2$) (**6**^{tBu2hq}) at 25 °C.

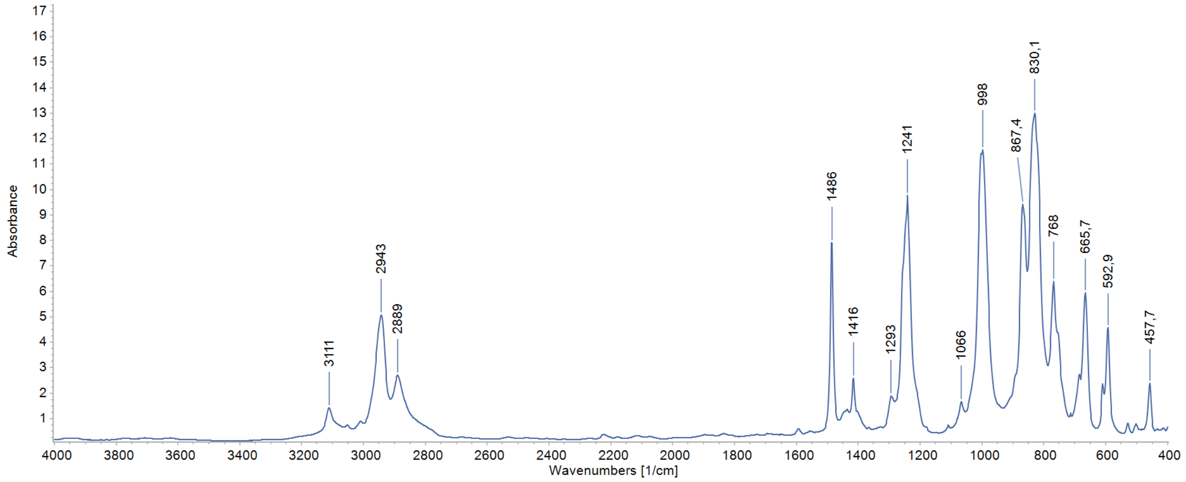

Figure S55. DRIFT spectrum of [(Ce{N(SiMe$_3$)$_2$}$_3$)$_2$(μ_2-O$_2$C$_6$H$_4$)][CoCp$_2$]$_2$ (**7**) at 25 °C.

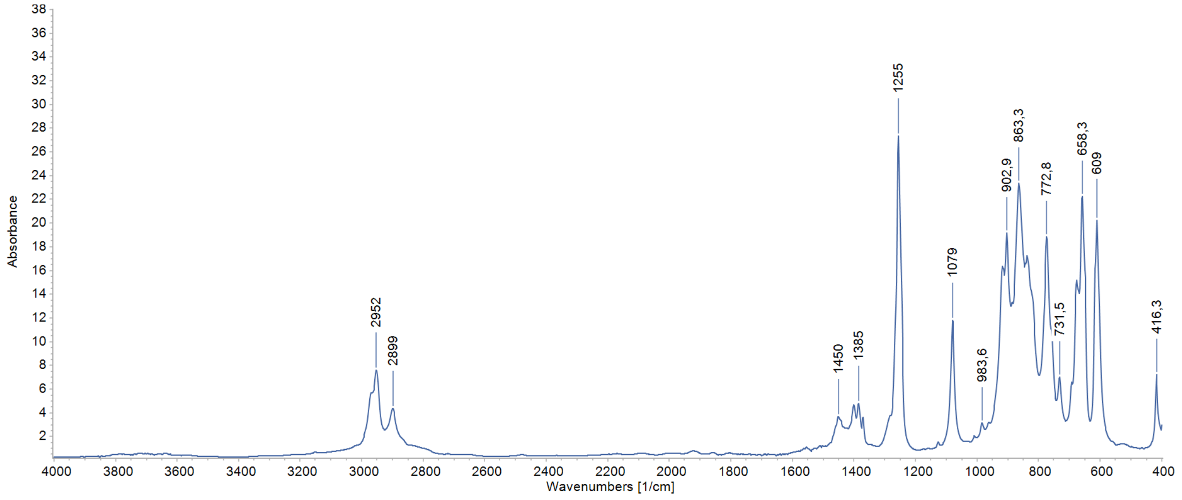

Figure S56. DRIFT spectrum of [Ce{OSi(O*t*Bu)$_3$}$_2$(thf)$_2$(μ_2-O$_2$C$_6$Me$_4$)]$_2$ (**8**) at 25 °C.

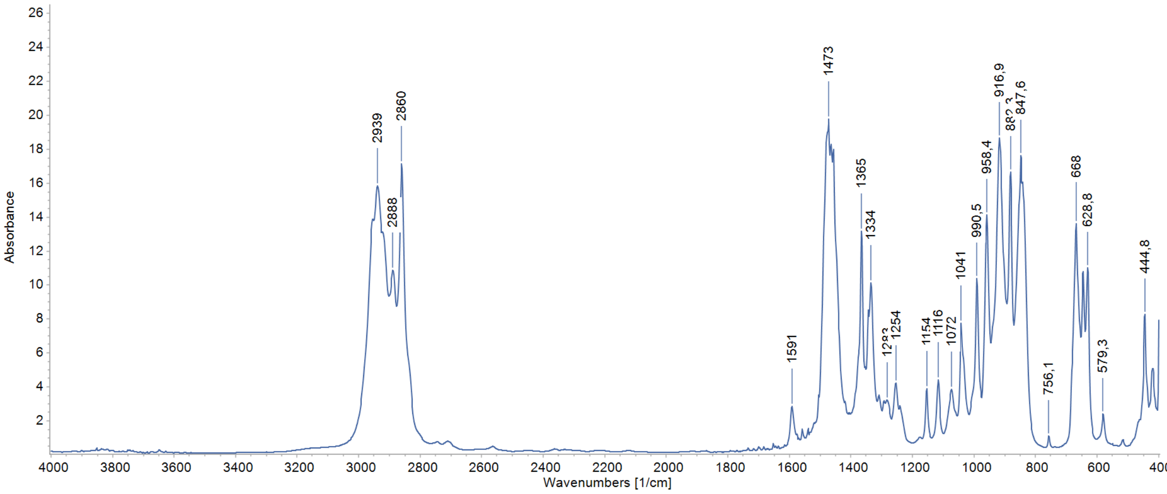

Figure S57. DRIFT spectrum of [(Ce(OSi*i*Pr$_3$)$_2$(thf)$_2$(μ_2-O$_2$C$_6$Me$_4$)]$_2$ (**9**) at 25 °C.

Cyclic voltammetry measurements

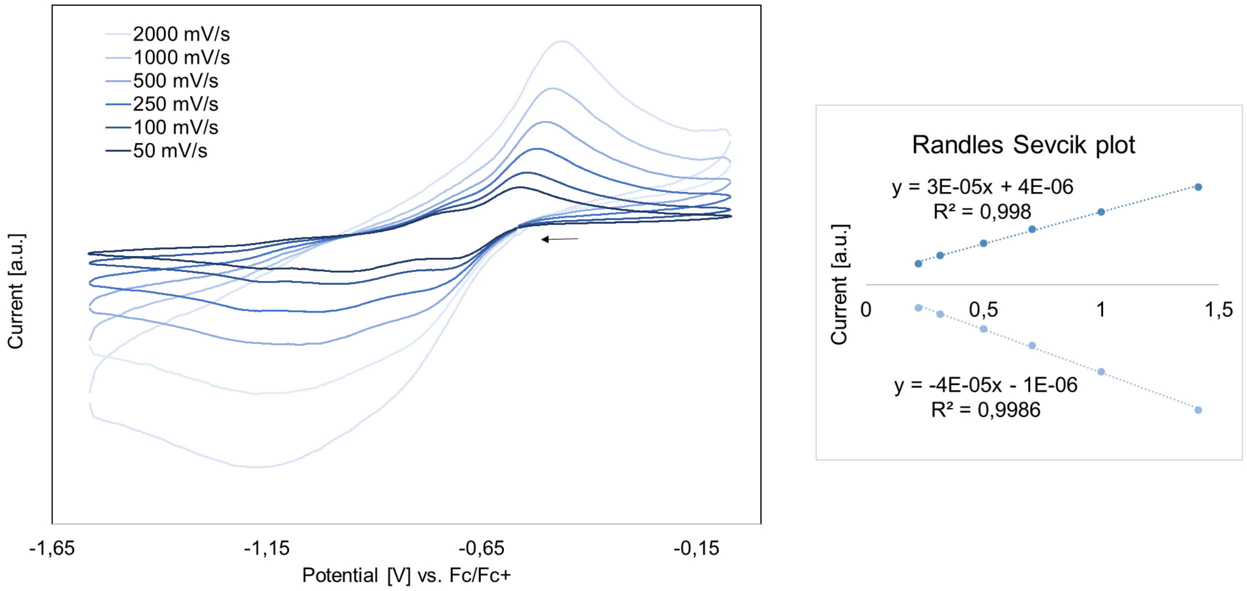

Figure S58. left: Cerium(III/IV) redox couple of complex **4**^{hq} in THF at ambient temperature and varying scan rates; right: corresponding Randles Sevcik plot of the anodic (top) and cathodic (bottom) redox features.

Table S1. Electrochemical data for the redox couples vs Fc/Fc$^+$ of complex **4**^{hq} in THF at ambient temperature.

scan rate	E_{1pa} [V]	E_{2pa} [V]	E_{1pc} [V]	E_{2pc} [V]	$\Delta E_{2pa/2pc}$ [V]	i_{pa}/i_{pc}
50 mV/s	-	−0.558	−0.699	−0.966	0.408	0.92
100 mV/s	-	−0.544	−0.728	−0.999	0.455	0.98
250 mV/s	-	−0.521	−0.781	−1.036	0.515	0.93
500 mV/s	-	−0.503	−0.794	−1.078	0.575	0.91
1000 mV/s	-	−0.486	−0.815	−1.182	0.696	0.84
2000 mV/s	-	−0.463	−0.835	−1.186	0.723	0.78

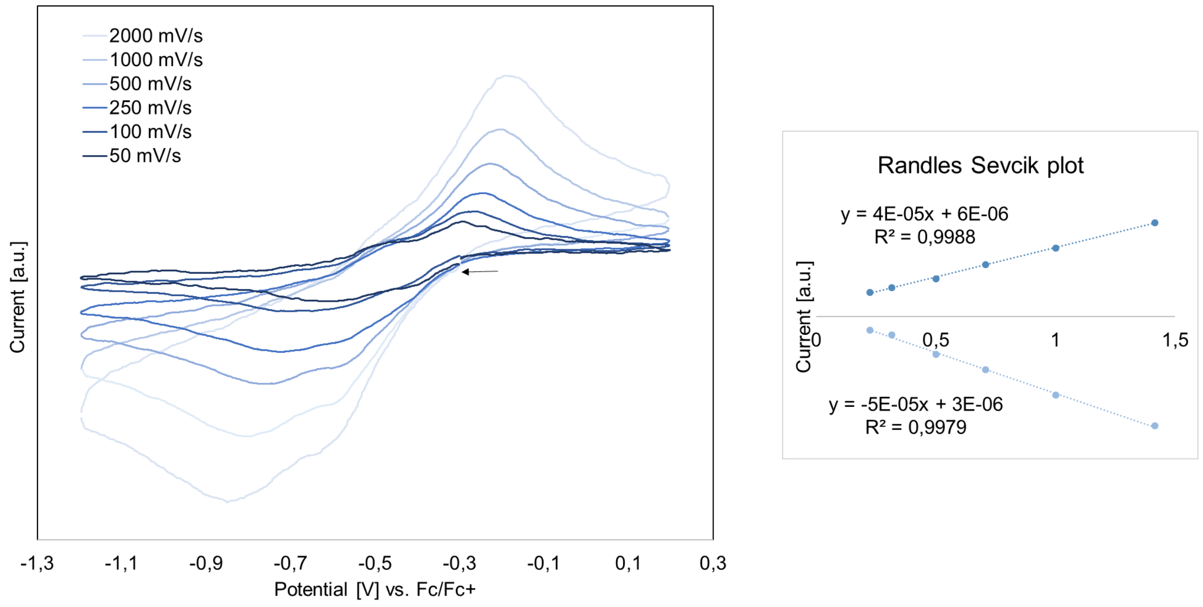

Figure S59a. left: Cerium(III/IV) redox couple of complex $\mathbf{4}^{\mathbf{Cl4hq}}$ in THF at ambient temperature and varying scan rates; right: corresponding Randles Sevcik plot of the anodic (top) and cathodic (bottom) redox features.

Table S2a. Electrochemical data for the redox couples vs Fc/Fc^+ of complex $\mathbf{4}^{\mathbf{Cl4hq}}$ in THF at ambient temperature.

scan rate	E_{1pa} [V]	E_{2pa} [V]	E_{1pc} [V]	E_{2pc} [V]	$\Delta E_{2pa/2pc}$ [V]	i_{pa}/i_{pc}
50 mV/s	-	−0.290	−0.415	−0.624	0.334	0.90
100 mV/s	-	−0.260	−0.437	−0.728	0.468	0.91
250 mV/s	-	−0.247	−0.576	−0.726	0.479	0.70
500 mV/s	-	−0.228	−0.582	−0.771	0.543	0.76
1000 mV/s	-	−0.205	−0.597	−0.805	0.600	0.74
2000 mV/s	-	−0.194	−0.626	−0.849	0.655	0.77

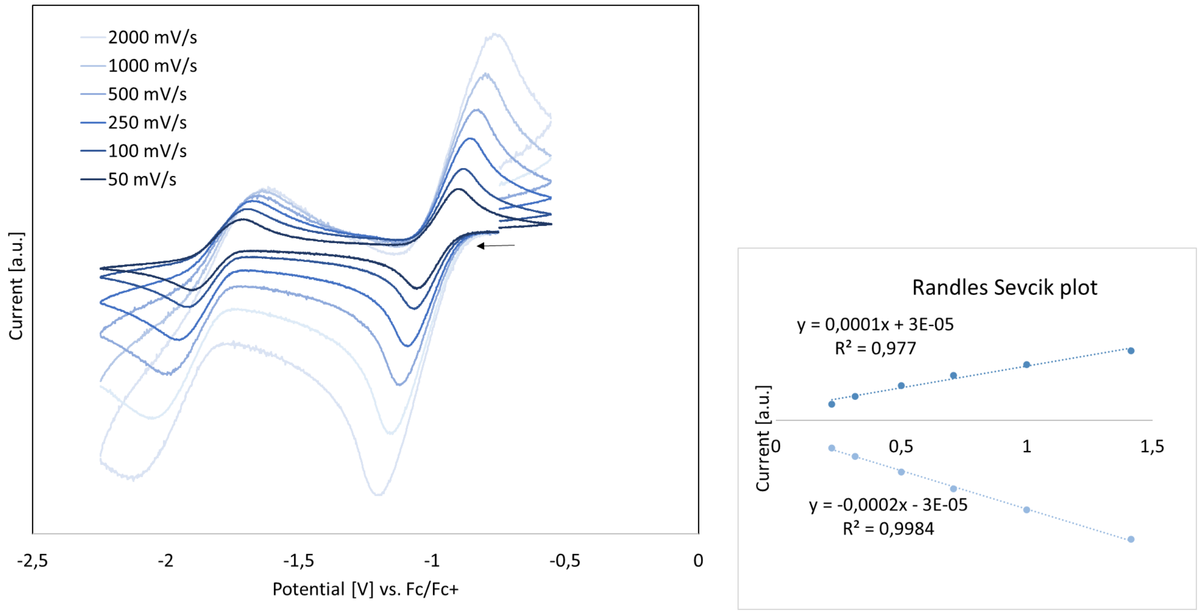

Figure 59b. left: redox couple of tetrachloro-1,4-benzoquinone in THF at ambient temperature and varying scan rates; right: corresponding Randles Sevcik plot of the anodic (top) and cathodic (bottom) redox features.

Table S2b. Electrochemical data for the redox couples vs Fc/Fc^+ of complex tetrachloro-1,4-benzoquinone in THF at ambient temperature.

scan rate	E_{1pa} [V]	E_{2pa} [V]	E_{1pc} [V]	E_{2pc} [V]	$\Delta E_{2pa/2pc}$ [V]	i_{pa}/i_{pc}
50 mV/s	−0.4785	−1.304	−0.6735	−1.499	0.208	0.21
100 mV/s	−0.4645	−1.29	−0.6815	−1.507	0.234	0.30
250 mV/s	−0.4505	−1.276	−0.7195	−1.545	0.287	0.29
500 mV/s	−0.4185	−1.244	−0.7515	−1.577	0.332	0.26
1000 mV/s	−0.4085	−1.234	−0.8195	−1.645	0.409	0.22
2000 mV/s	−0.3855	−1.211	−0.8925	−1.718	0.526	0.18

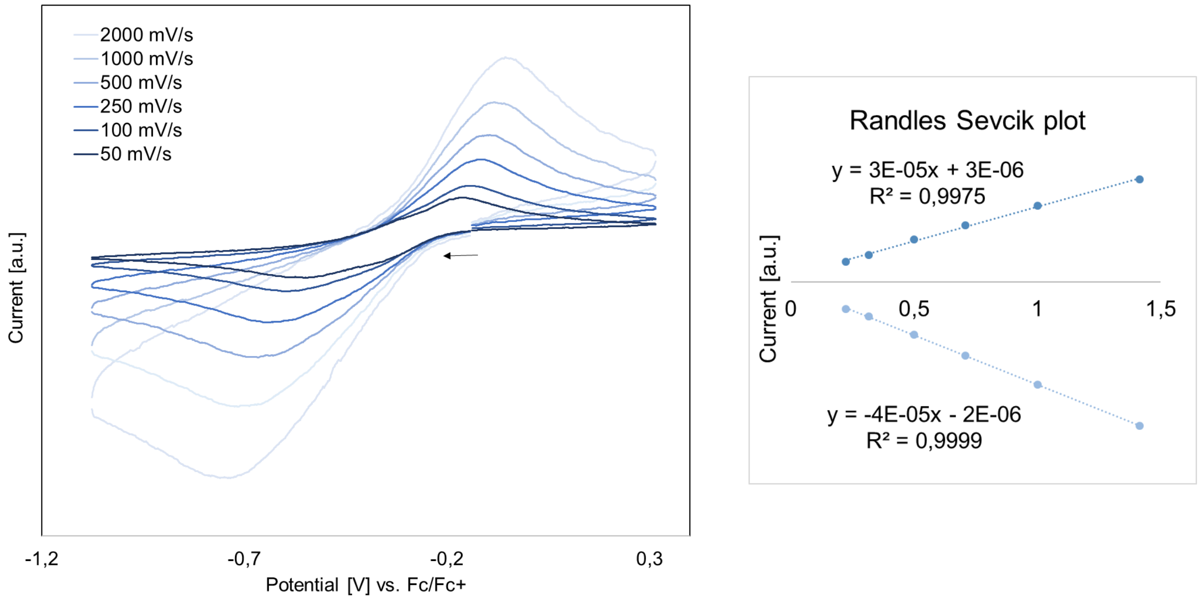

Figure S60. left: Cerium(III/IV) redox couple of complex **4**$^{\mathbf{ddhq}}$ in THF at ambient temperature and varying scan rates; right: corresponding Randles Sevcik plot of the anodic (top) and cathodic (bottom) redox features.

Table S3. Electrochemical data for the redox couples vs Fc/Fc$^+$ of complex **4**$^{\mathbf{ddhq}}$ in THF at ambient temperature.

scan rate	E_{1pa} [V]	E_{2pa} [V]	E_{1pc} [V]	E_{2pc} [V]	$\Delta E_{2pa/2pc}$ [V]	i_{pa}/i_{pc}
50 mV/s	-	−0.1685	-	−0.5455	0.377	0.78
100 mV/s	-	−0.1405	-	−0.5965	0.456	0.80
250 mV/s	-	−0.1165	-	−0.6495	0.533	0.82
500 mV/s	-	−0.1005	-	−0.6655	0.565	0.78
1000 mV/s	-	−0.0835	-	−0.7155	0.632	0.75
2000 mV/s	-	−0.0555	-	−0.7355	0.680	0.72

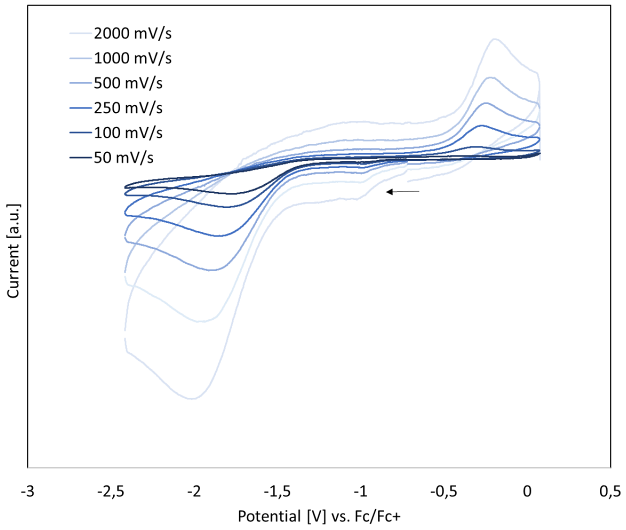

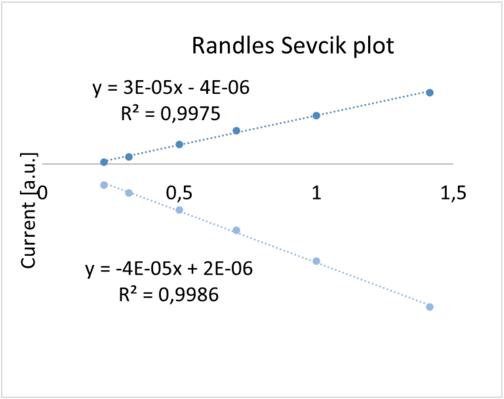

Figure S61. left: Cerium(III/IV) redox couple of complex $\mathbf{5^{hq}}$ in THF at ambient temperature and varying scan rates; right: corresponding Randles Sevcik plot of the anodic (top) and cathodic (bottom) redox features.

Table S4. Electrochemical data for the redox couples vs Fc/Fc$^+$ of complex $\mathbf{5^{hq}}$ in THF at ambient temperature.

scan rate	E_{1pa} [V]	E_{2pa} [V]	E_{1pc} [V]	E_{2pc} [V]	$\Delta E_{2pa/2pc}$ [V]	i_{pa}/i_{pc}
50 mV/s	-	−0.3625	-	−1.7785	1.416	0.11
100 mV/s	-	−0.3095	-	−1.8105	1.501	0.27
250 mV/s	-	−0.2725	-	−1.8425	1.57	0.44
500 mV/s	-	−0.2495	-	−1.9125	1.663	0.51
1000 mV/s	-	−0.2255	-	−1.9795	1.754	0.51
2000 mV/s	-	−0.1925	-	−2.0145	1.822	0.50

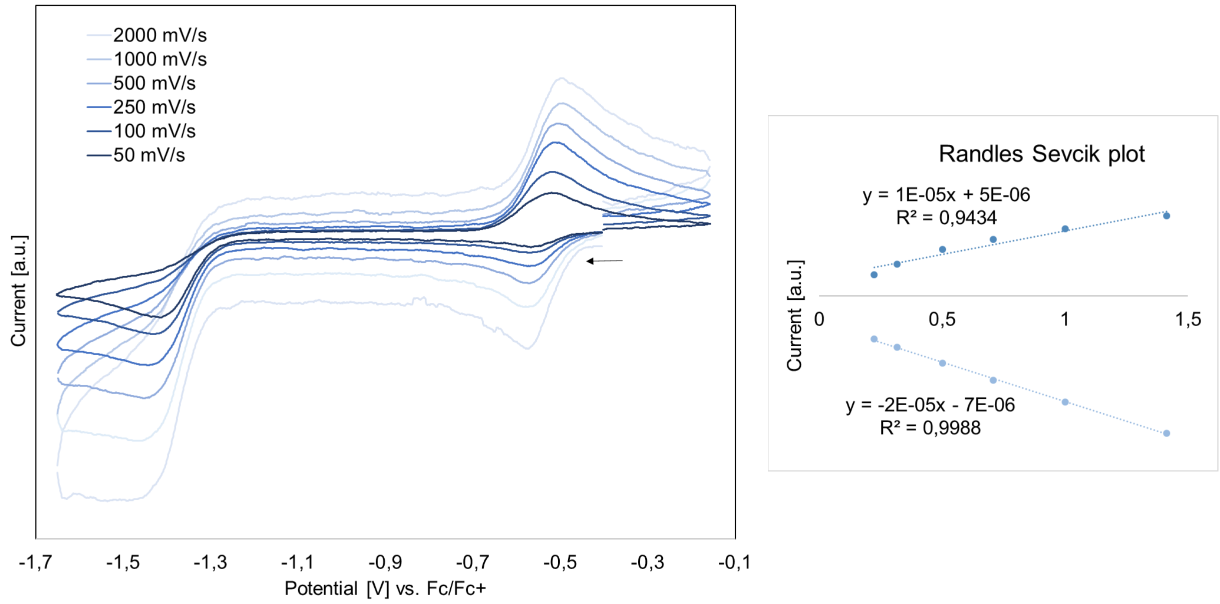

Figure S62. left: Cerium(III/IV) redox couple of complex **5**$^{\mathbf{Cl4hq}}$ in THF at ambient temperature and varying scan rates; right: corresponding Randles Sevcik plot of the anodic (top) and cathodic (bottom) redox features. The reductive feature at ~ −0.5 V results of a minor impurity of the sample.

Table S5. Electrochemical data for the redox couples vs Fc/Fc$^+$ of complex **5**$^{\mathbf{Cl4hq}}$ in THF at ambient temperature.

scan rate	E_{1pa} [V]	E_{2pa} [V]	E_{1pc} [V]	E_{2pc} [V]	$\Delta E_{2pa/2pc}$ [V]	i_{pa}/i_{pc}
50 mV/s	-	−0.5205	-	−1.4135	0.893	0.49
100 mV/s	-	−0.5175	-	−1.4335	0.916	0.63
250 mV/s	-	−0.5155	-	−1.4435	0.928	0.70
500 mV/s	-	−0.5075	-	−1.4535	0.946	0.67
1000 mV/s	-	−0.4935	-	−1.4715	0.978	0.64
2000 mV/s	-	−0.4975	-	−1.5355	1.038	0.59

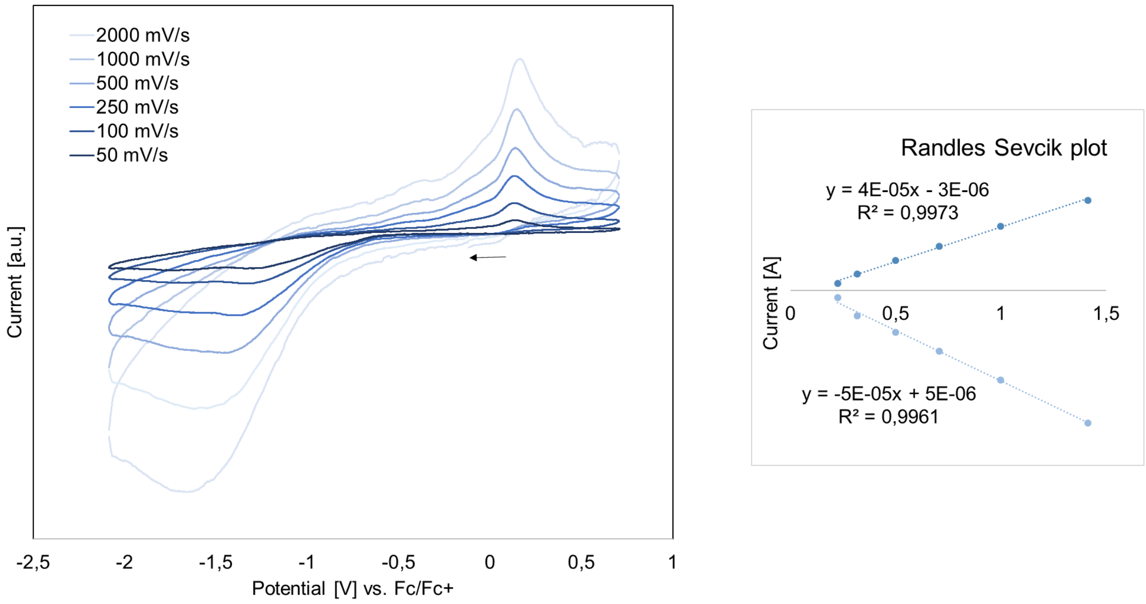

Figure S63. left: Cerium(III/IV) redox couple of complex **5**^{ddhq} in THF at ambient temperature and varying scan rates; right: corresponding Randles Sevcik plot of the anodic (top) and cathodic (bottom) redox features.

Table S6. Electrochemical data for the redox couples vs Fc/Fc^{+} of complex **5**^{ddhq} in THF at ambient temperature.

scan rate	E_{1pa} [V]	E_{2pa} [V]	E_{1pc} [V]	E_{2pc} [V]	$\Delta E_{2pa/2pc}$ [V]	i_{pa}/i_{pc}
50 mV/s	-	0.130	-	−1.353	1.483	0.95
100 mV/s	-	0.131	-	−1.312	1.443	0.63
250 mV/s	-	0.138	-	−1.394	1.532	0.72
500 mV/s	-	0.138	-	−1.434	1.572	0.73
1000 mV/s	-	0.150	-	−1.570	1.720	0.72
2000 mV/s	-	0.166	-	−1.646	1.812	0.68

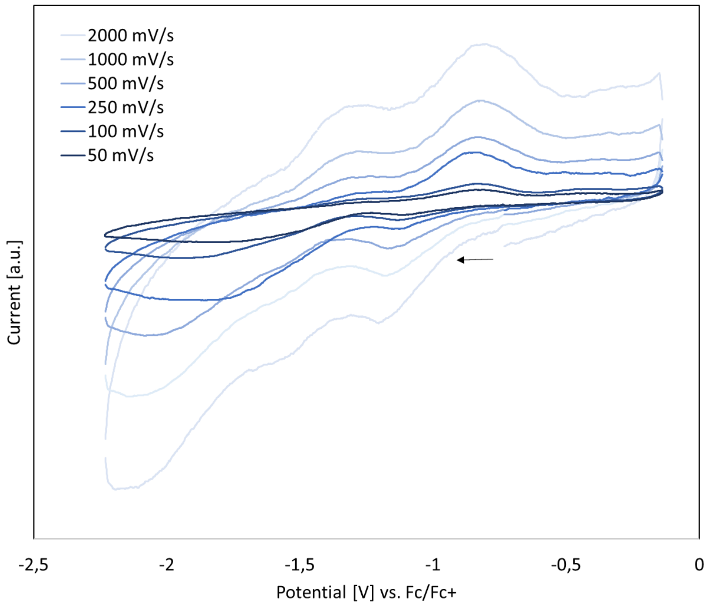

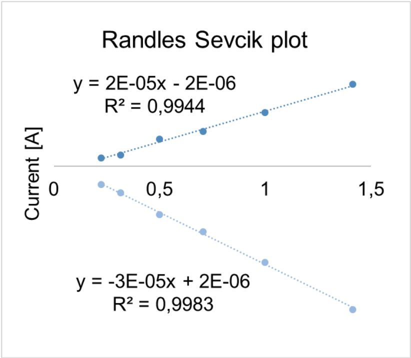

Figure S64. left: Cerium(III/IV) redox couple of complex **6^{hq}** in THF at ambient temperature and varying scan rates; right: corresponding Randles Sevcik plot of the anodic (top) and cathodic (bottom) redox features.

Table S7. Electrochemical data for the redox couples vs Fc/Fc^{+} of complex **6^{hq}** in THF at ambient temperature.

scan rate	E_{1pa} [V]	E_{2pa} [V]	E_{1pc} [V]	E_{2pc} [V]	$\Delta E_{2pa/2pc}$ [V]	i_{pa}/i_{pc}
50 mV/s	−1.273	−0.817	−1.116	−1.816	0.999	0.43
100 mV/s	−1.274	−0.835	−1.131	−1.969	1.134	0.42
250 mV/s	−1.292	−0.840	−1.146	−2.075	1.021	0.55
500 mV/s	−1.300	−0.825	−1.167	−2.081	1.256	0.53
1000 mV/s	−1.306	−0.810	−1.172	−2.145	1.335	0.55
2000 mV/s	−1.312	−0.800	−1.204	−2.193	1.393	0.57

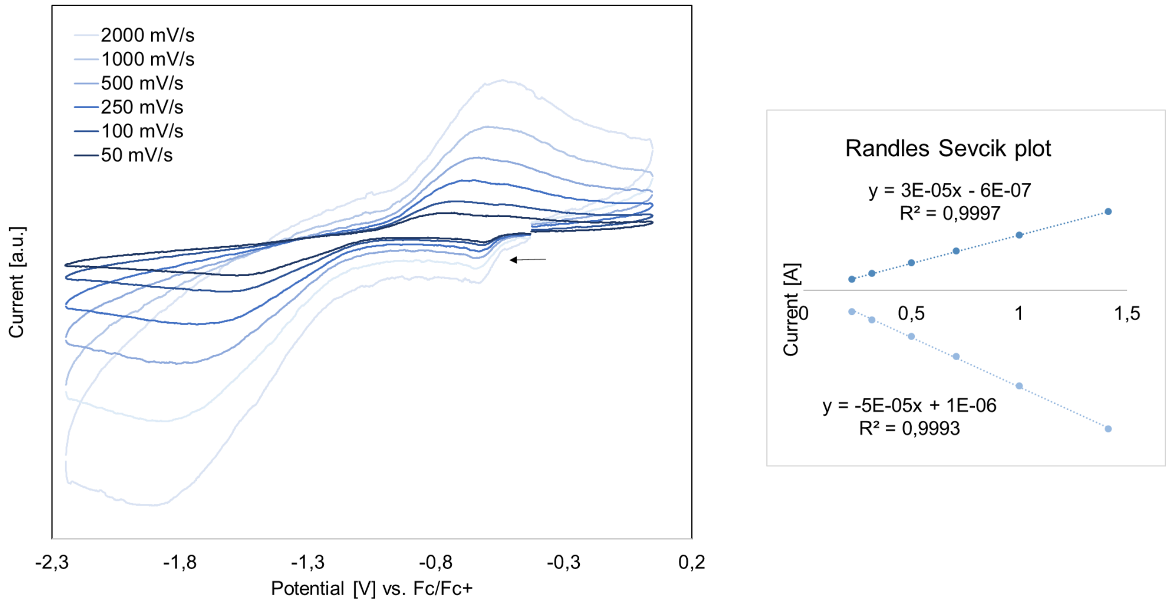

Figure S65. left: Cerium(III/IV) redox couple of complex **6^Cl4hq** in THF at ambient temperature and varying scan rates; right: corresponding Randles Sevcik plot of the anodic (top) and cathodic (bottom) redox features. The reductive feature at ~ −0.5 V results of a minor impurity of the sample.

Table S8. Electrochemical data for the redox couples vs Fc/Fc^+ of complex **6^Cl4hq** in THF at ambient temperature.

scan rate	E_{1pa} [V]	E_{2pa} [V]	E_{1pc} [V]	E_{2pc} [V]	$\Delta E_{2pa/2pc}$ [V]	i_{pa}/i_{pc}
50 mV/s	-	−0.729	-	−1.580	0.851	0.52
100 mV/s	-	−0.715	-	−1.640	0.925	0.57
250 mV/s	-	−0.654	-	−1.736	1.082	0.60
500 mV/s	-	−0.633	-	−1.816	1.183	0.59
1000 mV/s	-	−0.597	-	−1.880	1.283	0.57
2000 mV/s	-	−0.542	-	−1.905	1.363	0.57

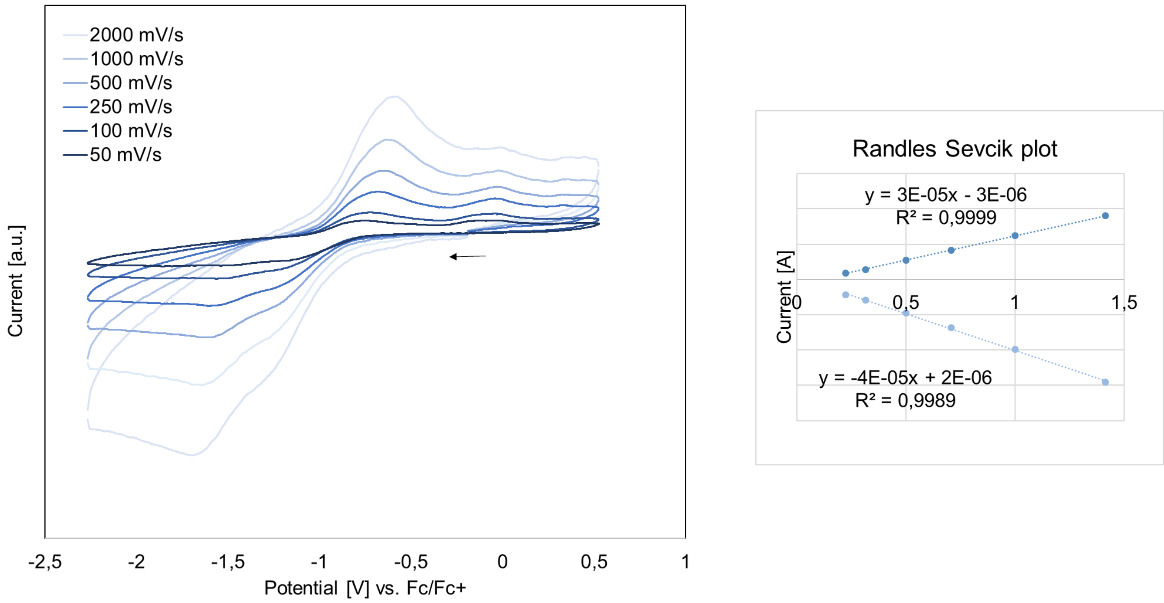

Figure S66. left: Cerium(III/IV) redox couple of complex **6**^{ddhq} in THF at ambient temperature and varying scan rates; right: corresponding Randles Sevcik plot of the anodic (top) and cathodic (bottom) redox features.

Table S9. Electrochemical data for the redox couples vs Fc/Fc$^+$ of complex **6**^{ddhq} in THF at ambient temperature.

scan rate	E_{1pa} [V]	E_{2pa} [V]	E_{1pc} [V]	E_{2pc} [V]	$\Delta E_{2pa/2pc}$ [V]	i_{pa}/i_{pc}
50 mV/s	-	−0.751	−1.149	−1.484	0.733	0.40
100 mV/s	-	−0.721	−1.186	−1.565	0.844	0.48
250 mV/s	-	−0.682	−1.210	−1.609	0.927	0.58
500 mV/s	-	−0.665	−1.232	−1.619	0.954	0.60
1000 mV/s	-	−0.624	−1.252	−1.649	1.025	0.62
2000 mV/s	-	−0.592	−1.259	−1.695	1.103	0.62

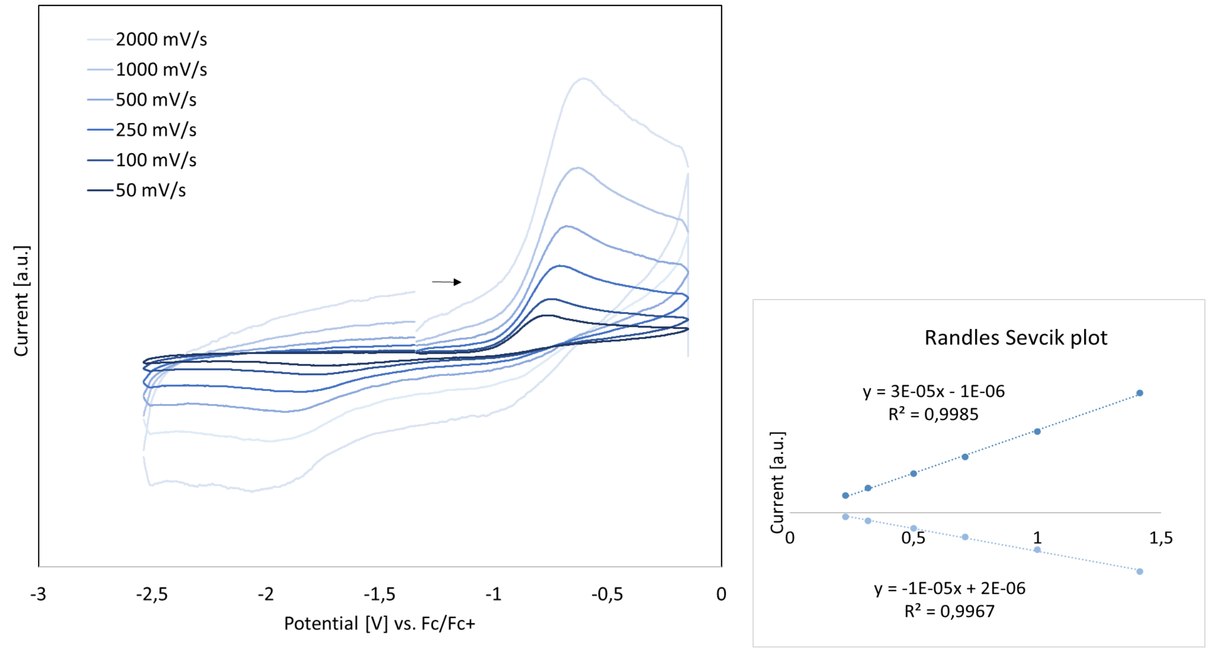

Figure S67. left: Cerium(III/IV) redox couple of complex **3** in THF at ambient temperature and varying scan rates; right: corresponding Randles Sevcik plot of the anodic (top) and cathodic (bottom) redox features.

Table S10. Electrochemical data for the redox couples vs Fc/Fc$^+$ of complex **3** in THF at ambient temperature.

scan rate	E_{pa} [V]	E_{pc} [V]	$\Delta E_{pa/pc}$ [V]	i_{pa}/i_{pc}
50 mV/s	−0.605	−1.757	1.152	0.232
100 mV/s	−0.633	−1.799	1.166	0.315
250 mV/s	−0.680	−1.850	1.170	0.395
500 mV/s	−0.712	−1.919	1.207	0.428
1000 mV/s	−0.746	−1.972	1.226	0.451
2000 mV/s	−0.778	−2.061	1.283	0.487

UV/Vis spectra

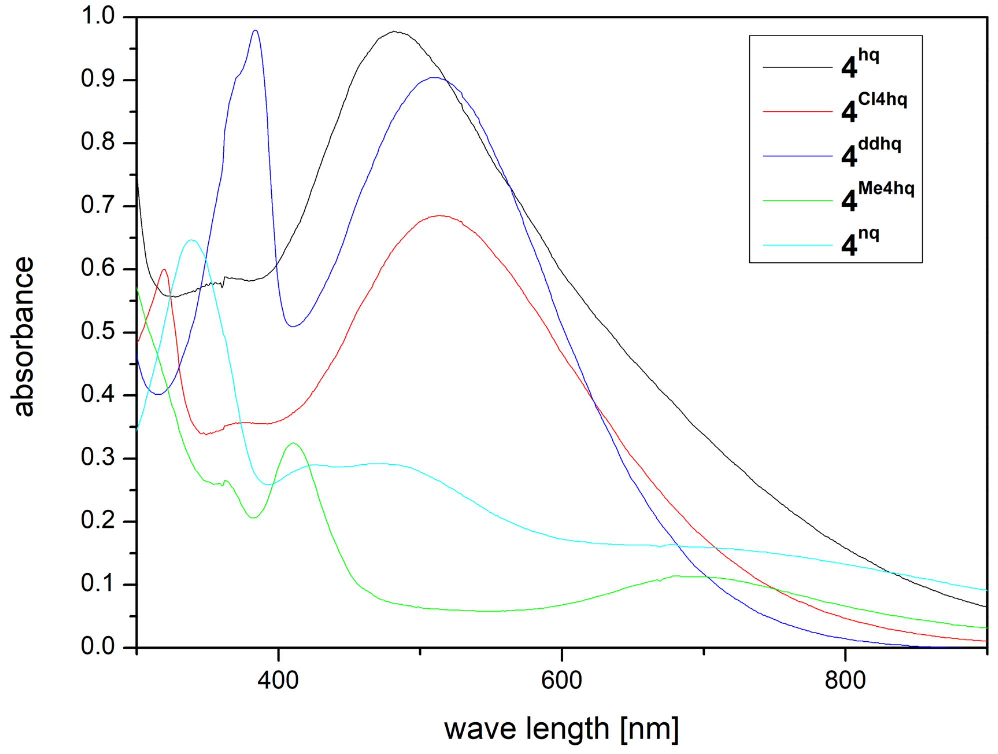

Figure S68. UV/Vis spectra of **4**^{hq}, **4**^{Cl4hq}, **4**^{ddhq}, **4**^{Me4hq} and **4**^{nhq} in toluene at ambient temperature. The strong absorption band of **4**^{nhq} at 339 nm may refer to 1,4-naphthoquinone.[8]

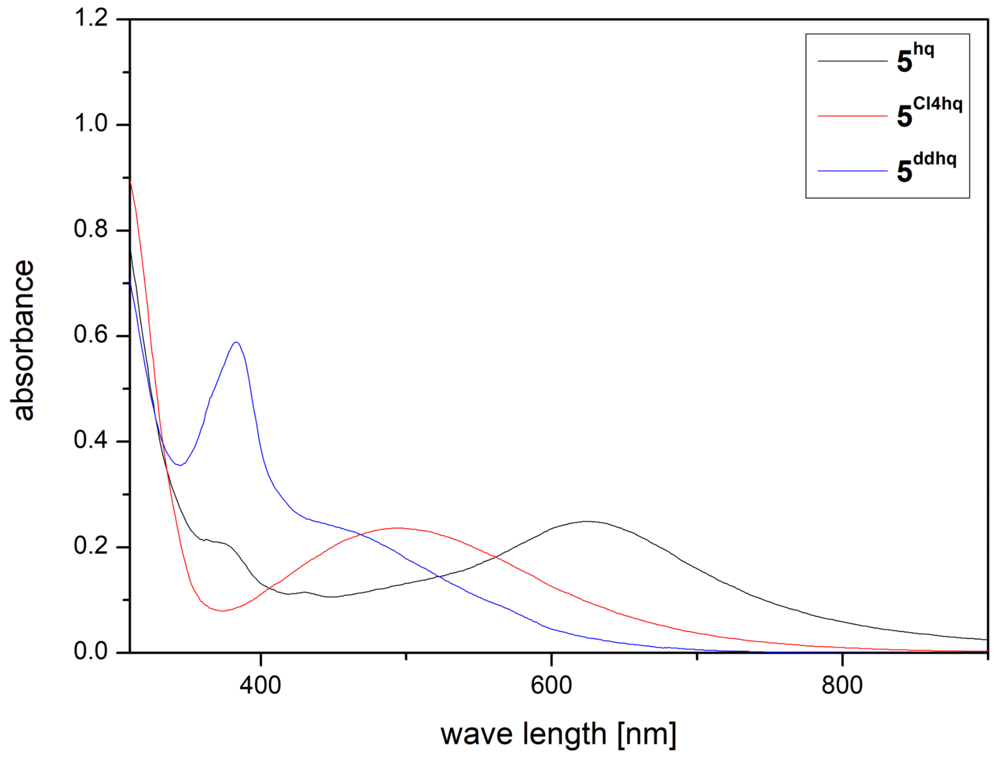

Figure S69. UV/Vis spectra of **5**^{hq}, **5**^{Cl4hq} and **5**^{ddhq} in toluene at ambient temperature.

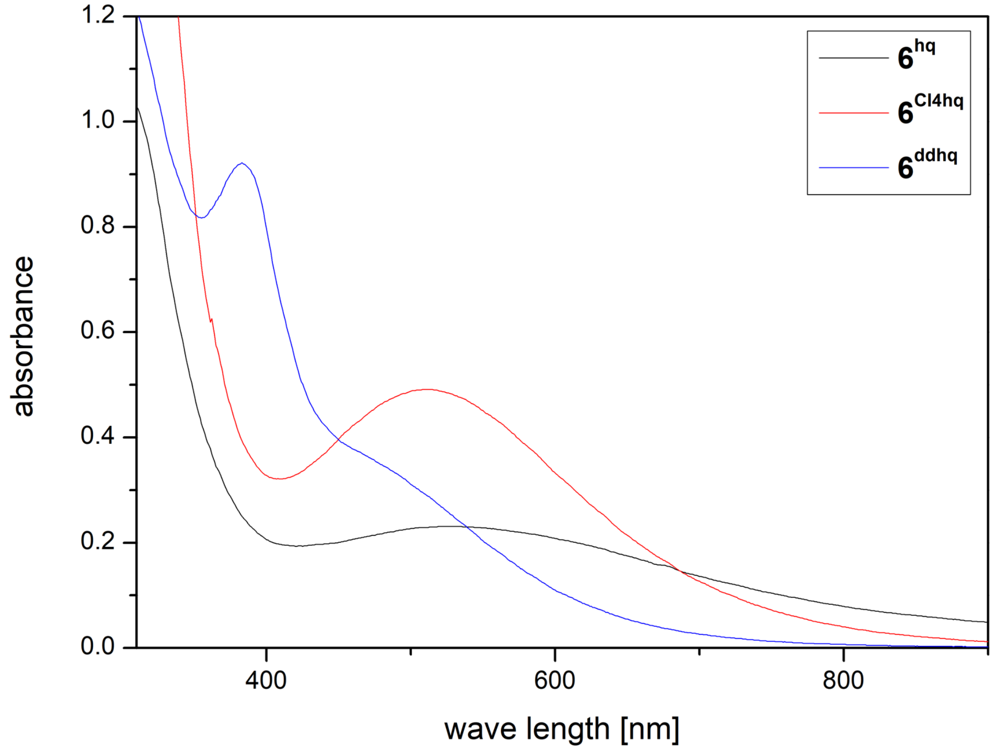

Figure S70. UV/Vis spectra of **6**^{hq}, **6**^{Cl4hq} and **6**^{ddhq} in toluene at ambient temperature. Due to its insolubility in toluene, **6**^{hq} was measured in THF.

EPR Spectra

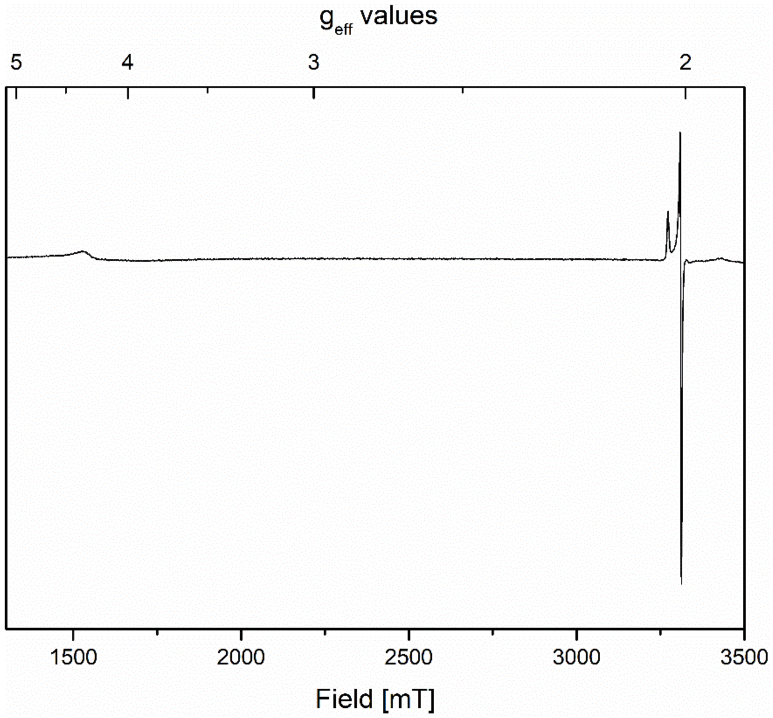

Figure S71. X-Band cw-EPR spectrum of crystalline [Ce{OSi(O*t*Bu)$_3$}$_2$(thf)$_2$(μ_2-O$_2$C$_6$Me$_4$)]$_2$ (**8**) (microwave frequency = 9.315802 GHz, microwave power = 0.6325 mW, modulation amplitude = 0.2 mT, T = 123 K).

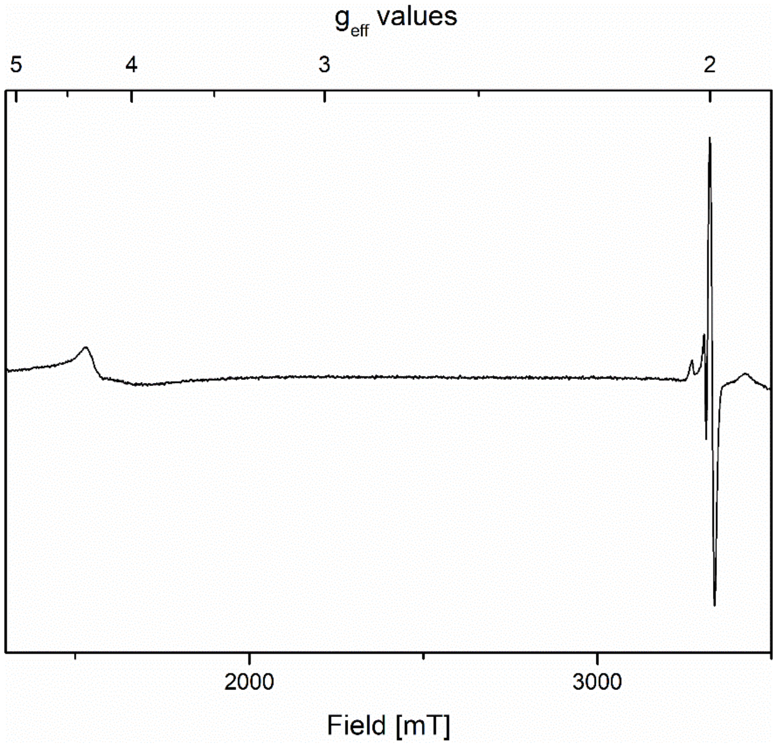

Figure S72. X-Band cw-EPR spectrum of crystalline [Ce(OSi*i*Pr$_3$)$_2$(thf)$_2$)(μ_2-O$_2$C$_6$Me$_4$)]$_2$ (**9**) (microwave frequency = 9.313522 GHz, microwave power = 0.6325 mW, modulation amplitude = 0.5 mT, T = 123 K).

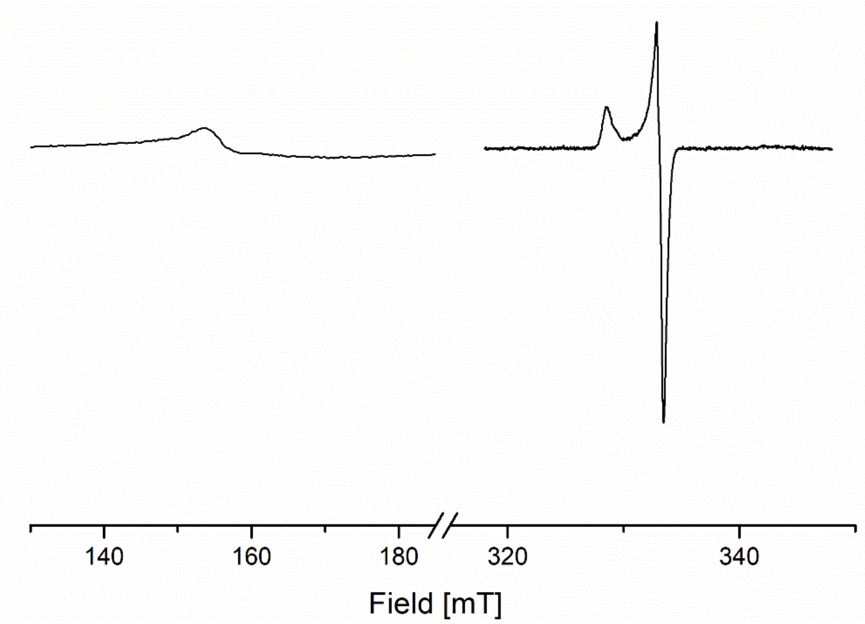

Figure S73. X-Band cw-EPR spectrum of $[Ce\{OSi(O\textit{t}Bu)_3\}_3]_2$ (**2**) in a frozen 2-Me-THF solution. Signal at 150 mT: microwave frequency = 9.37951 GHz, microwave power = 0.1262 mW, modulation amplitude = 0.06 mT, T = 77 K; Signal at 330 mT: microwave frequency = 9.380779 GHz, microwave power = 0.1262 mW, modulation amplitude = 0.5 mT, T = 77 K.

Crystallography

Crystals for X-ray crystallography were grown using saturated solutions of *n*-hexane (**3**), mixtures of *n*-hexane/toluene (**4**$^{\mathbf{Cl4hq}}$, **4**$^{\mathbf{ddhq}}$, **4**$^{\mathbf{Me4hq}}$, **4**$^{\mathbf{tBuhq}}$, **4**$^{\mathbf{nhq}}$), THF (**5**$^{\mathbf{Cl4hq}}$, **6**$^{\mathbf{Cl4hq}}$, **6**$^{\mathbf{ddhq}}$, **6**$^{\mathbf{tBuhq}}$, **8**, **9**), THF-d_8 (**7**) or 2-methyltetrahydrofuran (**5**$^{\mathbf{hq}}$), toluene (**[Ce$_3$(bpad)(pasq)(Me$_2$pz)$_6$(thf)]**), THF/*n*-hexane mixture (**[Ce(Me$_2$pz)$_2$(thf)$_2$(asq)]$_2$**), or cyclohexane/toluene mixture (**[Ce(*t*Bu$_2$pz)$_3$]$_2$(μ_2-O$_2$C$_6$Me$_4$)**). Note: crystals of **5**$^{\mathbf{hq}}$ weren´t of sufficient quality for structural characterisation, but crystals suitable for X-ray diffraction analysis were obtained from 2-methyl-THF giving [(Ce{OSi(O*t*Bu)$_3$}$_3$(2-Methf))$_2$(μ_2-O$_2$C$_6$H$_4$)] (**5**$^{\mathbf{hq}}$**·2MeTHF**).Suitable crystals for X-ray analysis were handpicked in a glovebox, coated with Parabar 10312 and stored on microscope slides. Data collection was done on a *Bruker* APEX II Duo diffractometer by using QUAZAR optics and Mo K_α (λ = 0.71073 Å). The data collection strategy was determined using COSMO[9] employing ω scans. Raw data were processed by APEX[10] and SAINT,[11] corrections for absorption effects were applied using SADABS.[12] The structures were solved by direct methods and refined against all data by full-matrix least-squares methods on F^2 using SHELXTL[12] and SHELXLE or OLEX2.[13,14] All non-hydrogen atoms were refined anisotropically. The disorders of the solvent molecules or *tert*-butyl groups were modelled using DSR,[15] a program to refinement of disordered structures with SHELXL. Plots were generated by using CCDC Mercury 3.19.1.[16] Further details regarding the refinement and crystallographic data are listed in Table S11 and in the CIF files. Highly disordered solvent within the lattice, identified as toluene (**4**$^{\mathbf{ddhq}}$) and THF (**7**) were removed through application of the SQUEEZE program suite in PLATON.[17]

Crystallographic Data (CCDC#)

Table S11. Crystallographic data for compounds **3**, **4^{Cl4hq}**, **4^{ddhq}** and **4^{Me4hq}**

	**3**	**4^{Cl4hq}**	**4^{ddhq}**	**4^{Me4hq}**
**CCDC**	2022461	2022465	2022472	2022473
**formula**	$C_{54}H_{126}Ce_2O_6Si_6$	$C_{42}H_{108}Ce_2Cl_4N_6O_2Si_{12}$	$C_{44}H_{108}Ce_2Cl_2N_8O_2Si_{12}$	$C_{46}H_{120}Ce_2N_6O_2Si_{12}$
**M [g·mol^{-1}]**	1320.32	1488.46	1469.60	1406.79
**λ [Å]**	0.71073	0.71073	0.71073	0.71073
**colour**	colourless	brown	black	brown
**crystal dimensions [mm]**	0.341 × 0.315 × 0.223	0.20 × 0.10 × 0.05	0.195 × 0.136 × 0.074	0.40 × 0.32 × 0.23
**crystal system**	monoclinic	monoclinic	monoclinic	monoclinic
**space group**	$P2_1/n$	$P2_1/n$	C2/c	C2/c
**a [Å]**	12.6609(5)	10.8680(16)	24.8116(11)	24.645(6)
**b [Å]**	23.0055(9)	16.755(2)	21.7940(10)	22.211(6)
**c [Å]**	12.7568(5)	20.149(3)	17.4764(8)	17.381(4)
**α [°]**	90	90	90	90
**β [°]**	109.4400(10)	90.235(4)	116.6590(10)	116.237(3)
**γ [°]**	90	90	90	90
**V [Å^3]**	3503.8(2)	3668.9(9)	8445.6(7)	8534(4)
**Z**	2	2	4	4
**F(000)**	1396	1540	3048	2952
**T [K]**	180(2)	150(2)	100(2)	150(2)
**ρ_{calcd} [g·cm^{-3}]**	1.251	1.347	1.156	1.095
**μ[mm^{-1}]**	1.424	1.601	1.330	1.252
**Data / restraints / parameters**	9459 / 0 / 355	7343 / 0 / 326	10463 / 12 / 361	7314 / 0 / 327
**Goodness of fit**	1.039	1.034	1.037	1.090
**R_1 (I > 2σ (I))[a]**	0.0256	0.0488	0.0289	0.0625
**$_{\omega}R_2$ (all data)[b]**	0.0631	0.0949	0.0688	0.1511

[a] $R_1 = \Sigma(||F0|-|Fc||)/\Sigma|F0|, F0 > 4s(F0)$. [b] $_{\omega}R_2 = \{\Sigma[w(F02-Fc2)2/\Sigma[w(F02)^2]]\}^{1/2}$.

Table S11 continued. Crystallographic data for compounds **4**^{tBu2hq}, **4**^{nhq}, **5**^{hq} and **5**^{Cl4hq}

	**4**^{tBu2hq}	**4**^{nhq}[c]	**5**^{hq}	**5**^{Cl4hq}
**CCDC**	2022468		2022470	2022476
**formula**	$C_{57}H_{136}Ce_2N_6O_2Si_{12}$	$C_{46}H_{114}Ce_2N_6O_2Si_{12}$	$C_{97.07}H_{204.13}Ce_2O_{30}Si_6$	$C_{90}H_{186}Ce_2Cl_4O_{29}Si_6$
**M [g·mol^{-1}]**	1555.03	1400.75	2300.34	2322.96
**λ [Å]**	0.71073	0.71073	0.71073	0.71073
**colour**	brown	brown	dark purple	purple
**crystal dimensions [mm]**	0.10 × 0.05 × 0.01		0.281 × 0.167 × 0.126	0.252 × 0.202 × 0.088
**crystal system**	triclinic	monoclinic	triclinic	orthorhombic
**space group**	$P\bar{1}$	P2/c	$P\bar{1}$	Pnna
**a [Å]**	11.4339(9)	20.343(7)	13.7919(11)	22.1584(19)
**b [Å]**	12.0881(10)	11.429(4)	14.0585(12)	41.418(4)
**c [Å]**	17.5236(14)	20.112(7)	20.0654(16)	13.4250(12)
**α [°]**	83.3194(13)	90	71.904(2)	90
**β [°]**	71.7094(12)	117.499(7)	81.904(2)	90
**γ [°]**	63.9637(11)	90	60.636(2)	90
**V [Å³]**	2065.6(3)	4148(2)	3222.7(5)	12320.8(19)
**Z**	1		1	4
**F(000)**	820		1227	4904
**T [K]**	150(2)		100(2)	100(2)
**ρ_{calcd} [g·cm^{-3}]**	1.250		1.185	1.252
**μ[mm^{-1}]**	1.300		0.815	0.937
**Data / restraints / parameters**	8433 / 122 / 411		18242 / 307 / 710	13600 / 1893 / 896
**Goodness of fit**	1.004		1.021	1.108
**R_1 (I > 2σ (I))[a]**	0.0316	0.1508	0.0269	0.0527
**$_{\omega}R_2$ (all data)[b]**	0.0677	0.3537	0.0681	0.1280

[a] $R_1 = \Sigma(||F0|-|Fc||)/\Sigma|F0|, F0 > 4s(F0)$. [b] $_{\omega}R_2 = \{\Sigma[w(F02-Fc2)2/\Sigma[w(F02)^2]]\}^{1/2}$. [c] Connectivity only.

Table S11 continued. Crystallographic data for compounds **6**^{Cl4hq}, **6**^{ddhq}, **6**^{tBu2hq} and **7**

	**6**^{Cl4hq}	**6**^{ddhq}	**6**^{tBu2hq}	**7**
**CCDC**	2022471	2022467	2022466	2022474
**formula**	$C_{76}H_{158}Ce_2Cl_4O_{12}Si_6$	$C_{70}H_{142}Ce_2Cl_2N_2O_{10}Si_6$	$C_{76}H_{162}Ce_2O_{10}Si_6$	$C_{66}H_{132}Ce_2Co_2D_8N_6O_3Si_{12}$
**M [g·mol^{-1}]**	1854.59	1691.53	1684.83	1809.06
**λ [Å]**	0.71073	0.71073	0.71073	0.71073
**colour**	orange	orange	purple	brown
**crystal dimensions [mm]**	0.137 × 0.130 × 0.056	0.191 × 0.169 × 0.102	0.270 × 0.098 × 0.034	0.142 × 0.131 × 0.082
**crystal system**	monoclinic	monoclinic	triclinic	monoclinic
**space group**	$P2_1/c$	$C2/c$	$P\bar{1}$	$P2/n$
**a [Å]**	11.2124(10)	18.228(5)	11.655(2)	11.7272(6)
**b [Å]**	12.5614(11)	17.588(4)	12.402(2)	20.2947(10)
**c [Å]**	34.289(3)	28.531(7)	17.499(3)	21.6422(11)
**α [°]**	90	90	92.596(3)	90
**β [°]**	97.212(2)	102.369(4)	93.946(3)	99.9900(10)
**γ [°]**	90	90	114.855(3)	90
**V [Å^3]**	4791.2(7)	8935(4)	2281.9(7)	5072.7(4)
**Z**	2	4	1	2
**F(000)**	1956	3560	898	1880
**T [K]**	101(2)	100(2)	100(2)	100(2)
**ρ_{calcd} [g·cm^{-3}]**	1.286	1.258	1.226	1.184
**μ[mm^{-1}]**	1.174	1.194	1.111	1.380
**Data / restraints / parameters**	12413 / 412 / 527	13667 / 138 / 495	10190 / 0 / 446	12529 / 214 / 501
**Goodness of fit**	1.015	1.096	1.027	1.010
**R_1 (I > 2σ (I))[a]**	0.0403	0.0459	0.0452	0.0395
**$_{\omega}R_2$ (all data)[b]**	0.0801	0.1268	0.0934	0.0863

[a] $R_1 = \Sigma(||F0|-|Fc||)/\Sigma|F0|, F0> 4s(F0)$. [b] $_{\omega}R_2 = \{\Sigma[w(F02-Fc2)2/\Sigma[w(F02)^2]]\}^{1/2}$.

Table S11 continued. Crystallographic data for compounds **8**, **9**, **[Ce$_3$(bpad)(pasq)(Me$_2$pz)$_6$(thf)]**, **[Ce(Me$_2$pz)$_2$(thf)$_2$(asq)]$_2$** and **[Ce(*t*Bu$_2$pz)$_3$(thf)]$_2$(*μ*$_2$-O$_2$C$_6$Me$_4$)**

	**8**	**9**	**[Ce$_3$(bpad)(pasq)(Me$_2$pz)$_6$(thf)]**	**[Ce(Me$_2$pz)$_2$(thf)$_2$(asq)]$_2$**	**[Ce(*t*Bu$_2$pz)$_3$(thf)]$_2$(*μ*$_2$-O$_2$C$_6$Me$_4$)**
**CCDC**	2022469	2022475	2022464	2022462	2022463
**formula**	$C_{100}H_{196}Ce_2O_{28}Si_4$	$C_{72}H_{140}Ce_2O_{12}Si_4$	$C_{91}H_{103}Ce_3N_{18}O_5$	$C_{68}H_{84}Ce_2N_8O_9$	$C_{84}H_{142}Ce_2N_{12}O_4$
**M [g·mol^{-1}]**	2239.16	1590.43	1949.27	1437.67	1664.33
**λ [Å]**	0.71073	0.71073	0.71073	0.71073	0.71073
**colour**	clear blue	blue	clear colourless	turquoise	reddish brown
**crystal dimensions [mm]**	0.220 × 0.120 × 0.010	0.209 × 0.110 × 0.076	0.312 × 0.221 × 0.122	0.210 × 0.130 × 0.020	0.100 x 0.020 x 0.010
**crystal system**	monoclinic	monoclinic	triclinic	triclinic	monoclinic
**space group**	$P2_1/c$	$P2_1/n$	$P\bar{1}$	$P\bar{1}$	$P2_1/n$
**a [Å]**	20.8578(10)	11.29(4)	12.7863(16)	11.8590(14)	11.7195(4)
**b [Å]**	24.2951(10)	22.03(7)	18.594(2)	14.7713(18)	18.0849(7)
**c [Å]**	11.7354(5)	17.25(7)	18.631(2)	19.126(2)	21.3488(8)
**α [°]**	90	90	102.5253(19)	79.676(2)	4505.1(3)
**β [°]**	94.4068(11)	96.250(11)	90.9634(18)	76.075(2)	90
**γ [°]**	90	90	93.3308(18)	81.398(2)	95.3481(15)
**V [Å^3]**	5929.2(5)	4265(27)	4314.7(9)	3179.2(7)	90
**Z**	2	2	2	2	2
**F(000)**	2384	1680	1978	1472	1756
**T [K]**	100(2)	100(2)	100(2)	100(2)	100(2)
**ρ_{calcd} [g·cm^{-3}]**	1.254	1.238	1.500	1.502	1.227
**μ[mm^{-1}]**	0.864	1.160	1.619	1.477	1.049
**Data / restraints / parameters**	10454 / 792 / 740	9414 / 0 / 423	17755 / 273 / 1199	20302 / 0 / 792	8250 / 138 / 520
**Goodness of fit**	1.064	1.100	0.968	0.996	1.071
**R_1 (I > 2σ (I))[a]**	0.0539	0.0502	0.0410	0.0442	0.0417
**$_{\omega}R_2$ (all data)[b]**	0.1005	0.0960	0.0759	0.1024	0.0749

[a] $R_1 = \Sigma(||F0|-|Fc||)/\Sigma|F0|, F0 > 4s(F0)$. [b] $_{\omega}R_2 = \{\Sigma[w(F02-Fc2)2/\Sigma[w(F02)^2]]\}^{1/2}$.

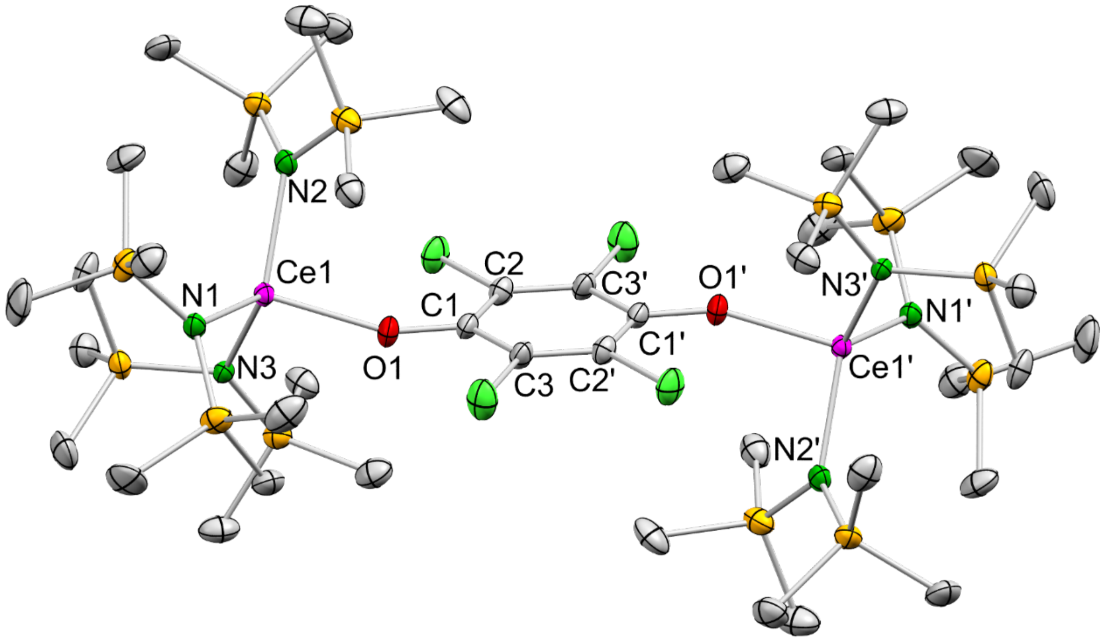

Figure S74. Crystal structure of [Ce{N(SiMe$_3$)$_2$}$_3$]$_2$(μ_2-O$_2$C$_6$Cl$_4$) (**4**^{Cl4hq}). Ellipsoids are shown at the 50% probability level. Hydrogen atoms and lattice toluene are omitted for clarity. Selected interatomic distances are listed in Table 1 (paper).

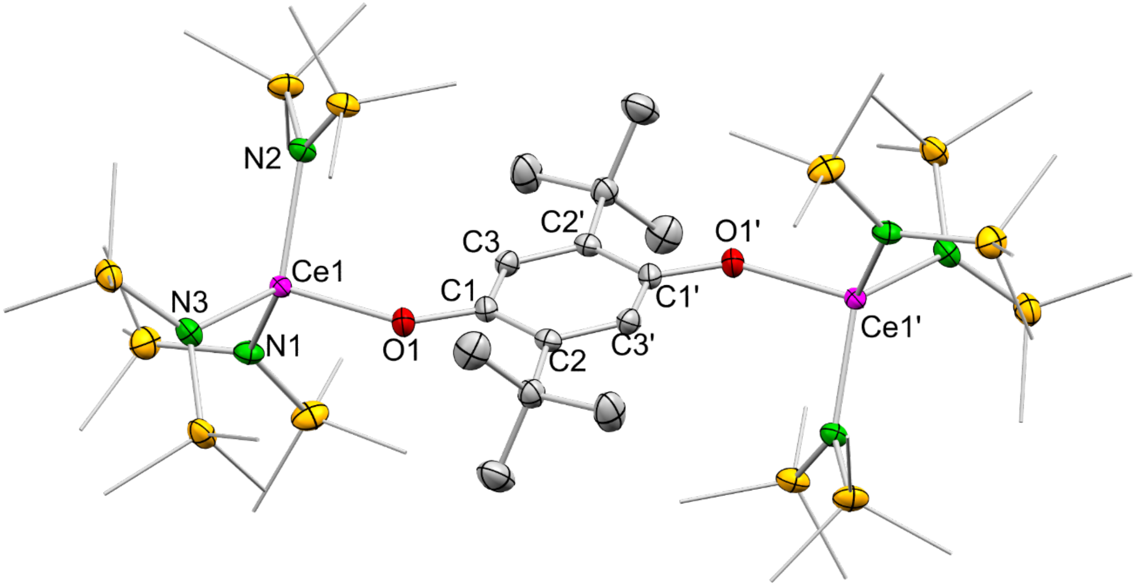

Figure S75. Crystal structure of [Ce{N(SiMe$_3$)$_2$}$_3$]$_2$(μ_2-O$_2$C$_6$*t*Bu$_2$H$_2$) (**4**^{tBu2hq}). Ellipsoids are shown at the 50% probability level. Hydrogen atoms and lattice toluene are omitted for clarity. Selected interatomic distances are given in Table 1 (paper).

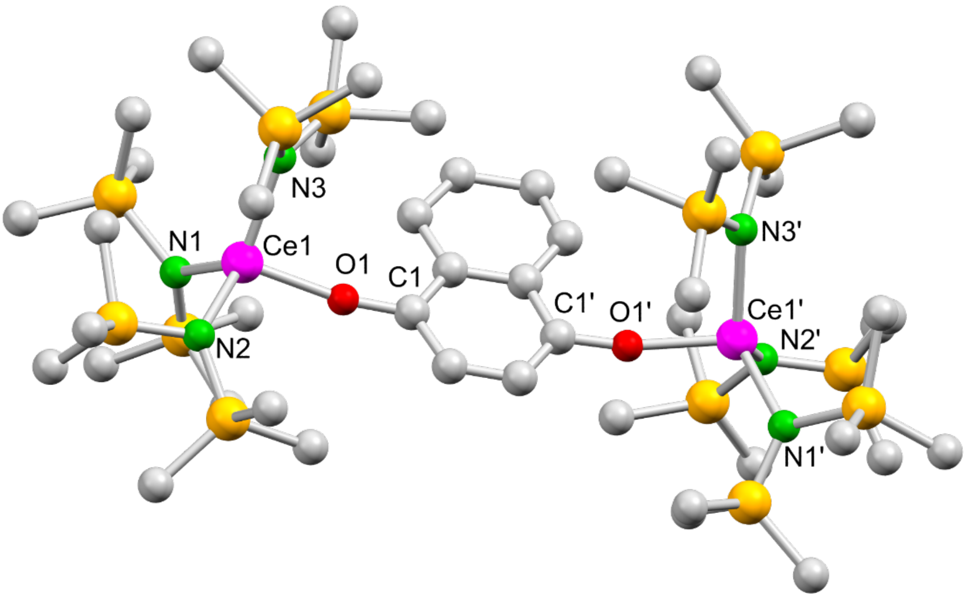

Figure S76. Connectivity of $[Ce\{N(SiMe_3)_2\}_3]_2(\mu_2\text{-}O_2C_6tBu_2H_2)$ (**4**^{nhq}). Hydrogen atoms and disordered atoms are omitted for clarity.

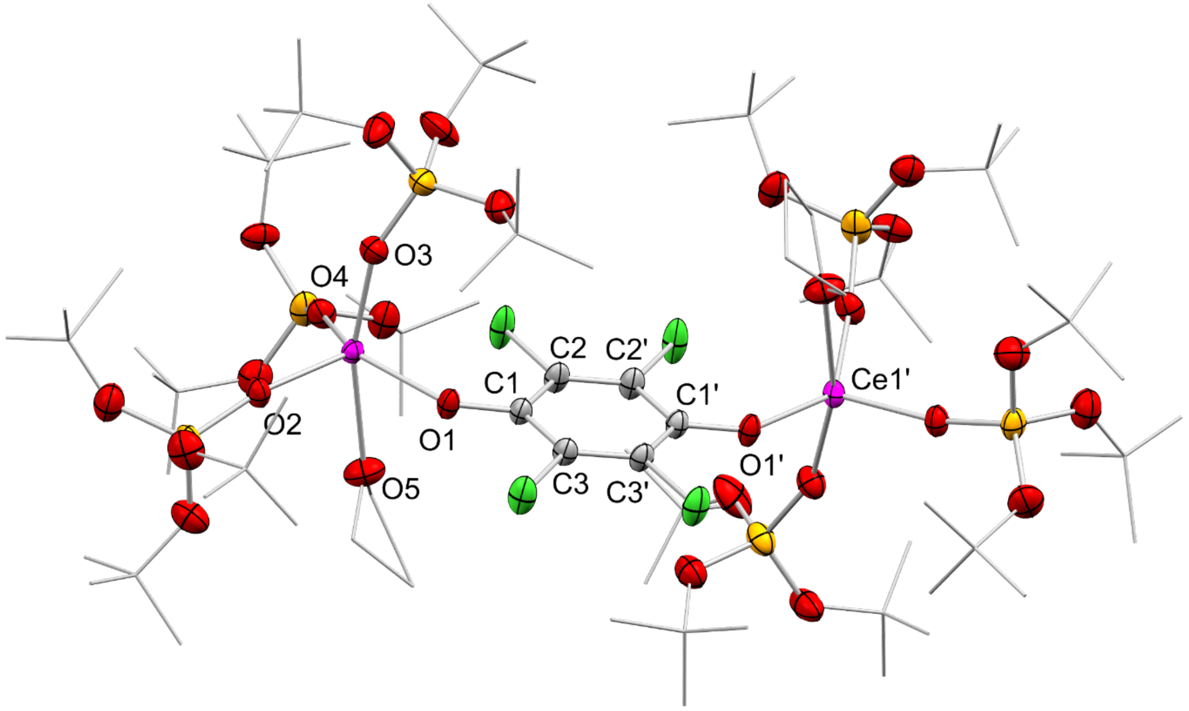

Figure S77. Crystal structure of $[Ce\{OSi(OtBu)_3\}_3(thf)]_2(\mu_2\text{-}O_2C_6Cl_4)$ (**5**^{Cl4hq}). Ellipsoids are shown at the 50% probability level. Hydrogen atoms and lattice THF are omitted for clarity. Selected interatomic distances are listed in Table 1 (paper).

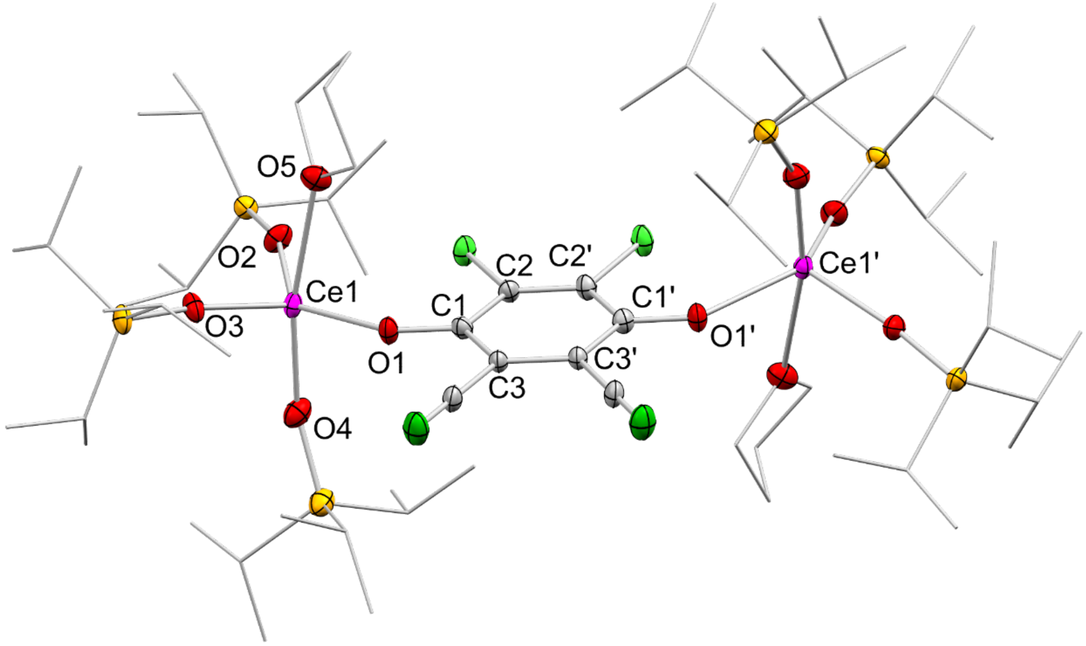

Figure S78. Crystal structure of [Ce(OSi*i*Pr$_3$)$_3$(thf)]$_2$(μ_2-O$_2$C$_6$Cl$_2$(CN)$_2$) (**6**$^{\mathbf{ddhq}}$). Ellipsoids are shown at the 50% probability level. Hydrogen atoms and lattice THF are omitted for clarity. Selected interatomic distances are given in Table 1 (paper).

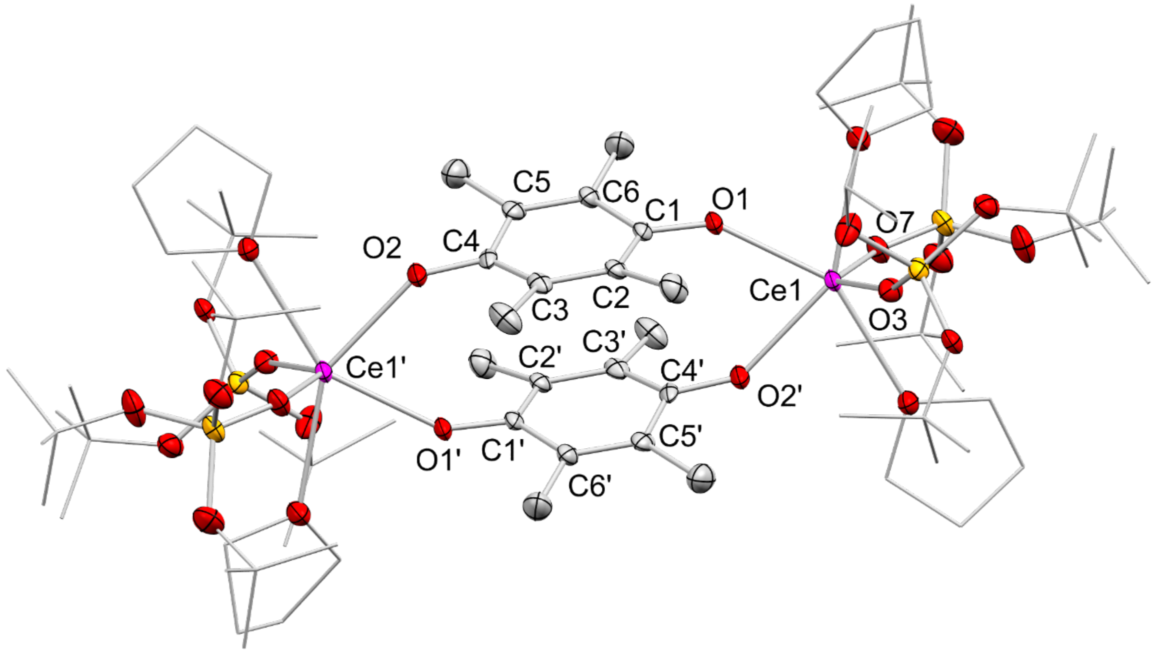

Figure S79. Crystal structure of [Ce{OSi(O*t*Bu)$_3$}$_2$(thf)$_2$(μ_2-O$_2$C$_6$Me$_4$)]$_2$ (**8**). Ellipsoids are shown at the 50% probability level. Hydrogen atoms and lattice THF are omitted for clarity. Selected interatomic distances [Å]: Ce1–O1 2.370(3), Ce1–O2 2.382(3), Ce1–O3 2.235(3), Ce1–O7 2.249(3), Ce1–O12 2.571(3), Ce1–O11 2.579(3),C1–O1 1.285(6), C4–O2 1.286(6), C1–C2 1.436(7), C1–C6 1.440(7), C2–C3 1.379(7), C3–C4 1.437(7), C4–C5 1.442(7), C5–C6 1.360(7).

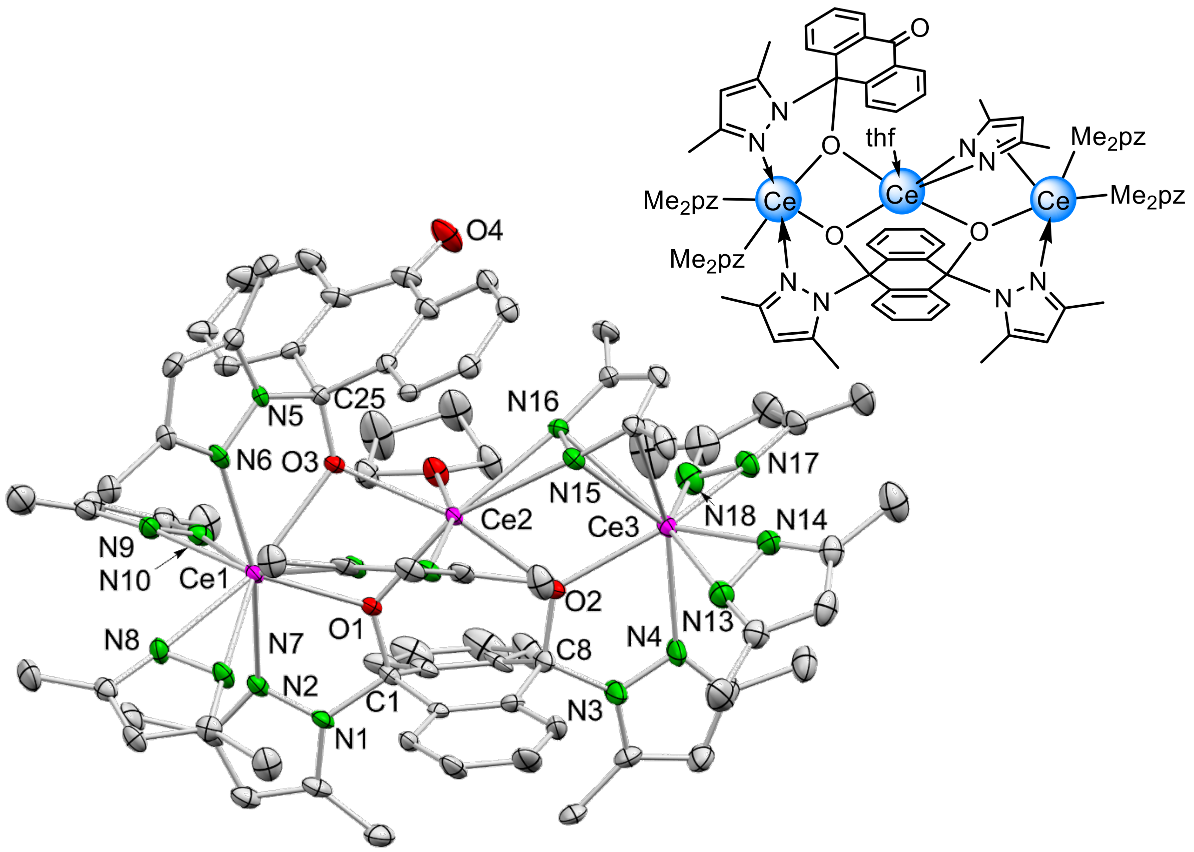

Figure S80. Crystal structure and structural representation of **[Ce$_3$(bpad)(pasq)(Me$_2$pz)$_6$(thf)]**. Ellipsoids are shown at the 50% probability level. Hydrogen atoms, lattice toluene and disordered atoms are omitted for clarity. Selected interatomic distances [Å]: Ce1–O1 2.488(3), Ce1–O3 2.532(2), Ce2–O1 2.379(2), Ce2–O2 2.495(3), Ce2–O3 2.421(3), Ce3–O2 2.429(3).

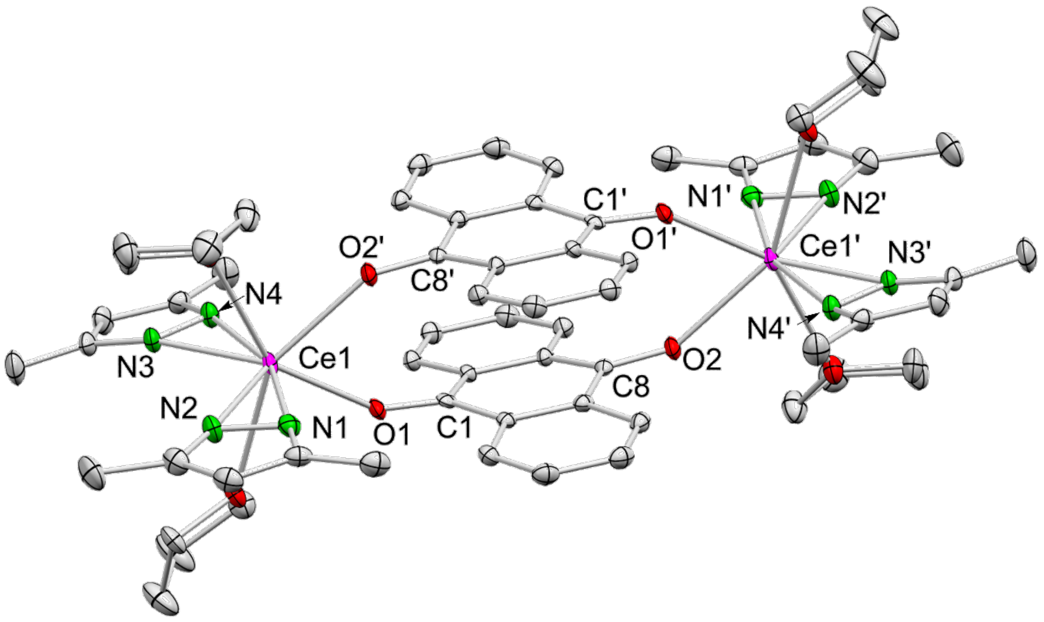

Figure S81. Crystal structure of **[Ce(Me$_2$pz)$_2$(thf)$_2$(asq)]$_2$**. Ellipsoids are shown at the 50% probability level. Hydrogen atoms, lattice THF and a second molecule are omitted for clarity. Selected interatomic distances [Å]: Ce1–O1 2.368(2), Ce1–O2' 2.365(2), Ce1–N1 2.510(2), Ce1–N2 2.462(3), Ce1–N3 2.468(2), Ce1–N4 2.504(2).

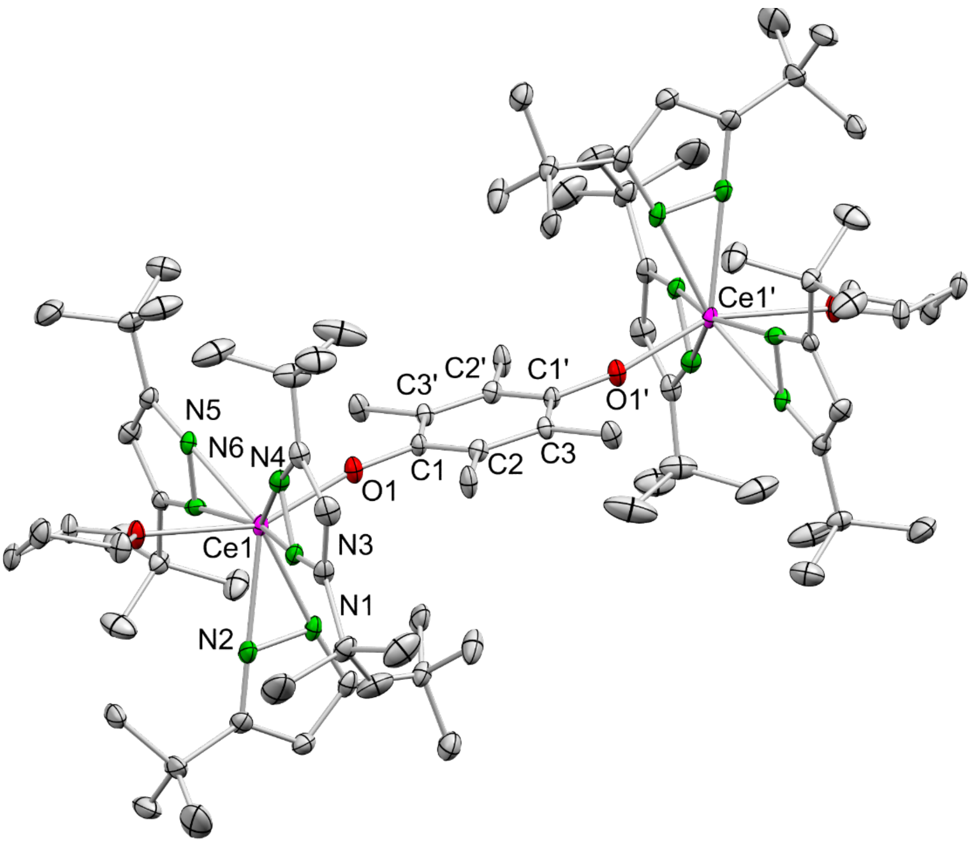

Figure S82. Crystal structure of and **[Ce(*t*Bu$_2$pz)$_3$]$_2$(μ_2-O$_2$C$_6$Me$_4$)**. Ellipsoids are shown at the 50% probability level. Hydrogen atoms and disordered atoms are omitted for clarity. Selected interatomic distances [Å]: Ce1–O1 2.076(2), Ce1–N1 2.339(3), Ce1–N2 2.408(3), Ce1–N3 2.377(3), Ce1–N4 2.409(3), Ce1–N5 2.412(3), Ce1–N6 2.362(3), C1–O1 1.357(4) C1–C2 1.409(5), C2–C3 1.391(5), C1–C3' 1.406(5).

References

1 P. B. Hitchcock, A. G. Hulkes, M. F. Lappert and Z. Li, *Dalton Trans.*, 2004, 129–136.
2 J. Friedrich, C. Maichle-Mössmer and R. Anwander, *Chem. Commun.*, 2017, **53**, 12044–12047.
3 D. Werner, G. B. Deacon, P. C. Junk and R. Anwander, *Chem. - Eur. J.*, 2014, **20**, 4426–4438.
4 D. Werner, U. Bayer, N. E. Rad, P. C. Junk, G. B. Deacon and R. Anwander, *Dalton Trans.*, 2018, **47**, 5952–5955.
5 R. K. Thomson, B. L. Scott, D. E. Morris and J. L. Kiplinger, *Comptes Rendus Chim.*, 2010, **13**, 790–802.
6 G. R. Fulmer, A. J. M. Miller, N. H. Sherden, H. E. Gottlieb, A. Nudelman, B. M. Stoltz, J. E. Bercaw and K. I. Goldberg, *Organometallics*, 2010, **29**, 2176–2179.
7 D. F. Evans, *J. Chem. Soc. Resumed*, 1959, 2003–2005.
8 Weast, R.C., *Handbook of Chemistry and Physics.*, CRC Press Inc, Boca Raton, Florida, 60th edn., 1979.
9 *COSMO, v. 1.61*, Bruker AXS Inc., Madiso, Wi, 2012.
10 *APEX 3, v. 2016.5-0*, Bruker AXS Inc., Madison, Wi, 2012.
11 *Saint, v. 8.34A*, Bruker AXS Inc., Madison, Wi, 2012.
12 L. Krause, R. Herbst-Irmer, G. M. Sheldrick and D. Stalke, *J. Appl. Crystallogr.*, 2015, **48**, 3–10.
13 G. M. Sheldrick, *Acta Crystallogr. Sect. Found. Adv.*, 2015, **71**, 3–8.
14 O. V. Dolomanov, L. J. Bourhis, R. J. Gildea, J. a. K. Howard and H. Puschmann, *J. Appl. Crystallogr.*, 2009, **42**, 339–341.
15 D. Kratzert, J. J. Holstein and I. Krossing, *J. Appl. Crystallogr.*, 2015, **48**, 933–938.
16 C. F. Macrae, I. J. Bruno, J. A. Chisholm, P. R. Edgington, P. McCabe, E. Pidcock, L. Rodriguez-Monge, R. Taylor, J. van de Streek and P. A. Wood, *J. Appl. Crystallogr.*, 2008, **41**, 466–470.
17 A. I. Spek, *Acta Crystallogr. Sect. C*, 2015, **71**, 9–18.
